# Peptide Ligands of AmiA, AliA, and AliB Proteins Determine Pneumococcal Phenotype

**DOI:** 10.3389/fmicb.2018.03013

**Published:** 2018-12-05

**Authors:** Fauzy Nasher, Fernando Aguilar, Suzanne Aebi, Peter W. M. Hermans, Manfred Heller, Lucy J. Hathaway

**Affiliations:** ^1^Institute for Infectious Diseases, Faculty of Medicine, University of Bern, Bern, Switzerland; ^2^Graduate School for Cellular and Biomedical Sciences, University of Bern, Bern, Switzerland; ^3^Janssen Vaccines and Prevention, Leiden, Netherlands; ^4^Julius Center, UMC Utrecht, Utrecht, Netherlands; ^5^Proteomics and Mass Spectrometry Core Facility, Department for BioMedical Research (DBMR), University of Bern, Bern, Switzerland

**Keywords:** *Streptococcus pneumoniae*, interspecies communications, growth, biofilm, capsule, chaining, ABC transporters

## Abstract

The Ami-AliA/AliB oligopeptide permease of *Streptococcus pneumoniae* has been suggested to play a role in environmental sensing and colonisation of the nasopharynx by this human bacterial pathogen by binding peptides derived from bacterial neighbours of other species in the microbiota. Here, we investigated the effects of the peptide ligands of the permease’s substrate binding proteins AmiA, AliA, and AliB on pneumococcal phenotype. AmiA and AliA ligands reduced pneumococcal growth, increased biofilm production and reduced capsule size. In contrast, AliB ligand increased growth and greatly increased bacterial chain length. A decrease in transformation rate was observed in response to all three peptides. Changes in protein expression were also observed, particularly those associated with metabolism and cell wall synthesis. Understanding interspecies bacterial communication and its effect on development of colonising versus invasive phenotypes has the potential to reveal new targets to tackle and prevent pneumococcal infections.

## Introduction

*Streptococcus pneumoniae* (pneumococcus) is one of the most extensively studied microorganisms, but this opportunistic pathogen remains a major causative agent of several human diseases including otitis media, pneumonia, sepsis and bacterial meningitis. However, the pneumococcus is more often a harmless inhabitant of the human nasopharynx, a niche it shares with many other microorganisms. Sensing of these microbial neighbours has been proposed to occur by binding of short peptides of these other bacterial species by *S. pneumoniae* via oligopeptide binding proteins of ATP-binding cassette (ABC) transporters (Hathaway et al., 2014). ABC transporters are also of interest as virulence factors since mutations in some have resulted in a reduction in pneumonia and bacteraemia in mouse models (Polissi et al., 1998; Lau et al., 2001).

One ABC transporter, the Ami-AliA/AliB permease, has been suggested to play a role in uptake of environmental signals and in competence for genetic transformation (Claverys et al., 2000). One of these permease’s oligopeptide binding proteins, AliB, has also been shown to aid in colonisation of the nasopharynx in a mouse model (Kerr et al., 2004). Previously, it has been shown that peptides found in other bacterial species are recognised by homologs of AliB: AliB-like ORF1 and ORF2 in non-encapsulated *S. pneumoniae* and that binding of the proteins to their peptide ligands affected pneumococcal phenotype and gene expression (Hathaway et al., 2014; Nasher et al., 2018a).

For the three substrate binding proteins of the Ami-AliA/AliB permease, we have shown recently that AmiA binds peptide AKTIKITQTR, matching an amino acid sequence found in 50S ribosomal subunit protein L30; AliA binds peptide FNEMQPIVDRQ, matching an amino acid sequence found in 30S ribosomal protein S20 and AliB binds peptide AIQSEKARKHN, matching an amino acid sequence found in 30S ribosomal protein S20 (Nasher et al., 2018b). These Ami-AliA/AliB peptide ligands are found in multiple bacterial species in the class of Gammaproteobacteria which includes common colonisers of the nostrils and nasopharynx (Pettigrew et al., 2012; Biesbroek et al., 2014; Mika et al., 2015; Teo et al., 2015). We therefore speculate that the pneumococcus binds these peptides to sense the presence and composition of its bacterial neighbours and adapt by altering its phenotype.

We determined here the effects of binding of AmiA, AliA, and AliB to their ligands on phenotypes associated with colonisation or virulence in wild type strains and mutants lacking the substrate binding proteins. Effects were found on growth, capsule size, chaining, transformation rate, and biofilm as well as on the pneumococcal proteome.

## Materials and Methods

### Ethics Statement

The human nasopharyngeal epithelial cell line Detroit 562 (CCL-138 was obtained from the American Type Culture Collection (ATCC)).

Clinical isolates of *S. pneumoniae* were selected from two nationwide surveillance programmes (Mühlemann et al., 2003; Kronenberg et al., 2006), from anonymous donors and have been used in previous studies (Hathaway et al., 2004, 2012).

### Bacterial Culture and Strains

Strain D39 (serotype 2) pneumococci were used as the parental strain for the production of mutants. Construction of the individual, ΔAmiA, ΔAliA, ΔAliB, and triple mutant ΔAmiA*/*ΔAliA*/*ΔAliB was described previously (Alloing et al., 1994; Kerr et al., 2004). Clinical isolates 106.66 (serotype 6B) and 110.58 (non-encapsulated, classic lineage) (Hilty et al., 2014) were also used. Bacteria, stored at -80°C using Protect bacterial preservers (Technical Service Consultants, Heywood, United Kingdom), were grown on Columbia sheep blood agar (CSBA) plates at 37°C, 5% CO_2_. Overnight cultures were prepared with 3–10 colonies in 5 mL brain heart infusion broth (Becton Dickinson and Company, le Pont de Claix, France) containing 5% foetal calf serum (FCS; Biochrom KG, Berlin, Germany; BHI+FCS medium).

### Growth Assay

Strains were streaked onto CSBA plates and incubated at 37°C in a 5% CO_2_-enriched atmosphere overnight then sub-cultured in BHI+FCS medium to OD_600nm_ 0.5, centrifuged at 3000 ×*g* for 5 min and resuspended in 5 mL chemically defined medium (CDM) which contains 5.5 mM glucose (Schaffner et al., 2014). Growth was monitored in sterile flat-bottomed 96-well microtiter plates (Nunclon Surface, Nunc, Denmark) based on the method of Brewster (2003). In brief, 200 μL bacteria culture was grown per well at 37°C and OD_450nm_ measured every 30 min using a VersaMax microplate reader (Molecular Devices) over 22 h with or without peptide ligands (synthesised by PolyPeptide Group, Strasbourg, France). The plate was shaken automatically for 5 s before each reading. The problem of condensation affecting the readings was avoided by pre-treating the lids of the 96-well plates with 3 mL 0.05% Triton X-100 in 20% ethanol and allowing them to air-dry before use (Hathaway et al., 2012).

### Growth Competition Assay

D39 and its mutants ΔAmiA, ΔAliA, ΔAliB, and ΔAmiA/ΔAliA/ΔAliB were streaked onto CSBA plates and incubated at 37°C in a 5% CO_2_-enriched atmosphere overnight then subcultured in BHI+FCS medium to OD_600nm_ 0.5, centrifuged at 3000 ×*g* for 5 min and resuspended in 5 mL CDM. 250 μL bacterial culture (125 μL of each of the two strains being compared) was transferred to 4.75 mL CDM pre-warmed to 37°C. The initial inoculum was plated out to check that there was a 1:1 mixture of the two bacterial strains. Peptide ligands were added to a final concentration of 0.5 mg/mL, and the culture incubated to an OD_600nm_ 0.3. Serial dilutions in PBS were plated onto CSBA plates with and without antibiotics to differentiate between wild type (susceptible) and mutants (resistant) strains. ΔAmiA mutant was selected on 10 μg/mL of tetracycline, ΔAliB on 2 μg/mL of chloramphenicol, and both ΔAliA and triple mutant ΔAmiA/ΔAliA/ΔAliB on 1 μg/mL of erythromycin/mL. After overnight incubation, the numbers of colonies were counted and the colony forming units (CFU) for each strain calculated.

### FITC-Dextran Exclusion Assay

Capsule thickness was determined by measuring the zone of exclusion of FITC-dextran as described previously (Gates et al., 2004; Hathaway et al., 2012), using FITC-dextran of 2000 kDa (Sigma-Aldrich) with the following difference, bacteria were cultured overnight in BHI+FCS medium until OD_600nm_ = 0.5 then 100 μL subcultured into 2 mL of CDM with and without peptide ligands and grown to an OD_600nm_ = 0.3. After centrifuging at 3000 ×*g* for 5 min the pellet was resuspended in 500 μL of PBS. 10 μL bacterial suspension was mixed with 2 μL FITC-dextran (10 mg/mL in water), pipetted onto a microscope slide and a coverslip applied firmly. The slides were viewed using a Zeiss Axio Imager M1 fluorescence microscope with a 100× objective and photographed by a Zeiss AxioCam HRc camera. The images were converted to grayscale and analysed with UTHSCSA ImageTool for Windows v3.0 (University of Texas Health Science Center, San Antonio, TX, United States) software. A bright field image was also photographed for each fluorescent image recorded in order to count the number of bacteria. Each image contained between 8 and 150 bacteria, five images were counted per group in each of three independent experiments making a total of 15 images per group. Each data point in Figure 3B represents one image.

### Chain Formation

Bacteria were cultured overnight in BHI+FCS medium until OD_600nm_ = 0.5 then 100 μL sub-cultured into 2 mL of CDM with and without peptide ligands and grown to an OD_600nm_ = 0.3. After centrifuging at 3000 ×*g* for 5 min the pellet was resuspended in 500 μL of PBS, 25 μL of the suspension was put on microscope slides and Gram stained. The slides were viewed using a Zeiss Axio microscope with a 100× objective and photographed by a Zeiss AxioCam HRc camera. A bright field image was acquired to count the number of bacteria per chain. 5 to 78 bacterial chains were counted per image, five images were counted per group in each of three independent experiments making a total of 15 images per group. Each data point in Figure 4B represents one image.

### Adherence to Human Epithelial Cell Line

Adherence to the nasopharyngeal epithelial cell line Detroit 562 (CCL-138, from the ATCC) was performed as described previously (Hathaway et al., 2004) with the following differences: A total of 4 × 10^5^ cells/well were cultured in a 24-well tissue culture plate in Minimum Essential Medium+Earle’s salts (MEM) containing 10% FCS, 2 mM L-glutamine, 1.5 g/L sodium bicarbonate, 0.1 mM non-essential amino acids and 1 mM sodium pyruvate (Gibco, United Kingdom) until reaching complete confluence at 37°C in 5% CO_2_. *S. pneumoniae* strains D39 and its mutants as well as strains 106.66 and 110.58 were grown in BHI+FCS to the logarithmic phase OD_600nm_ = 0.4 and washed in MEM then resuspended to give 10^7^ bacteria in 0.5 mL MEM, which was added per well of washed Detroit cells. The bacteria were centrifuged onto the cells at 390 ×*g* for 5 min at room temperature. After 1 h incubation of the cells with the bacteria at 37°C and five washes with PBS, 200 μL of trypsin was then added to each well and incubated for 5 min in 37°C to detach the cells. Adhered bacteria were recovered, serially diluted in 0.85% NaCl and plated onto CSBA. CFU/mL was then calculated after an overnight incubation at 37°C in 5% CO_2_.

### Biofilm Assay

Biofilm assays were performed as previously described (Marks et al., 2012b) with the following modifications. A total of 4 × 10^5^ Detroit cells/well in MEM salts containing 10% FCS, 2 mM L-glutamine, 1.5 g/L sodium bicarbonate, 0.1 mM non-essential amino acids and 1 mM sodium pyruvate (Gibco, United Kingdom) were cultured in 24-well tissue culture plate until reaching complete confluence at 37°C in 5% CO_2_. Strains D39 and its mutants and strains 106.66 and 110.58 were grown in CDM to OD_600nm_ 0.4 to give 10^5^ bacteria in 0.5 mL per well. 5 μL salmon sperm DNA was added into each well and, where indicated, corresponding peptide ligands, followed by 16 h of static incubation at 37°C, 5% CO_2_. The medium containing the planktonic bacteria was removed and each well was washed twice with PBS taking care not to disturb the biofilm. 200 μL PBS was then added per well and the plate was sonicated for 6 s in a water bath and then the biofilm scraped thoroughly from the wells. The contents of the wells were recovered and the CFU determined by plating out serial dilutions CSBA and incubating overnight at 37°C.

### Transformation Assay

To determine transformation rates of strain D39 and its mutants, strains 106.66 and 110.58, bacteria were grown to an OD_600nm_ = 0.15 in BHI+FCS. A total of 0.5 mL of the culture was transferred to 9.5 mL CDM prewarmed to 30°C and incubated for 15 min at 30°C in a waterbath. Competence-stimulating peptide 1 (CSP 1; ERLSKFFRFILQRLL) for D39 and its mutants and CSP 2 (EMRISRIILDFLFLRKK) for 106.66 and 110.58 were added to final concentration 100 ng/mL with and without AmiA, AliA, and AliB peptide ligands to a final concentration of 0.5 mg/mL, the culture incubated for 15 min at 30°C. Chromosomal DNA, 1 μg, from streptomycin resistant strain 104.37 was added and the culture incubated for 60 min at 30°C, then 90 min at 37°C. Serial dilutions in PBS were plated onto CSBA plates with and without 200 μg/mL streptomycin. After overnight incubation, the numbers of colonies were counted and the transformation rate calculated.

### Proteomic Analysis

Strain D39 and its mutants were cultured overnight in BHI+FCS medium until OD_600nm_ = 0.5 then 100 μL sub-cultured into 10 mL of CDM and grown to an OD_600nm_ = 0.2, the culture was split into two 5 mL aliquots and 0.5 mg/mL of peptides were added to one sample and incubated for 15 min at 37°C. Both cultures were centrifuged for 5 min at 4000 ×*g* and the pellets were resuspended in 50 mM Tris–HCl and 8 M urea and sonicated. Sample processing, Liquid Chromatography Mass Spectrometry (LC-MS/MS) and data interpretation was essentially done as described previously (Engel et al., 2014) with the following changes. LC-MS/MS analysis was carried out on an UltiMate 3000 Nano LC coupled to an Orbitrap Fusion Lumos instrument (Thermo Fisher Scientific) acquiring full MS scans in the *m*/*z* range 400–1400 in the orbitrap at resolution 120,000 with AGC set to 4e5 and maximal ion injection time of 50 ms. Peptide precursors with charge 2–8 were fragmented once in the ion trap then excluded for 30 s. The ion trap settings were data-dependent MS2 cycle time of 3 s, isolation width of 1.6 *m*/*z*, fragmentation HCD mode with 30% normalised collision energy, AGC of 1e4 with maximal ion injection time of 35 ms. The LC-MS/MS data was processed with MaxQuant (version 1.5.4.1) using default settings for peak detection, strict trypsin cleavage rule allowing for up to three missed cleavages, variable oxidation on methionine and acetylation of protein N-termini with strict carbamidomethylation of cysteines. Match between runs was activated with a retention time window of 0.7 min. The fragment spectra were interpreted with the *S. pneumoniae* strain D39 (serotype 2) ensemble database (version gca_d39.ASM1436v1). LFQ intensities were used for data analysis with imputation of missing values from the low end of the LOG2 transformed intensity distribution of each LC-MS/MS run using Perseus (version 1.5.5.3) as suggested by Lazar et al. (2016).

### Statistics

For proteomics, differential protein expression analysis was performed with *t*-tests within Perseus, including a permutation-based false discovery rate estimation (cutoff at 5% and using S0 function set at 0.5) to correct for multiple testing. For other experiments two-way ANOVA or student *t-*tests were performed to obtain *p*-values using the software GraphPad Prism (Version 7, GraphPad Software, Inc.).

## Results

### Peptide Ligands Affect Pneumococcal Growth

*Streptococcus pneumoniae* strain D39 (serotype 2) and its mutants in which one or all three of the substrate-binding proteins have been deleted (ΔAmiA, ΔAliA, ΔAliB, and ΔAmiA/ΔAliA/ΔAliB) were grown in CDM in 96 well plates in the absence and presence (at concentrations of between 0.5 and 0.062 mg/mL) of AmiA, AliA, and AliB peptide ligands for 22 h.

**FIGURE 1:**
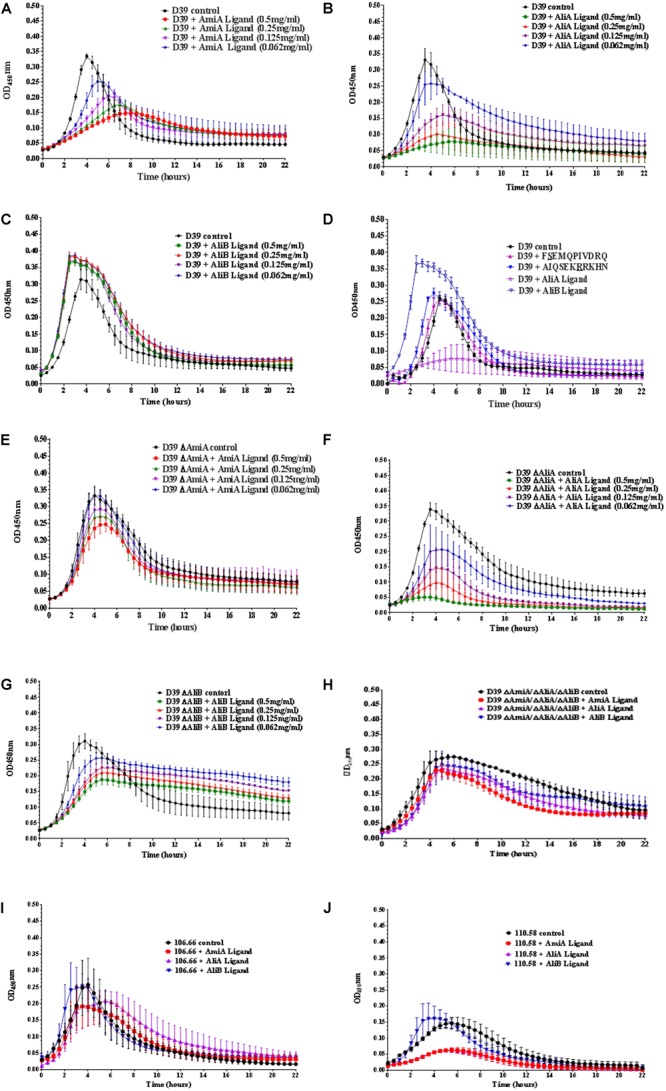
Growth curves of pneumococcal strain D39, its mutants and strains 106.66 and 110.58 in the presence and absence of the peptide ligands of AmiA, AliA, and AliB. Growth in chemically defined medium (CDM) was determined by measuring optical density over time for **(A)** wild type strain D39 in the absence (control) and presence of AmiA peptide ligand (at the different concentrations indicated), **(B)** D39 in the absence and presence of AliA ligand, **(C)** D39 in the absence and presence of AliB ligand, showing that AmiA and AliA ligands cause significant and dose dependent suppression of growth but AliB ligand does not. The effects of peptides on growth were specific as shown in panel **(D)** where D39 was grown in the absence and presence (0.5 mg/mL) of peptides with a single amino acid substitution: FSEMQPIVDRQ differs from AliA ligand at the second amino acid and AIQSEKRRKHN differs from AliB ligand at the seventh amino acid (underlined). Growth curves in the presence of the unmodified ligands have been included to allow direct comparison. AmiA peptide had much less suppressive effect on growth of the mutant lacking the AmiA receptor, D39 ΔAmiA **(E)**. Whereas growth of the D39 ΔAliA mutant was suppressed by AliA peptide **(F)**. Unlike the wild type D39 strain, growth of the D39 ΔAliB mutant was reduced by the AliB peptide **(G)**. None of the three peptide ligands had a great effect on the triple mutant ΔAmiA/ΔAliA/ΔAliB (**H**). Suppression of growth by AmiA and AliA peptides but not AliB peptide was also seen in two clinical pneumococcal isolates: strain 106.66 (serotype 6B) **(I)** and non-encapsulated strain 110.58 **(J)** (Peptide concentrations of 0.5 mg/mL). Curves show the mean values of at least three independent experiments performed on different days, error bars equal SEM.

For strain D39, AmiA, and AliA peptide ligands reduced growth significantly in a dose dependent way (Figures 1A,B) whilst AliB peptide ligand slightly reduced the lag phase and increased the maximum optical density (OD) (Figure 1C). To check that these effects were peptide specific, D39 was grown in the presence of peptides with single amino acid differences from peptides AliA and AliB (Figure 1D). (FSEMQPIVDRQ differs from AliA ligand at the second amino acid and AIQSEKRRKHN differs from AliB ligand at the seventh amino acid, underlined; a single amino acid version of AmiA peptide was insoluble and so could not be used in the growth assay). A single amino acid substitution caused AliA peptide to lose its ability to suppress growth and the substitution in the AliB peptide reduced its ability to boost growth response to the peptide.

Less suppression of growth was seen by AmiA peptide in the ΔAmiA mutant supporting the idea that this is the specific receptor for the peptide (Figure 1E). In contrast, AliA peptide affects the ΔAliA mutant in the same way as it affects the D39 wild type as expected since AliA peptide is known to bind to AmiA receptor (Figure 1F; Nasher et al., 2018b). Unlike the wild type D39 strain, growth of the D39 ΔAliB mutant was reduced by the AliB peptide (Figure 1G). However, none of the three peptide ligands had a great effect on the triple mutant ΔAmiA/ΔAliA/ΔAliB indicating a redundancy of function between the three substrate binding proteins (Figure 1H).

To test whether the peptides had the same effect on growth for clinical isolates, we tested the effects on strain 106.66 (serotype 6B) and non-encapsulated strain 110.58 (Figures 1I,J). As for D39, AmiA and AliA peptide ligands reduced growth whilst AliB peptide ligand slightly shortened the lag phase for these clinical isolates.

AmiA and AliA peptides have a bacteriostatic, rather than bacteriolytic, effect as shown by plating out and counting CFU at different timepoints during exponential growth (Supplementary Figure S1).

**FIGURE 2:**
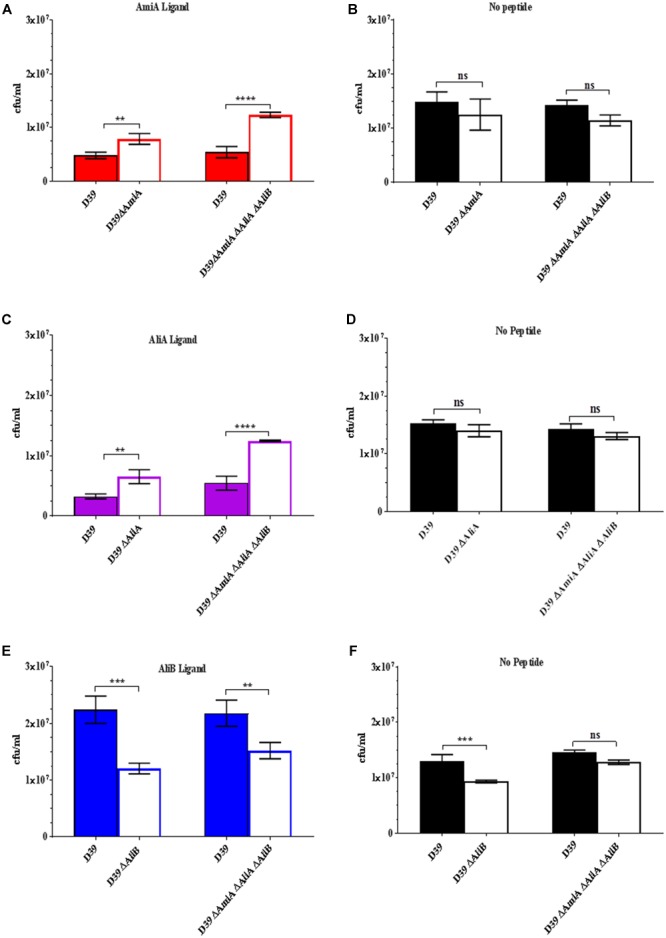
Competition assay between D39 and each of its mutants when cultured together in the absence and presence (0.5 mg/mL) of each of the peptides. Strain D39 was cultured together with mutant ΔAmiA or the triple mutant ΔAmiA/ΔAliA/ΔAliB in panel **(A)** the presence or **(B)** absence of AmiA ligand, with mutant ΔAliA or ΔAmiA/ΔAliA/ΔAliB in panel **(C)** the presence or **(D)** absence of AliA ligand and with mutant ΔAliB or the triple mutant ΔAmiA/ΔAliA/ΔAliB in panel **(E)** the presence or **(F)** absence of AliB ligand in CDM. When OD_600nm_ reached 0.3, the bacteria were plated onto CSBA with and without antibiotics to differentiate between the wild type and mutants and CFU counted. Results are as the mean ± SD of three independent experiments, ^∗∗^*p* = 0.0021; ^∗∗∗^*p* = 0.00021; ^∗∗∗∗^*p* < 0.0001; ns, not significant.

Given the effects on growth by the peptides in Figure 1, next we determined whether possession of the oligopeptide binding proteins affected the outcome of a competition assay when wild type strain D39 and each of its mutants were cultured together in the presence of the peptides. Figure 2A shows that D39 is outcompeted by both the mutant lacking AmiA (ΔAmiA) and the triple mutant ΔAmiA/ΔAliA/ΔAliB when the AmiA peptide is present but not when the AmiA peptide is absent (Figure 2B). The same pattern is seen for AliA (Figure 2C) with mutants lacking the receptor for the peptide having a growth advantage in the competition assay when the peptide was present (Figure 2C) but not when the peptide was absent (Figure 2D). In contrast, the wild type D39 strain had a competitive advantage over the mutant lacking AliB (ΔAliB) and over the triple mutant (Figure 2E). D39 also had a growth advantage over the ΔAliB mutant in the absence of peptide, although to a lesser extent, and no advantage over the triple mutant (Figure 2F).

(See Supplementary Figure S2 for all data points at OD_600nm_ = 0.1, 0.2, and 0.3).

The results of the competition assays support those of the growth curves: peptides AmiA and AliA inhibit growth but AliB enhances growth.

### Peptides Affect Capsule Thickness

**FIGURE 3:**
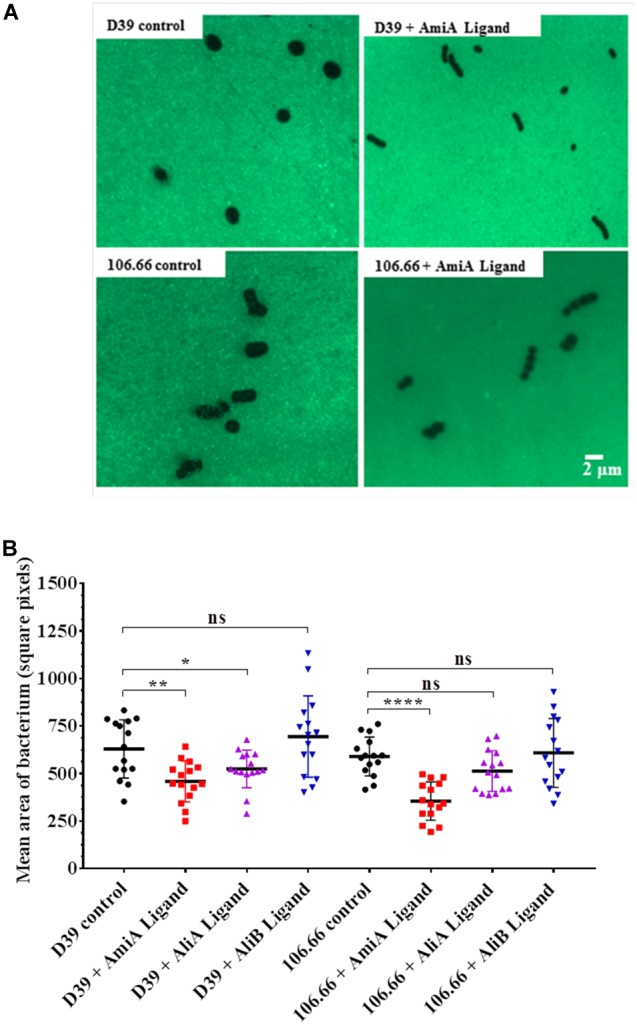
FITC-dextran exclusion assay to determine the effect of the peptides on capsule thickness. In CDM, bacteria were grown in the presence or absence of AmiA, AliA or AliB peptide ligands to OD_600nm_ = 0.2. **(A)** A representative fluorescent image shows a visible reduction in capsule size following incubation with AmiA peptide ligand for both strains D39 and 106.66. **(B)** Quantification of results with all peptides presented as mean ± SD of mean area per bacterium in square pixels. Each symbol represents one image containing between 8 and 150 bacteria. ^∗^*p* = 0.0332; ^∗∗^*p* = 0.002; ^∗∗∗∗^*p* = 0.0001; ns, not significant.

Since the polysaccharide capsule is a major virulence factor of *S. pneumoniae*, we investigated whether any of the three peptides affected capsule thickness. Figure 3A shows an example of a FITC-dextran exclusion assay using strains D39 (serotype 2) and 106.66 (serotype 6B). For both strains there was a clear diminution of capsule size following incubation with AmiA peptide. The results for all three peptides for both strains are summarised in Figure 3B. The reduction in capsule thickness in response to AmiA peptide was significant for both strains. AliA peptide also reduced capsule thickness in both strains, although the difference was only significant for strain D39. In contrast, AliB peptide ligand caused a slight, non-significant, increase in capsule thickness in strain D39 only (Figure 3B). None of the three peptide ligands affected capsule thickness significantly in the triple mutant ΔAmiA/ΔAliA/ΔAliB (Supplementary Figure S3). In the absence of peptide ligands, mutants had thinner capsule than the parent strain D39. AliA peptide ligand did not affect capsule thickness in the ΔAliA mutant, nor did AliB peptide ligand affect capsule thickness in the ΔAliB mutant. AmiA peptide significantly increased capsule thickness in the ΔAmiA mutant but only to a value equivalent to the capsule thickness of D39 in the absence of peptide (Supplementary Figure S3).

### Peptide Ligands Affect Chain Length of Encapsulated but Not Non-encapsulated Pneumococci

**FIGURE 4:**
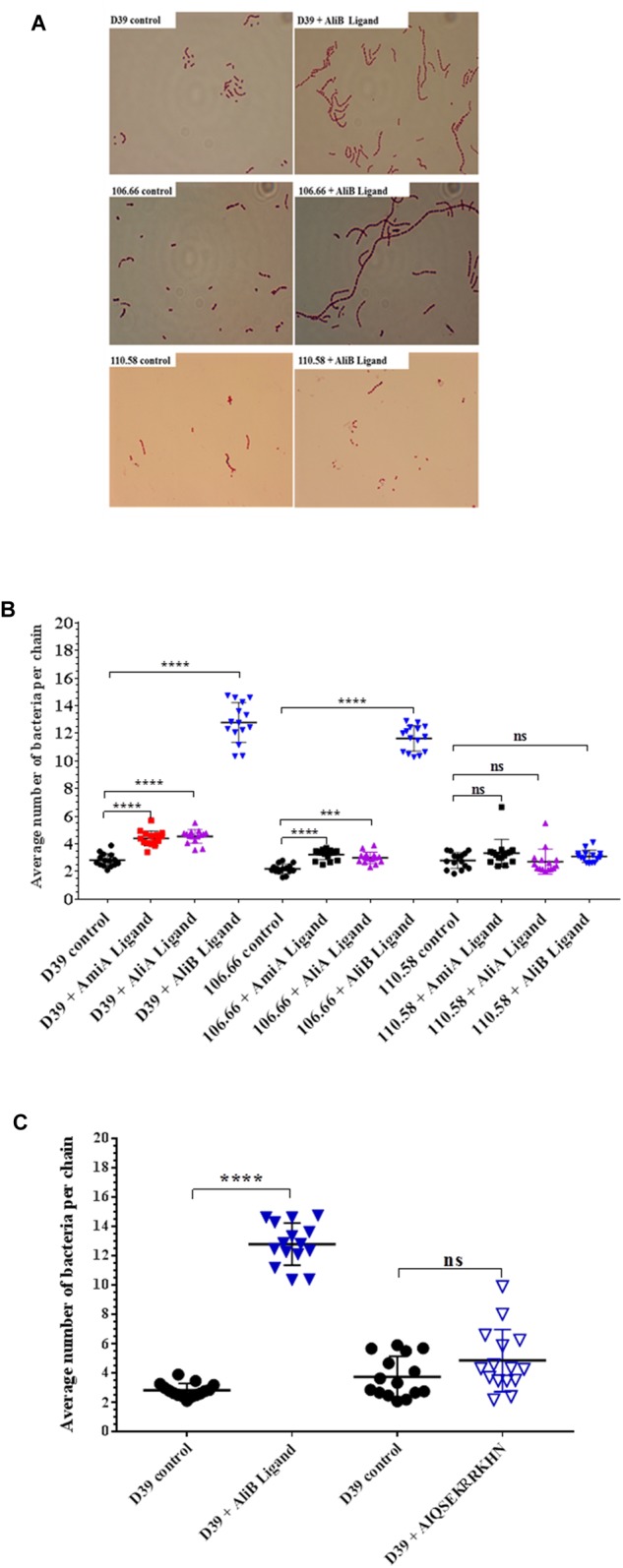
Effect of peptides on pneumococcal chain length. In CDM, strain D39 and its mutants, 106.66 and 110.58 were grown to OD_600nm_ = 0.2 in the absence and presence of peptide ligands (0.5 mg/mL). **(A)** Representative brightfield images showing differences in chain length between controls and *S. pneumoniae* treated with AliB peptide ligand. **(B)** Quantification of results with all peptides and **(C)** comparison of effect of AliB ligand with that of a peptide with a single amino acid substitution (AIQSEKRRKHN) on chain length of strain D39. Results are presented as mean ± SD of average number of bacteria per chain. Each symbol represents an image containing between 5 and 78 chains. ^∗∗∗^*p* = 0.00021; ^∗∗∗∗^*p* = 0.0001; ns, not significant.

When performing the capsule thickness experiments, we noticed that the peptide ligands appeared to affect chain length of the encapsulated pneumococci. We therefore quantified the effects of the peptide ligands on chain length (Figure 4). All three peptide ligands increased the average number of bacteria per chain, but by far the greatest effect was seen in presence of AliB peptide ligand on strains D39 and 106.66 (Figure 4A). The peptides increased chain length in the encapsulated strains D39 (serotype 2) and 106.66 (serotype 6B) but not the non-encapsulated strain 110.58 (Figure 4B). None of the three peptides had any significant effect on chain length in any of the four mutant strains (Supplementary Figure S4). By replacing the AliB peptide by the one with the single amino acid substitution (AIQSEKRRKHN) we show that the effect on chain length is specific to the AliB peptide (Figure 4C).

### Peptides Affect Biofilm Formation

**FIGURE 5:**
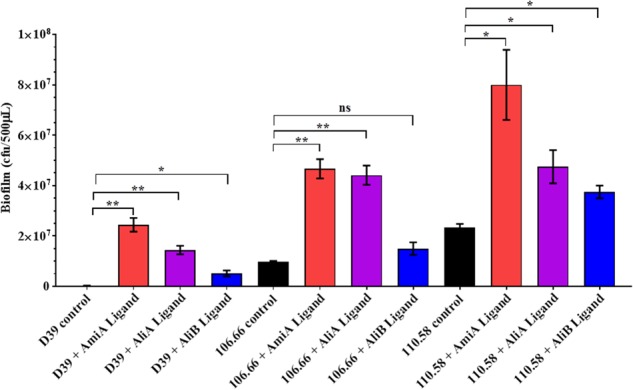
Effect of peptides on biofilm formation. Quantitative analysis of *in vitro* biofilm formation of strains D39, 106.66, and 110.58 on Detroit 562 nasopharyngeal epithelial cells in the absence and presence of peptide ligands (0.5 mg/mL) in CDM after 16 h of static incubation. Results are expressed as mean CFU/500 μL medium ± SD, ^∗^*p* = 0.0332; ^∗∗^*p* = 0.0021; ns, not significant.

We found no effect of the peptide ligands on adherence (see Supplementary Figure S5) but nevertheless we looked for an effect on biofilm formation. Peptide ligands for AmiA and AliA significantly increased biofilm formation for all three strains; D39, 106.66, and 110.58 (Figure 5). AliB peptide ligand caused a significant increase in biofilm in strains D39 and 110.58, although to a lesser extent than the other two peptides, but not in strain 106.66 (Figure 5). AmiA peptide increased biofilm production in mutant ΔAmiA/ΔAliA/ΔAliB but not in mutant ΔAmiA. AliA and AliB peptide ligands caused no changes in biofilm formation in any of the mutants tested (Supplementary Figure S6).

### Peptide Ligands Reduced Transformation Rate

**FIGURE 6:**
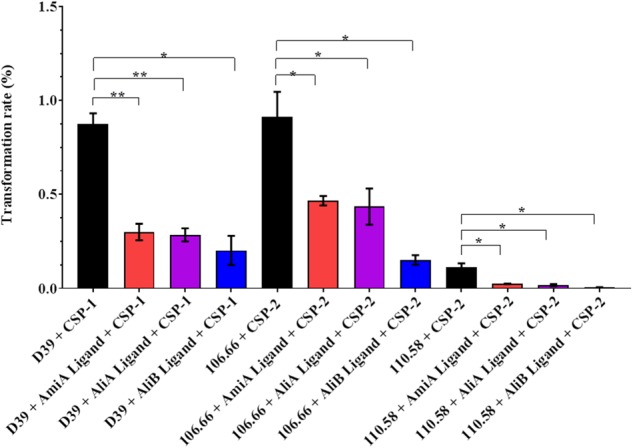
Effect of peptides on transformation rate. The rate of transformation to antibiotic resistance by the uptake of DNA from a resistant strain was determined for strains D39, 106.66, and 110.58 in the presence of the appropriate CSP (as indicated on the *x*-axis) and the absence and presence of the peptide ligands (0.5 mg/mL). Percentage transformation rates are the means ± SD of three independent experiments, ^∗^*p* = 0.0332; ^∗∗^*p* = 0.0021; ns, not significant.

*Streptococcus pneumoniae* is naturally competent for genetic transformation and being able to take up DNA in a hostile environment may confer a survival advantage. The effect of the peptides on CSP-mediated transformation rate was therefore tested in the strain D39, its mutants and strains 106.66 and 110.58. All three peptide ligands significantly reduced the transformation rate in strains D39, 106.66, and 110.58 (Figure 6). No significant effect was observed in the mutants (Supplementary Figure S7A). It must be noted that the peptide concentration is high (0.5 mg/mL) relative to the concentration of CSP (100 ng/mL) and that at such concentrations the AliA and AliB peptides with single amino acid substitutions and an irrelevant peptide reduced transformation rate (Supplementary Figure S7B), although in contrast to AmiA, AliA, and AliB, the results were not statistically significant.

### Peptide Affects Protein Expression

To gain insight into how the peptides caused these phenotype changes, we also looked at the effect of AmiA, AliA, and AliB peptide ligands on the pneumococcal proteome. Proteomic analysis was performed by LC-MS/MS on strain D39 and its mutants ΔAmiA, ΔAliA, ΔAliB, and triple mutant ΔAmiAΔAliAΔAliB in the presence and absence of peptide ligands. Table 1 shows all proteins significantly upregulated and downregulated in the wild type following a 15 min exposure to each of the peptide ligands. Although there was some overlap of the effect on protein expression between the three peptides, we noted that only AliB peptide increased expression of alanine racemase, a protein that catalyses the racemisation of L-alanine and D-alanine. D-alanine is needed for biosynthesis of peptidoglycan in the cell wall, essential for AliB peptide-induced growth. AmiA peptide appeared to switch off expression of several proteins associated with metabolism such as Vex2, the ATP-binding protein of an ABC transporter for export of lipoproteins during cell division, Cof family protein involved in metabolism and RecN, a protein that repairs DNA during replication. Two proteins uniquely switched off by AliA were a replicative DNA helicase and penicillin-binding protein 2X which is required for building cell wall. These results seem to support the role of AmiA and AliA proteins in reducing growth. Of interest was also the CinA protein, which is associated with competence and was switched on by AmiA and AliA but not AliB.

**Table 1:** All proteins significantly upregulated and downregulated in the wild type strain D39 following a 15 min exposure to each of the peptide ligands^∗^.

Protein ID	Name	Description	AmiA peptide	AliA peptide	AliB peptide
ABJ53612		Potassium uptake protein, Trk family protein		On	On
ABJ53738	mraW	S-adenosyl-methyltransferase MraW	On	On	On
ABJ54061	Alr	Alanine racemase			On
ABJ54079	cinA	Competence-damage protein, putative	On	On	
ABJ54125		Conserved hypothetical protein	On	On	On
ABJ54281		ABC transporter ATP-binding protein		On	On
ABJ54300	ispA	Geranyltranstransferase	Off		
ABJ54555		Conserved hypothetical protein	Off		Off
ABJ54708	scrB	Sucrose-6-phosphate hydrolase	Off	Off	Off
ABJ54728	dnaB	Replicative DNA helicase		Off	
ABJ54958	vex2	ABC transporter, ATP-binding protein Vexp2	Off		
ABJ54991		Cof family protein	Off		
ABJ55133	recN	DNA repair protein RecN	Off		
ABJ55135		Oxidoreductase, short chain dehydrogenase/reductase family protein	Off		Off
ABJ55333	pbpX	Penicillin-binding protein 2X		Off	
ABJ55410		Oxidoreductase, putative	Off		Off

*^∗^“On” corresponds to detection of the protein in the presence of the peptide specified and zero detection in the absence of the peptide. “Off” corresponds to detection of the protein in the absence of the peptide specified and zero detection in the presence of the peptide. For complete data values for D39 mutants see Supplementary Table S1.*

See Supplementary Table S1 for upregulated and downregulated proteins in the single and triple mutants in the presence of the peptides and Supplementary Table S2 for the complete set of proteins.

## Discussion

We propose that there is interspecies communication between bacteria that live in the human nasopharynx and that detecting other bacterial species allows *S. pneumoniae* to adapt to its environment. Previously, we reported that *S. pneumoniae* AmiA, AliA, and AliB lipoproteins of an ABC transporter are able to bind peptides matching ribosomal proteins of other bacteria species including common nasopharynx colonisers (Nasher et al., 2018b). These proteins are encoded by genes of the core pneumococcal genome (Obert et al., 2006) and so are of relevance across the pneumococcal population. Here, we looked at what effects these novel peptide ligands have on the phenotype of the pneumococcus.

In the presence of AmiA and AliA peptide ligands, growth of all strains was greatly reduced; by contrast AliB peptide ligand slightly boosted growth. Steady but persistent growth may be an ideal strategy for a coloniser in an environment such as the nasopharynx where nutrient availability is limited. In contrast proliferation of the pneumococcus has been suggested to increase the risk of invasive disease (Albrich et al., 2012).

AmiA and AliA peptides, but not AliB peptide, decreased capsule thickness. The polysaccharide capsule is the most important virulence factor of pneumococci, playing a role in invasive disease (Kelly et al., 1994; Beech et al., 2018). The capsule prevents phagocytosis mediated by Fcγ receptors, complement receptors and non-opsonic receptor thus protecting the pneumococci from the host immune response (Kelly et al., 1994; Hardy et al., 2001; Hammerschmidt et al., 2005; Hyams et al., 2010, 2013; Geno et al., 2015). Additionally, and an increase in capsule size has been observed in systemic infections (Kim and Weiser, 1998; Kim et al., 1999). However, capsule is downregulated when pneumococci are in intimate contact with epithelial cells (Hammerschmidt et al., 2005), and a transparent phenotype that is associated with improved colonisation of the nasopharynx has a reduced expression of capsular polysaccharide (Weiser, 2010). We therefore propose that when *S. pneumoniae* comes into contact with AmiA ligand and AliA ligand, these peptides signal the pneumococci to downregulate capsule expression which may promote attachment to epithelial cells, the first step in colonisation. Colonisation involves biofilm formation and, whilst all three peptides increased the amount of biofilm, the effect was greatest for AmiA and AliA peptides. Biofilm formation may provide several advantages for the pneumococci including a protective environment in which the bacteria can upregulate factors that are involved in nutrient uptake from their environment while downregulating virulence factors involved in inflammation to evade host defence system (Blanchette-Cain et al., 2013; Qin et al., 2013). Several previous studies have found that capsule size is inversely associated with biofilm formation (Hall-Stoodley et al., 2008; Marks et al., 2012b; Junges et al., 2017). In contrast, proliferating bacteria are able to transition from colonising to invasive disease at a higher degree than biofilm forming bacteria (Trappetti et al., 2011; Marks et al., 2013) suggesting that AliB peptide might have a higher likelihood than the other two peptides of being associated with invasion. We found that peptide ligands for AmiA and AliA particularly increased biofilm formation, despite not increasing adherence to epithelial cells in our *in vitro* model, although others have found a correlation between adherence and biofilm formation (Konkel et al., 1997; Marks et al., 2012a; Sulaeman et al., 2012). It is possible that the peptide ligands enhance biofilm formation by another mechanism such as enhancing extracellular matrix formation.

AliB peptide ligand dramatically increased chain formation and length in the encapsulated strains but not in the non-encapsulated 110.58. We propose that increasing chain length may compensate for a thick capsule during colonisation as having a thicker capsule has also been reported to restrict adherence to human epithelial cells (Talbot et al., 1996; Adamou et al., 1998; Ring et al., 1998; Novick et al., 2017). Non-encapsulated pneumococci have an advantage in adherence and colonisation (Keller et al., 2016), and may therefore not require increase in chaining for effective colonisation. If a longer chain allows more secure attachment as suggested by a recent report (Rodriguez et al., 2012), the chances of progression to invasive disease may also be greater.

Our findings indicate that *S. pneumoniae* transformation rate can be influenced by other bacterial species. To our knowledge, there have not been any reports on the effect of other bacterial species that are common colonisers of the nasopharynx on the transformation rate of pneumococcus, although there are several studies that highlight that competence and transformation rate of the pneumococcus is affected by co-colonisation of multiple serotypes (Donati et al., 2010; Hiller et al., 2010; Croucher et al., 2011; Muzzi and Donati, 2011; Marks et al., 2012b). Transformation rate was decreased by all three peptides, despite the finding that only AmiA and AliA reduced capsule thickness, but the only competence-associated protein found in the proteomic studies was CinA which was switched on by AmiA and AliA but not AliB. Expression of this protein has been previously shown to be turned on during competence (Martin et al., 1995) but its role in transformation has not been confirmed (Mortier-Barrière et al., 1998). Since biofilm formation is generally associated with increased competence, further studies over a time course might be warranted to decipher the precise role of the peptides in competence.

Proteomic data indicated downregulation of some proteins involved in metabolism or cell division by AmiA peptide (Vex2, Cof family protein, RecN) and AliA (DnaB, PbpX). These results are compatible with the phenotypic data, where AmiA and AliA reduced growth and AliB increased both peptidoglycan production (Alr) and increased growth. Alr is an alanine racemase and uses a covalently bound pyridoxal 5′-phosphate cofactor to catalyse the racemisation of L-alanine and D-alanine. D-alanine is a necessary component of the peptidoglycan layer in bacterial cell walls (Lambert and Neuhaus, 1972; Milligan et al., 2007). In *S. mutans*, Alr has also been shown to be necessary for growth and competitiveness (Wei et al., 2016). However, proteomic data was not clear cut, for example MraW, involved in cell wall biogenesis was increased by all three peptides,

A limitation in this study was the incubation time of the pneumococcal strains and the peptide ligands which only provided a snapshot of the proteome profile. We cannot overlook the fact that we could have also missed regulation of many other essential proteins thought to be involved in pneumococcal pathogenesis. In addition, all our studies investigated the effects of one peptide ligand for each lipoprotein receptor but we do not exclude the possibility that other peptides may bind and that their effects on the pneumococcal phenotypes may be different from those found in the present study.

We have shown that AmiA, AliA, and AliB peptides, found in ribosomal proteins of other bacterial species, alter the phenotypes of *S. pneumoniae*. We propose that exploiting this knowledge could offer new targets in the prevention and treatment of pneumococcal diseases.

## Author Contributions

LH conceived the study. FN wrote the first draft of the manuscript. FN, FA, and LH participated in design of the study. FN, SA, and FA performed the research. MH analyzed proteomic data. PH provided the strain D39 and mutants. All authors gave final approval for publication.

## Conflict of Interest Statement

PH is employed by the company Janssen Vaccines and Prevention. The remaining authors declare that the research was conducted in the absence of any commercial or financial relationships that could be construed as a potential conflict of interest.
