# Supplementary material for: Peptide Ligands of AmiA, AliA, and AliB Proteins Determine Pneumococcal Phenotype
Source: Front Microbiol. 2018 Dec 5;9:3013. doi: 10.3389/fmicb.2018.03013 (PMC6290326; doi:10.3389/fmicb.2018.03013)
Supplement: Supplementary file 1 [file Data_Sheet_1.PDF]

Supplementary Material

Peptide ligands of AmiA, AliA and AliB proteins determine pneumococcal phenotype

Fauzy Nasher[1, 2], Fernando Aguilar[1], Susanne Aebi[1], Peter W. M. Hermans[4], Manfred Heller[3], Lucy J. Hathaway[1*]

[1]Institute for Infectious Diseases, Faculty of Medicine, [2]Graduate School for Cellular and Biomedical Sciences, University of Bern, Bern, Switzerland, [3]Proteomics and Mass Spectrometry Core Facility, Department of Clinical Research, University of Bern, Bern, Switzerland, [4]Utrecht University Leiden, South Holland Province, Netherlands

*** Correspondence:** Corresponding Author: lucy.hathaway@ifik.unibe.ch

1)

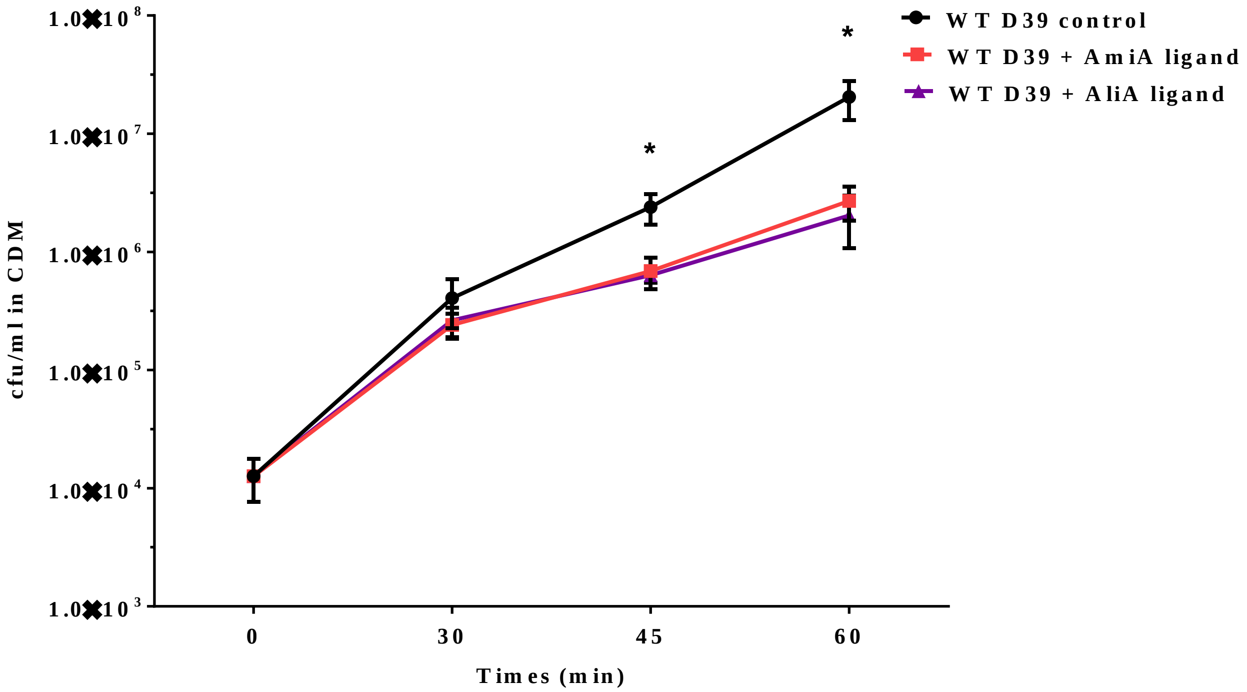

Figure S1 – Effect of AmiA and AliA peptide ligands (0.5mg/mL) on cfu of *Streptococcus pneumoniae* strain D39. Strain D39 was grown in CDM to exponential phase before the addition of the peptide ligands and live bacteria were enumerated at the indicated time points. Results are presented as the mean ± s.d. of three independent experiments, *p<0.05. Reduction of cfu relative to untreated bacteria, but not relative to initial timepoint, indicates that both peptides have a bacteriostatic, rather than bacteriolytic, effect.

2*a*)

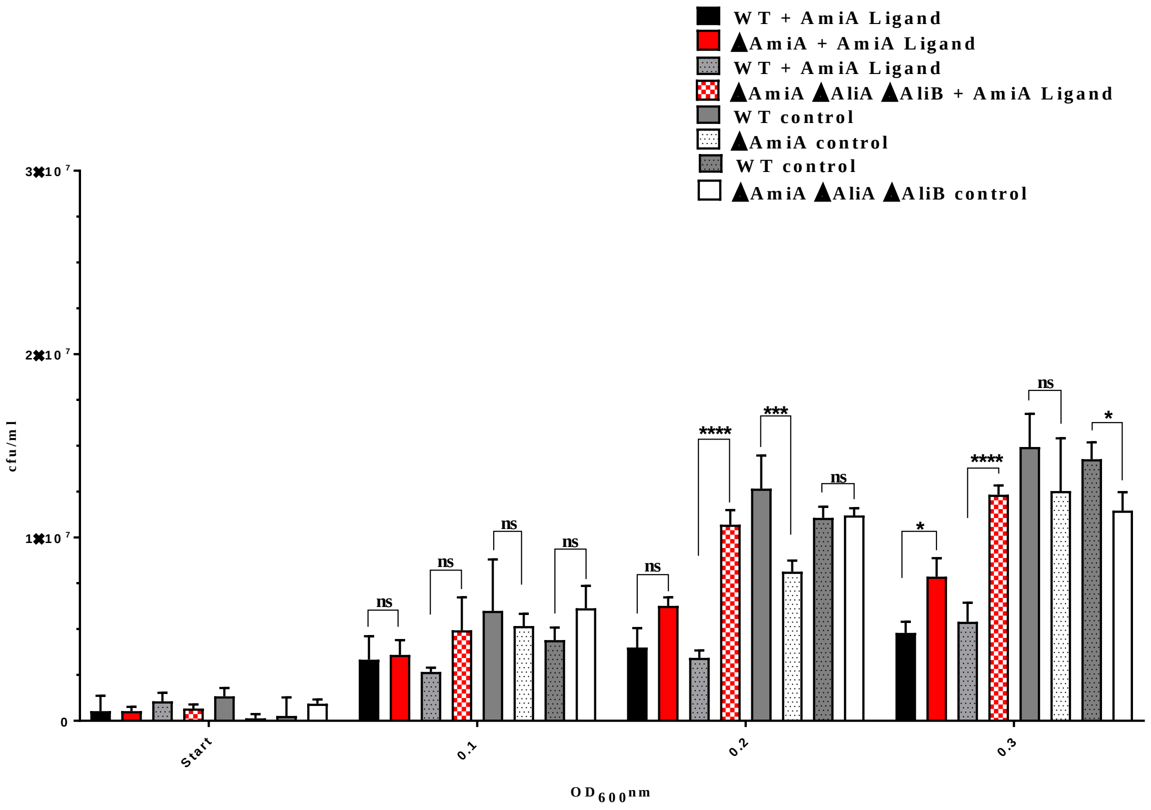
WT + AmiA Ligand
▲AmiA + AmiA Ligand
WT + AmiA Ligand
▲AmiA ▲AliA ▲AliB + AmiA Ligand
WT control
▲AmiA control
WT control
▲AmiA ▲AliA ▲AliB control
3×10^7
2×10^7
1×10^7
0
cfu/ml
ns
****
***
*
Start
0.1
0.2
0.3
OD600nm

***b*)**

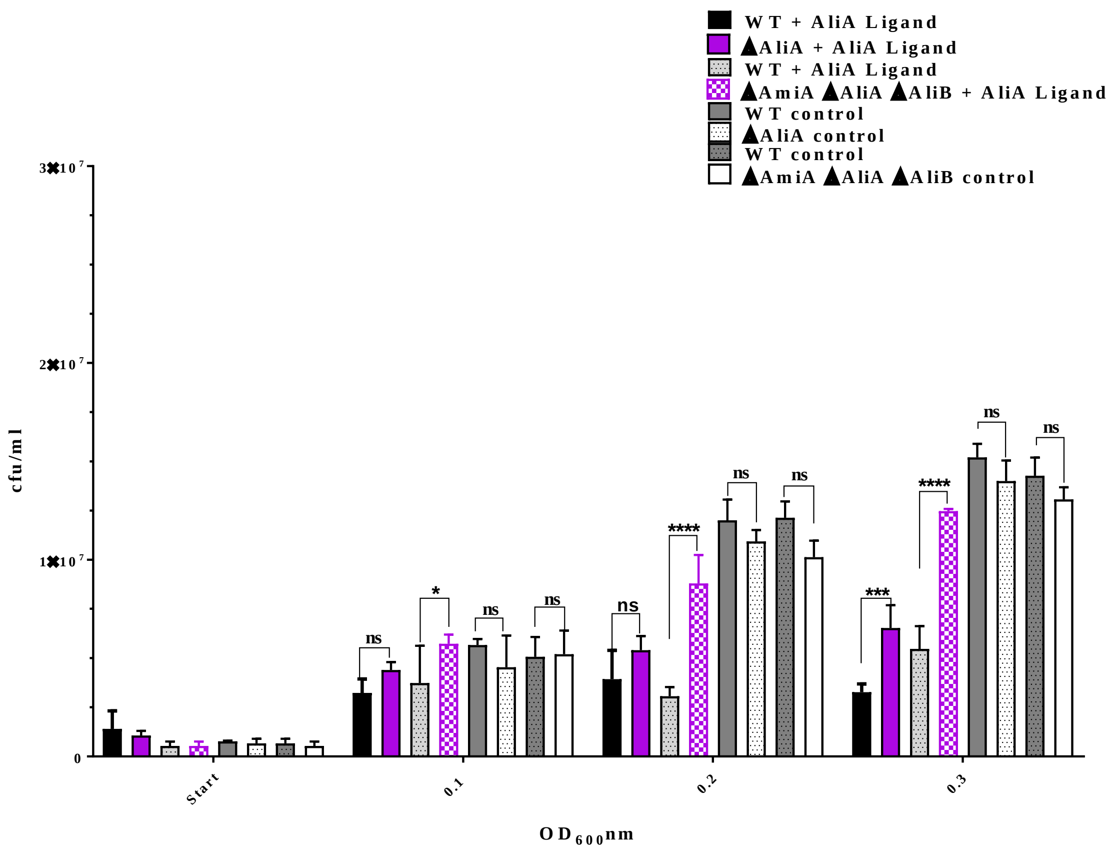
WT + AliA Ligand
▲AliA + AliA Ligand
WT + AliA Ligand
▲AmiA ▲AliA ▲AliB + AliA Ligand
WT control
▲AliA control
WT control
▲AmiA ▲AliA ▲AliB control
cfu/ml
3×10^7
2×10^7
1×10^7
0
Start
0.1
0.2
0.3
OD600nm
ns
*
****
***

***c*)**

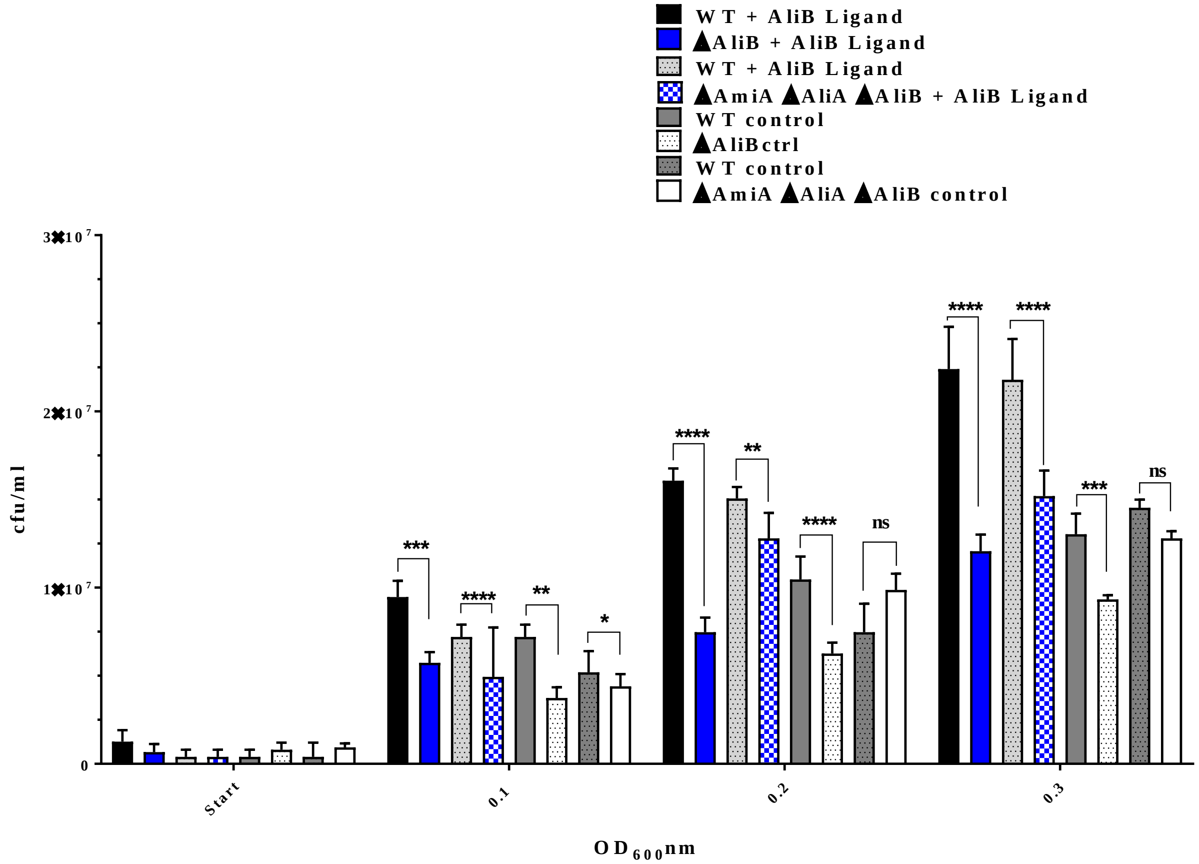

Figure S2 - Competition assay in the presence of peptide ligands (0.5mg/mL). Mixtures of strain D39 and its mutants were grown in CDM and plated at OD_{600nm}=0.1, 0.2, 0.3 on CSBA with and without antibiotics to differentiate between wild type and mutants. The effect of (*a*) AmiA, (*b*) AliA and (*c*) AliB peptide ligand on strain D39 and its mutants. Results are presented as the mean ± s.d. of three independent experiments, **p=0.0021; ***p=0.00021; ****p<0.0001; ns=not significant.

3)

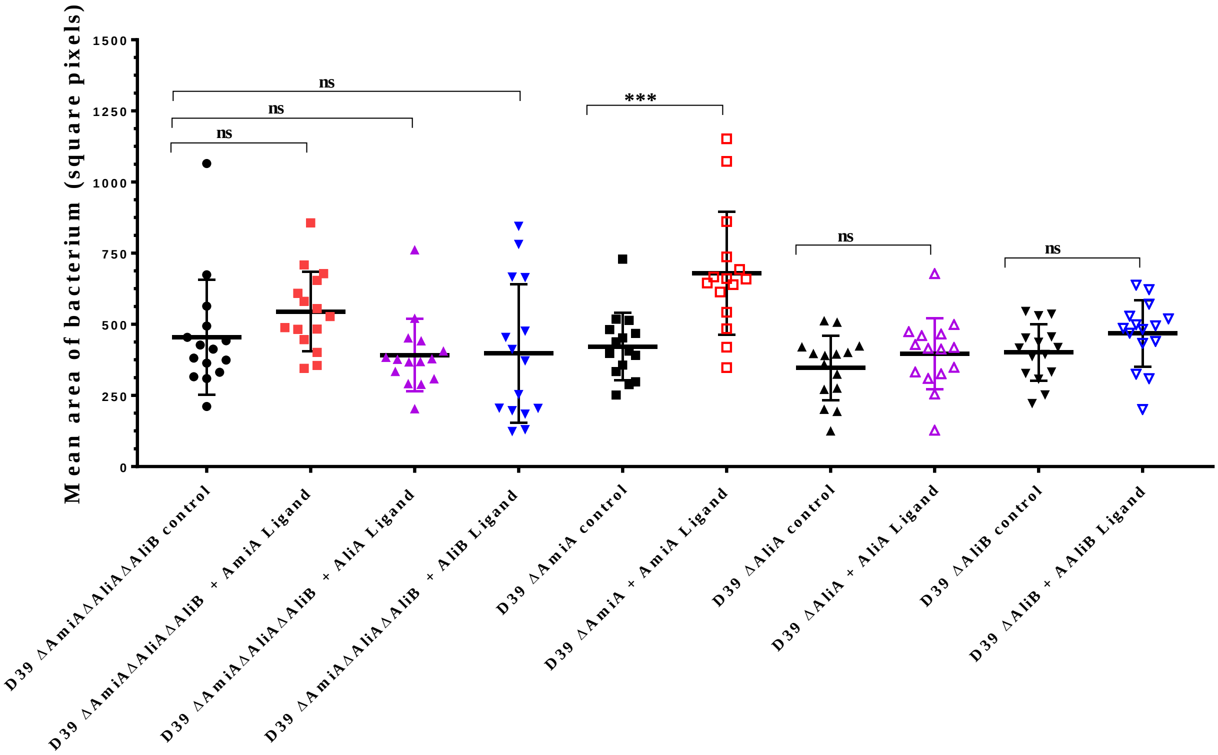

Figure S3 - FITC-dextran exclusion assay. In CDM, bacteria were grown in the presence or absence of AmiA, AliA or AliB peptide ligands (0.5 mg/mL) to OD_{600}=0.2. Capsule thickness of strain D39 ΔAmiA, ΔAliA, ΔAliB and ΔAmiA/ΔAliA/ΔAliB mutants was determined by measuring the zone of exclusion of FITC-dextran. Results are presented as means ± s.d of mean are of bacterium in square pixels, ***p=0.0002; ns=not significant.

4)

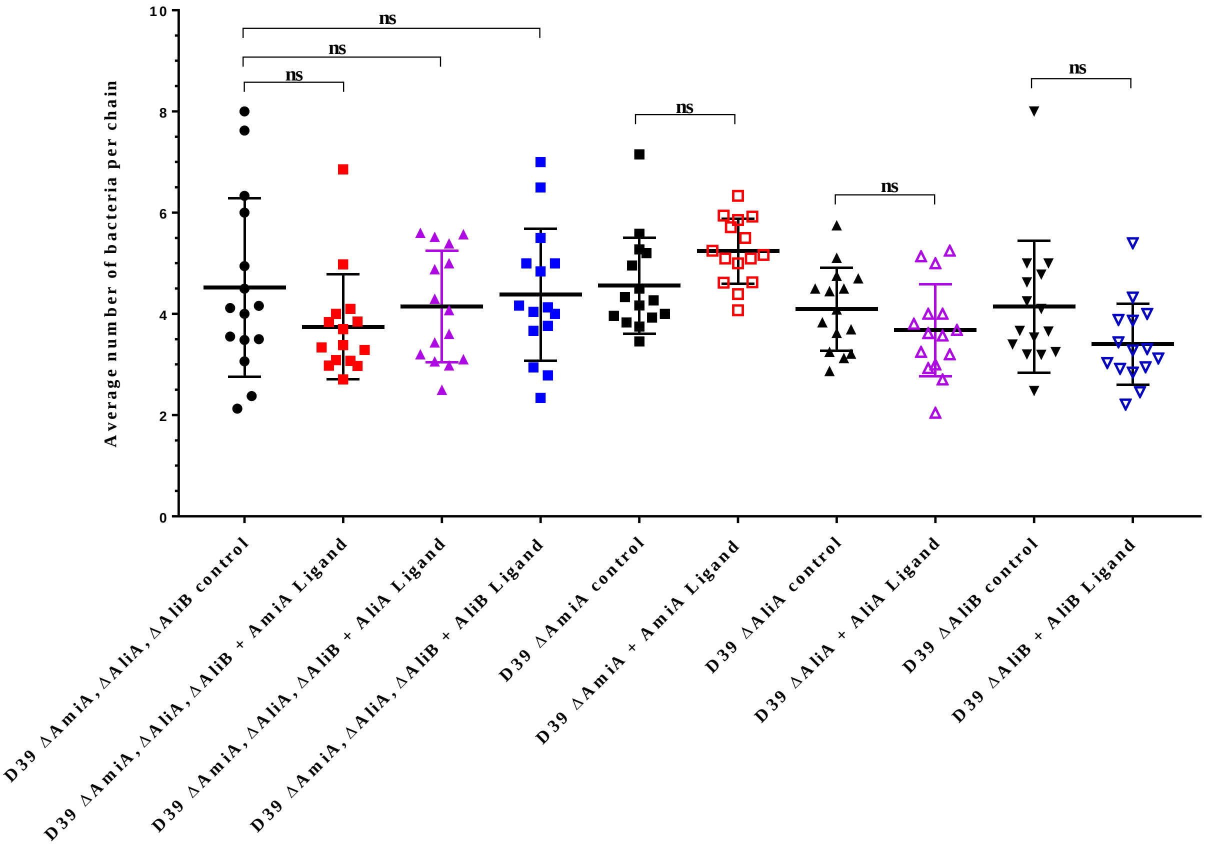

Figure S4 - Analysis of chaining. In CDM, strains D39 ΔAmiA, ΔAliA, ΔAliB and ΔAmiA/ΔAliA/ΔAliB mutants were grown to OD_{600}=0.2 in the presence (0.5mg/mL) and absence of peptide ligands. Results show means ± s.d. of the average number of bacteria per chain, ns=not significant.

5*a*)

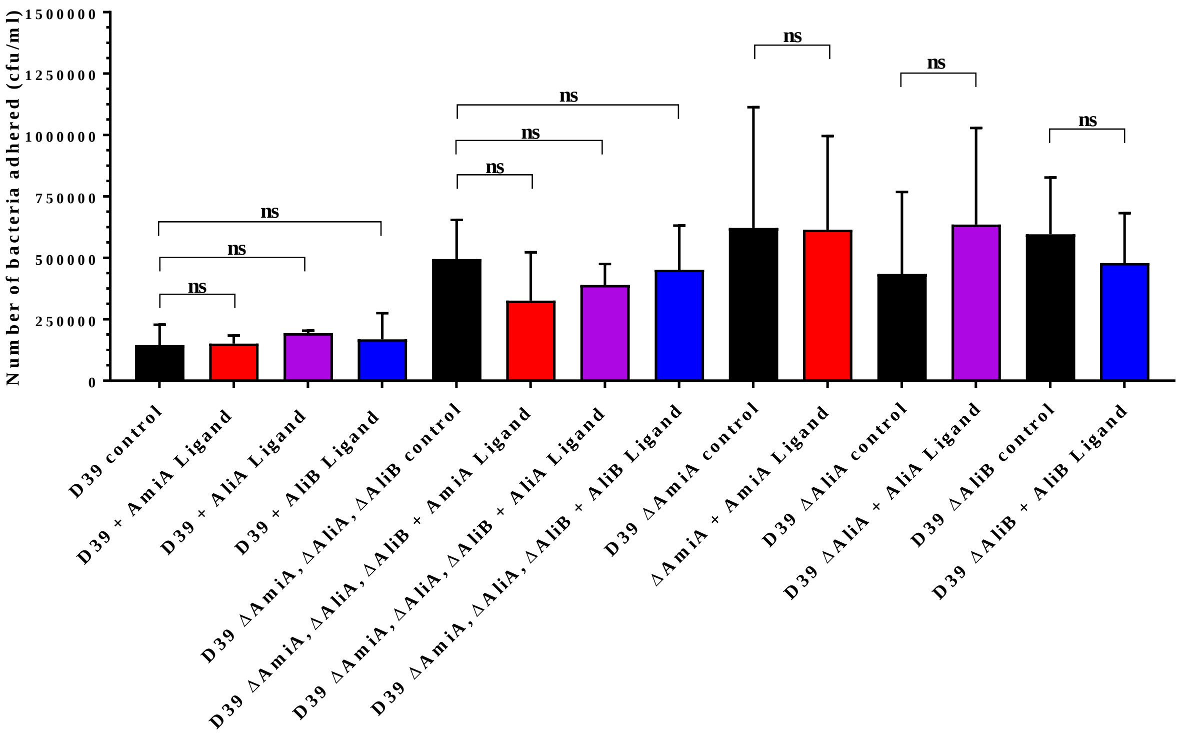
Number of bacteria adhered (cfu/ml)
1500000
1250000
1000000
750000
500000
250000
0
ns
D39 control
D39 + AmiA Ligand
D39 + AliA Ligand
D39 + AliB Ligand
D39 ΔAmiA, ΔAliA, ΔAliB control
D39 ΔAmiA, ΔAliA, ΔAliB + AmiA Ligand
D39 ΔAmiA, ΔAliA, ΔAliB + AliA Ligand
D39 ΔAmiA, ΔAliA, ΔAliB + AliB Ligand
D39 ΔAmiA control
ΔAmiA + AmiA Ligand
D39 ΔAliA control
D39 ΔAliA + AliA Ligand
D39 ΔAliB control
D39 ΔAliB + AliB Ligand

***b*)**

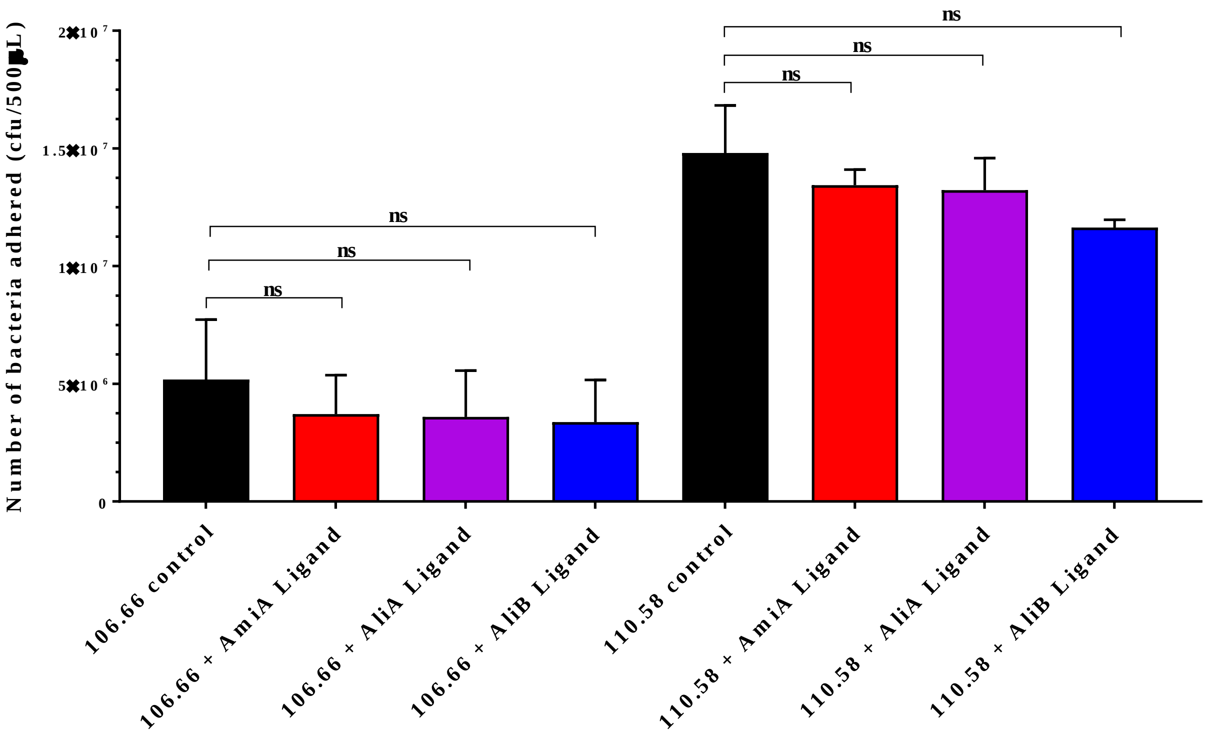

Figure S5 - Adherence to Detroit epithelial cells. In CDM, colony forming units of a) Strain D39 and its mutants, b) strains 106.66 and 110.58 after 1 h incubation at 37 ºC on Detroit 562 nasopharyngeal epithelial cells in the presence (0.5mg/mL) and absence of AmiA, AliA, and AliB peptide ligands. Results are presented as mean ± s.d., ns=not significant.

6)

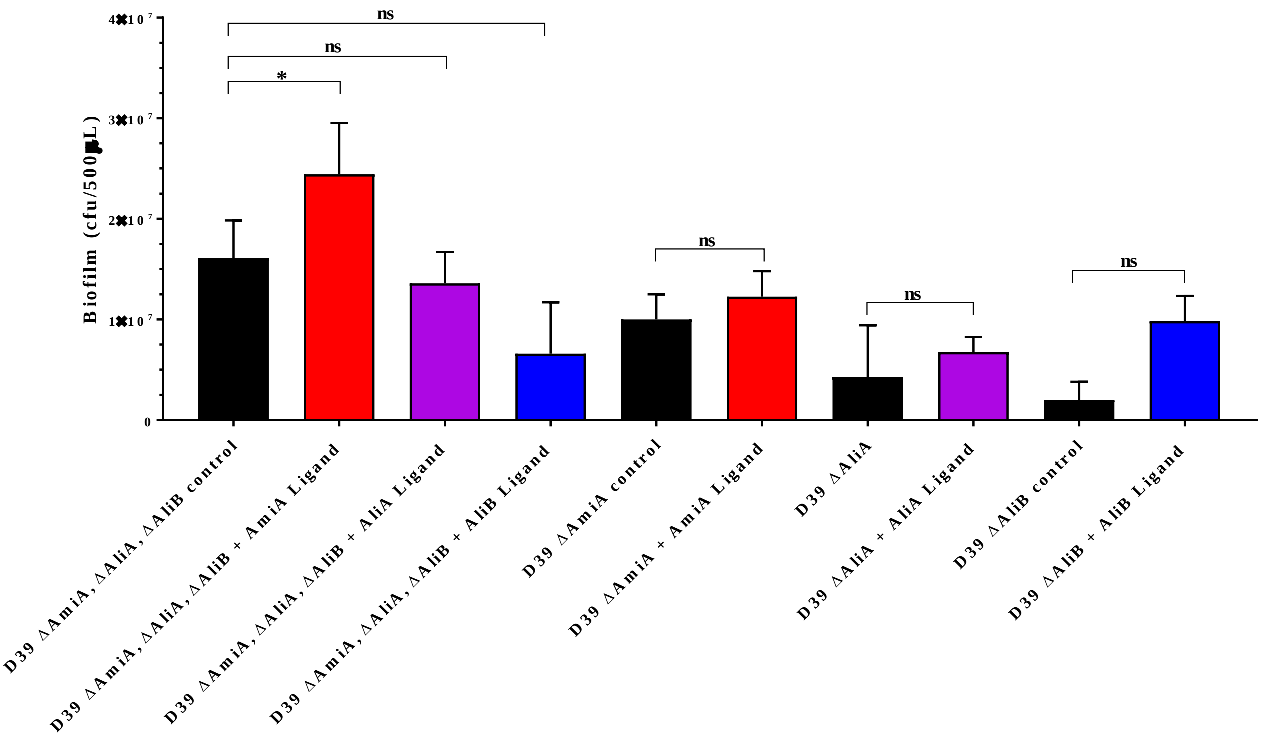

Figure S6 - Quantitative analysis of *in vitro* biofilm formation. CFU counts were determined for D39 ΔAmiA, ΔAliA, ΔAliB and ΔAmiA/ΔAliA/ΔAliB mutants following 16 h of static incubation on Detroit 562 nasopharyngeal epithelial cells with (0.5mg/mL) and without peptide ligands in CDM. Results show means ± s.d., *p=0.0332; ns=not significant.

7a)

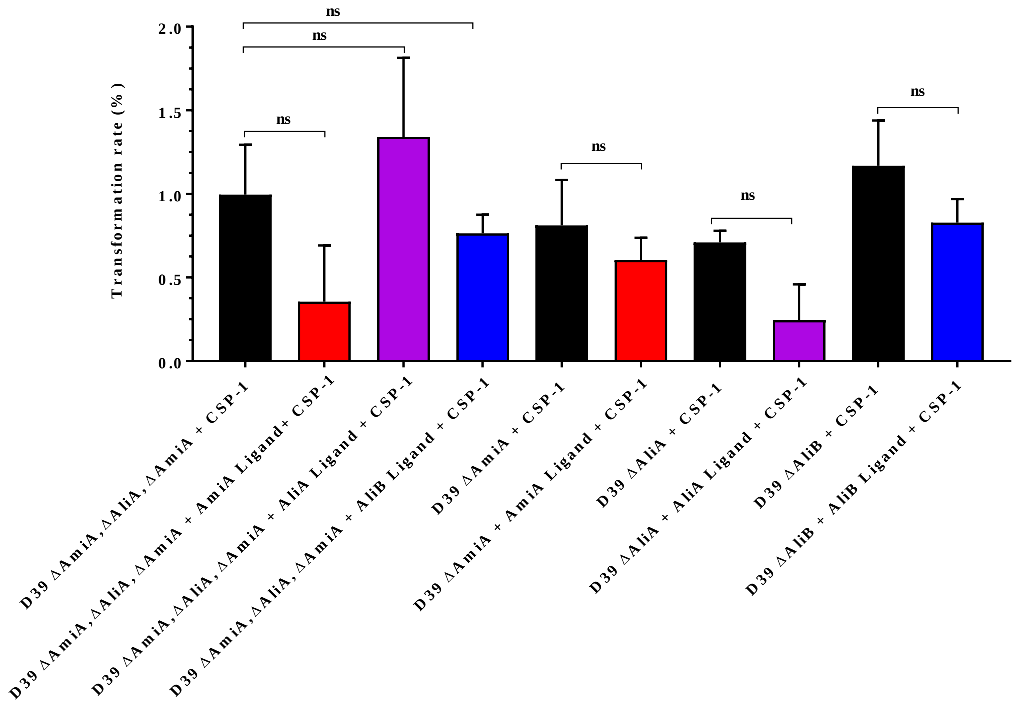
Transformation rate (%)
2.0
1.5
1.0
0.5
0.0
ns
ns
ns
ns
ns
ns
D39 ΔAmiA,ΔAliA,ΔAmiA + CSP-1
D39 ΔAmiA,ΔAliA,ΔAmiA + AmiA Ligand+ CSP-1
D39 ΔAmiA,ΔAliA,ΔAmiA + AliA Ligand + CSP-1
D39 ΔAmiA,ΔAliA,ΔAmiA + AliB Ligand + CSP-1
D39 ΔAmiA + CSP-1
D39 ΔAmiA + AmiA Ligand + CSP-1
D39 ΔAliA + CSP-1
D39 ΔAliA + AliA Ligand + CSP-1
D39 ΔAliB + CSP-1
D39 ΔAliB + AliB Ligand + CSP-1

b)

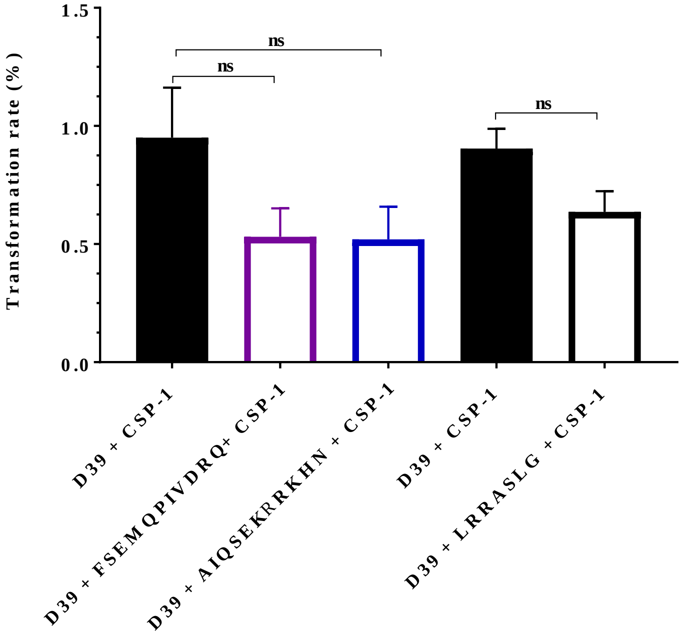
Transformation rate (%)
1.5
1.0
0.5
0.0
ns
ns
ns
D39 + CSP-1
D39 + FSEMQPIVDRQ+ CSP-1
D39 + AIQSEKRRKHN + CSP-1
D39 + CSP-1
D39 + LRRASLG +CSP-1

Figure S7 - Percentage transformation rates. a) D39 ΔAmiA, ΔAliA, ΔAliB and ΔAmiA/ΔAliA/ΔAliB mutants were incubated in CDM with 100ng/mL exogenous CSP in the presence (0.5mg/mL) and absence of AmiA, AliA, and AliB peptide ligands. Results show means ± s.d. of three independent experiments, *p=0.0332; **p=0.0021; ns=not significant. b) Wild type D39 incubated in CDM with 100 ng/mL exogenous CSP in the presence (0.5 mg/mL) and absence of AliA peptide with a single amino acid substitution (FSEMQPIVDRQ), AliB peptide with a single amino acid substitution (AIQSEKRRKHN) and a peptide with an irrelevant sequence (Kemptide) (LRRASLG). There were no significant differences between any of these groups.
