# Supplementary material for: Peptide Ligands of AmiA, AliA, and AliB Proteins Determine Pneumococcal Phenotype
Source: Front Microbiol. 2018 Dec 5;9:3013. doi: 10.3389/fmicb.2018.03013 (PMC6290326; doi:10.3389/fmicb.2018.03013)
Supplement: Supplementary file 2 [file Data_Sheet_2.PDF]

Table S1: Change in protein expression in D39 ΔAmiA, ΔAliA, ΔAliB and ΔAmiAΔAliAΔAliB mutants following exposure to AmiA, AliA and AliB peptide ligands.

Peptide ligand	Strains	Protein ID	Gene name	Description	Difference	q
			**AKTIKTQTR**	**(AmiA peptide ligand)**		
	**ΔAmiA**					
Upregulated		ABJ53661	bglA-1	6-phospho-beta-glucosidase	-21.233	0
		ABJ54169		hydrolase, haloacid dehalogenase-like family protein	-20.942	0
		ABJ55133	recN	DNA repair protein RecN	-20.479	0
		ABJ54214		cytidine and deoxycytidylate deaminase family protein	-20.241	0
		ABJ54032	murB	UDP-N-acetylenolpyruvoylglucosamine reductase	-20.871	0
		ABJ55452	penA	penicillin-binding protein 2B	-20.788	0
		ABJ55113		DegV family protein	-20.901	0
		ABJ54714		conserved hypothetical protein	-1.330	0.0490
Downregulated						
		ABJ54640		conserved hypothetical protein TIGR00043	20.641	0
		ABJ54439	atpH	ATP synthase F1, delta subunit	20.415	0
		ABJ55512		RNA methylase family protein UPF0020, putative	19.868	0
		ABJ54264	clpL	ATP-dependent Clp protease, ATP-binding subunit	20.840	0
		ABJ54376	recG	ATP-dependent DNA helicase RecG	20.875	0
		ABJ53927	parC	DNA topoisomerase IV, A subunit	20.441	0
		ABJ54245		TPR domain protein	21.075	0
		ABJ54281		ABC transporter ATP-binding protein	20.718	0
		ABJ55333	pbpX	penicillin-binding protein 2X	19.904	0
		ABJ53738	mraW	S-adenosyl-methyltransferase MraW	20.249	0
		ABJ54532	livG	branched-chain amino acid ABC transporter, ATP-binding protein	20.912	0
		ABJ55035	mtnN	MTA/SAH nucleosidase	20.479	0
		ABJ54159	manM	PTS system, mannose-specific IIC component	19.996	0
		ABJ54677		alcohol dehydrogenase, zinc-containing	20.807	0
		ABJ54958	vex2	ABC transporter, ATP-binding protein Vexp2	20.224	0
		ABJ55145	malQ	4-alpha-glucanotransferase	21.032	0
		ABJ53626	blpR	response regulator BlpR	21.324	0
		ABJ54844		Cof family protein	21.131	0
		ABJ54991		Cof family protein	19.718	0
		ABJ54242		conserved hypothetical protein	21.111	0
		ABJ54736	estA	tributyrin esterase	21.288	0
		ABJ55280		protein jag (SpoIIIJ-associated protein), putative	2.2784	0.0059
	**ΔAmiA, ΔAliA, ΔAliA**					
Upregulated						
		ABJ53927	parC	DNA topoisomerase IV, A subunit	-20.921	0
		ABJ54331	nth	endonuclease III	-20.921	0
		ABJ54555		conserved hypothetical protein	-20.410	0
		ABJ54958	vex2	ABC transporter, ATP-binding protein Vexp2	-20.110	0
		ABJ54192	gldA	glycerol dehydrogenase	-21.003	0
		ABJ53738	mraW	S-adenosyl-methyltransferase MraW	-21.454	0
		ABJ54245		TPR domain protein	-20.709	0

		ABJ54300	ispA	geranyltranstransferase	-21.353	0
		ABJ54125		conserved hypothetical protein	-21.048	0
		ABJ54729		conserved hypothetical protein	-20.332	0
Downregulated						
		ABJ54331	nth	endonuclease III	20.347	0
		ABJ55265		conserved hypothetical protein	20.194	0
		ABJ54104		L-asparaginase, putative	21.499	0
			**FNEMQPIVDRQ (AliA peptide ligand)**			
	**ΔAliA**					
Upregulated						
		ABJ54104		L-asparaginase, putative	-21.909	0
		ABJ54728	dnaB	replicative DNA helicase	-20.336	0
		ABJ54281		ABC transporter ATP-binding protein	-20.260	0
		ABJ54729		conserved hypothetical protein	-19.793	0
		ABJ54958	vex2	ABC transporter, ATP-binding protein Vexp2	-20.123	0
		ABJ53755	amiE	oligopeptide ABC transporter, ATP-binding protein AmiE	-20.019	0
		ABJ54341		agmatine deiminase	-20.088	0
		ABJ54511	tyrA	prephenate dehydrogenase	-20.907	0
		ABJ55035	mtnN	MTA/SAH nucleosidase	-20.602	0
		ABJ54844		Cof family protein	-19.761	0
Downregulated						
		ABJ54130	nadD	nicotinate (nicotinamide) nucleotide adenylyltransferase	20.671	0
		ABJ55452	penA	penicillin-binding protein 2B	20.446	0
		ABJ55378	cbiO1	cobalt ABC transporter, ATP-binding protein CbiO1	21.846	0
		ABJ54295	pbp2A	penicillin-binding protein 2A	2.816	0
		ABJ55031	scrK	fructokinase	1.956	0.0061
		ABJ55098	fabZ	beta-hydroxyacyl-(acyl-carrier-protein) dehydratase FabZ	1.602	0.0245
		ABJ54514	ftsL	cell division protein FtsL	2.008	0.0477
	**ΔAmiA, ΔAliA, ΔAliA**					
Upregulated						
		ABJ53927	parC	DNA topoisomerase IV, A subunit	-20.441	0
		ABJ54958	vex2	ABC transporter, ATP-binding protein Vexp2	-20.465	0
		ABJ54555		conserved hypothetical protein	-20.637	0
		ABJ54300	ispA	geranyltranstransferase	-21.179	0
		ABJ54192	gldA	glycerol dehydrogenase	-20.775	0
		ABJ54245		TPR domain protein	-20.759	0
		ABJ53738	mraW	S-adenosyl-methyltransferase MraW	-20.293	0
		ABJ54281		ABC transporter ATP-binding protein	-20.362	0
		ABJ54729		conserved hypothetical protein	-20.727	0
Downregulated						
		ABJ54047		ABC transporter, ATP-binding/permease protein	23.416	0
		ABJ55333	pbpX	penicillin-binding protein 2X	20.063	0
		ABJ53755	amiE	oligopeptide ABC transporter, ATP-binding protein AmiE	19.939	0
		ABJ55060	proB	glutamate 5-kinase	20.640	0
		ABJ54532	livG	branched-chain amino acid ABC transporter, ATP-binding protein	20.613	0
		ABJ54728	dnaB	replicative DNA helicase	20.567	0
		ABJ55265		conserved hypothetical protein	20.194	0
		ABJ54566		GTP-binding protein	20.549	0
		ABJ53612		potassium uptake protein, Trk family protein	20.667	0

	ABJ55369		YbbR-like lipoprotein, putative	20.713	0
	ABJ53699		lipoprotein, putative	19.888	0
	ABJ55452	penA	penicillin-binding protein 2B	19.998	0
			**AIQSEKARKHN (AliB peptide ligand)**		
**ΔAliB**					
Upregulated					
	ABJ54640		conserved hypothetical protein TIGR00043	-20.697	0
	ABJ55026		transcriptional regulator, NrdR family protein	-21.469	0
	ABJ54736	estA	tributyrin esterase	-20.679	0
	ABJ55333	pbpX	penicillin-binding protein 2X	-20.609	0
	ABJ54729		conserved hypothetical protein	-20.598	0
	ABJ54511	tyrA	prephenate dehydrogenase	-20.830	0
	ABJ54306		conserved hypothetical protein	-21.119	0
Downregulated					
	ABJ54245		TPR domain protein	20.105	0
	ABJ54044		conserved hypothetical protein	21.490	0
	ABJ55427		amino acid ABC transporter, amino acid-binding protein, putative	20.972	0
	ABJ54192	gldA	glycerol dehydrogenase	20.784	0
	ABJ53793	comE	response regulator	21.284	0
	ABJ54271	licD2	phosphotransferase LicD2	19.924	0
	ABJ54160		conserved hypothetical protein	20.837	0
	ABJ54923		conserved hypothetical protein TIGR01440	20.868	0
	ABJ55122	budA	alpha-acetolactate decarboxylase	20.390	0
	ABJ55060	proB	glutamate 5-kinase	20.322	0
	ABJ55004	relA	GTP pyrophosphokinase	19.754	0
	ABJ54457		amino acid ABC transporter, ATP-binding protein	20.567	0
	ABJ53699		lipoprotein, putative	20.904	0
	ABJ55113		DegV family protein	20.171	0
	ABJ55472		DegV family protein	20.140	0
	ABJ53912		conserved hypothetical protein	21.060	0
	ABJ54125		conserved hypothetical protein	20.486	0
	ABJ54147;ABJ54042;ABJ55306	IS630-Spn1, transposase Orf1;pep chromosome:ASM1436v1:Chromosome:86577:86735:1 gene:SPD_0084		1.570	0.00892308
	ABJ53808	agaS	sugar isomerase domain protein AgaS	3.229	0.00032
	ABJ54969		amino acid ABC transporter, ATP-binding protein	1.839	0.02044444
	ABJ55402		conserved hypothetical protein	1.447	0.04606897
	ABJ55485	cysS	cysteinyl-tRNA synthetase	1.832	0.03828571
**ΔAmiA, ΔAliA, ΔAliA**					
Upregulated					
	ABJ54729		conserved hypothetical protein	-20.173	0

	ABJ53927	parC	DNA topoisomerase IV, A subunit	-20.383	0
Downregulated					
	ABJ53918	trmB	tRNA (guanine-N(7)-)-methyltransferase	20.049	0
	ABJ55333	pbpX	penicillin-binding protein 2X	20.063	0
	ABJ54130	nadD	nicotinate (nicotinamide) nucleotide adenylyltransferase	21.974	0
	ABJ54214		cytidine and deoxycytidylate deaminase family protein	20.413	0
	ABJ54032	murB	UDP-N-acetylenolpyruvoylglucosamine reductase	20.157	0
	ABJ53990		CBS domain protein	20.586	0
	ABJ54271	licD2	phosphotransferase LicD2	19.287	0
	ABJ54628		conserved hypothetical protein	20.356	0
	ABJ54439	atpH	ATP synthase F1, delta subunit	19.956	0
	ABJ55410		oxidoreductase, putative	20.434	0
	ABJ53755	amiE	oligopeptide ABC transporter, ATP-binding protein AmiE	19.939	0
	ABJ54331	nth	endonuclease III	20.347	0
	ABJ54010	pcrA	ATP-dependent DNA helicase PcrA	20.473	0
	ABJ54991		Cof family protein	20.208	0
	ABJ54532	livG	branched-chain amino acid ABC transporter, ATP-binding protein	20.613	0
	ABJ54376	recG	ATP-dependent DNA helicase RecG	19.643	0
	ABJ53793	comE	Response regulator	20.792	0
	ABJ54306		conserved hypothetical protein	20.770	0
	ABJ55265		conserved hypothetical protein	20.194	0
	ABJ54566		GTP-binding protein	20.549	0
	ABJ55133	recN	DNA repair protein RecN	20.271	0
	ABJ53612		potassium uptake protein, Trk family protein	20.667	0
	ABJ53838		S4 domain protein	20.422	0
	ABJ54169		hydrolase, haloacid dehalogenase-like family protein	21.079	0
	ABJ55072		conserved hypothetical protein	21.861	0
	ABJ55031	scrK	fructokinase	1.453	0.0194
	ABJ54104		L-asparaginase, putative	1.623	0.028
	ABJ54124		ABC transporter, ATP-binding protein	1.925	0.0304

Negative values indicate a lower value in the absence of the peptide ligands compared to their presence.
