# Supplementary material for: Peptide Ligands of AmiA, AliA, and AliB Proteins Determine Pneumococcal Phenotype
Source: Front Microbiol. 2018 Dec 5;9:3013. doi: 10.3389/fmicb.2018.03013 (PMC6290326; doi:10.3389/fmicb.2018.03013)
Supplement: Supplementary file 3 [file Data_Sheet_3.PDF]

Supplementary Table S2 - All proteomic data.

Table S2

Majority protein IDs	Gene Name	Description	Q-value	Score	Intensity	MS/MS count	AliA-CTR vs AliA-AliA Significant	AliA-CTR vs AliA-AliA p-value(-Log)	AliA-CTR vs AliA-AliA q-value	AliA-CTR vs AliA-AliA Δ	AliB-CTR vs AliB-AliB Significant	AliB-CTR vs AliB-AliB p-value(-Log)	AliB-CTR vs AliB-AliB q-value	AliB-CTR vs AliB-AliB Δ	AmiA-CTR vs AmiA-AmiA Significant	AmiA-CTR vs AmiA-AmiA p-value(-Log)	AmiA-CTR vs AmiA-AmiA q-value	AmiA-CTR vs AmiA-AmiA Δ	AliA-AliA. median LFQ int	AliA-CTR. median LFQ int	AliB-AliB. median LFQ int	AliB-CTR. median LFQ int	AmiA-AmiA. median LFQ int	AmiA-CTR. median LFQ int	TM-CTR vs TM-AliA Significant
ABJ53606	pyrH	uridylate kinase	0	171.12	939840000	201		0.498450812	0.531067568	-0.388731003		0.290649217	0.721553398	-0.376343091		0.94352589	0.411956989	0.556693077	24.6649	23.9972	23.616	23.7912	24.2644	25.3412	
ABJ53608	gidA	tRNA uridine 5-carboxymethylaminomethyl modification enzyme gidA(glucose-inhibi	0	10.641	160030000	38		0.160774081	0.817781753	-0.094418208		0.47028593	0.606074074	-0.596420606		0.800837873	0.514915254	0.364945094	22.1816	22.1352	21.7945	21.926	21.5009	22.0865	
ABJ53611		conserved hypothetical protein	0	70.2	1969700000	563		1.205282776	0.406556213	0.477016767		0.142476672	0.88544086	-0.066328367		0.743832097	0.530409938	-0.338487625	25.7866	26.1853	26.0354	25.9389	25.6774	25.4164	
ABJ53612		potassium uptake protein, Trk family protein	0.0014749	3.6578	13906000	10		0	0.998552822	0		0	0.998554913	0		0	0.99856528	0	0	0	0	0	0	0	1
ABJ53617		GTP1/Obg family GTP-binding protein	0	110.38	651080000	172		2.073430423	0.379925234	-0.514455795		0.742134438	0.647615721	0.35218811		0.687694305	0.440138614	0.605696042	24.0217	23.6238	23.2283	23.5825	23.6946	23.8349	
ABJ53624	trpA	tryptophan synthase, alpha subunit	0.0081855	1.6972	86375000	47		0.204526207	0.660414747	-0.309197426		0.107622185	0.899542088	0.048537572		1.038470492	0.444917073	-0.479167938	21.1457	21.0519	21.1173	21.3039	21.4314	20.8717	
ABJ53626	blpR	response regulator BlpR	0	4.6447	119470000	32		1.094645203	0.339	1.090683619		0.412115945	0.638953271	-0.549230258	1	12.47381916	0	21.32498487	20.2139	21.8431	20.5054	19.5023	0	21.3047	
ABJ53627		conserved hypothetical protein	0	107.57	104230000	77		0.22421972	0.743352459	0.165117264		0.762802231	0.445553719	0.734136581		1.205576045	0.387726496	0.657776515	21.384	21.4128	21.0717	21.5442	20.8869	21.3469	
ABJ53631	rplI	ribosomal protein L9	0	193.48	3077700000	438		0.69445965	0.40558209	-0.742117564		0.255311524	0.756726368	-0.227631251		1.074778442	0.343692308	0.84405899	26.2779	26.2893	25.7385	25.5213	26.1696	26.5059	
ABJ53634	thrS	threonyl-tRNA synthetase	0	323.31	2368700000	594		0.474691261	0.662100917	-0.189723333		0.043531334	0.904632953	-0.06159242		0.685451134	0.489585185	0.43847243	25.5322	25.2324	24.8441	25.4278	25.0906	25.3709	
ABJ53635		oligopeptide ABC transporter,oligopeptide-binding protein	0	12.359	163740000	36		0.986745583	0.379542857	-0.700282097		1.427017935	0.22	0.994447072		1.699515655	0.273055556	0.779541016	22.083	21.7017	21.2584	22.2177	22.054	22.5935	
ABJ53641		acetyltransferase, GNAT family protein	0	7.4783	78910000	20		1.200012943	0.389454545	0.710841497		0.214140971	0.74405291	0.313093185		0.285952257	0.599126316	0.414587339	20.1388	20.9469	20.8331	20.7224	20.6109	21.2294	
ABJ53642		conserved hypothetical protein	0	11.513	248870000	48		0.299199734	0.549690852	0.528039932		0.013822269	0.951735338	-0.028709412		0.004023453	0.986206096	0.009731611	21.5639	22.2034	22.4468	22.0789	22.2369	22.4753	
ABJ53643		iron-compound ABC transporter, iron-compound-binding protein	0	4.9822	928760000	15		0.390892639	0.502054054	0.585506439		0.07303097	0.867216418	-0.14493656		0.336292437	0.605697917	-0.346383413	24.9261	24.867	24.8992	24.6299	24.8227	24.8702	
ABJ53651	amiA	oligopeptide ABC transporter, oligopeptide-binding protein AmiA	0	323.31	11991000000	2114		0.794489578	0.397272727	-0.772727013		0.054774904	0.896946459	-0.087058385		0.81146295	0.445618605	-0.518758456	28.9304	27.9422	28.0204	28.6151	22.5126	21.8446	
ABJ53652	tuf	translation elongation factor Tu	0	323.31	2.58E+11	8861	[illegible]	[illegible]	[illegible]	[illegible]	[illegible]	[illegible]	[illegible]	[illegible]	[illegible]	[illegible]	[illegible]	[illegible]	[illegible]	[illegible]	[illegible]	[illegible]	[illegible]	[illegible]	[illegible]
ABJ53654	upp	uracil phosphoribosyltransferase	0	162.05	2300400000	487	[illegible]	[illegible]	[illegible]	[illegible]	[illegible]	[illegible]	[illegible]	[illegible]	[illegible]	[illegible]	[illegible]	[illegible]	[illegible]	[illegible]	[illegible]	[illegible]	[illegible]	[illegible]	[illegible]
ABJ53655	cbpA	choline binding protein A	0	168.84	805350000	328	[illegible]	[illegible]	[illegible]	[illegible]	[illegible]	[illegible]	[illegible]	[illegible]	[illegible]	[illegible]	[illegible]	[illegible]	[illegible]	[illegible]	[illegible]	[illegible]	[illegible]	[illegible]	[illegible]
ABJ53656		conserved hypothetical protein	0	323.31	2745200000	393	[illegible]	[illegible]	[illegible]	[illegible]	[illegible]	[illegible]	[illegible]	[illegible]	[illegible]	[illegible]	[illegible]	[illegible]	[illegible]	[illegible]	[illegible]	[illegible]	[illegible]	[illegible]	[illegible]
ABJ53658	pulA	pullulanase, type I	0	173.29	1057400000	549	[illegible]	[illegible]	[illegible]	[illegible]	[illegible]	[illegible]	[illegible]	[illegible]	[illegible]	[illegible]	[illegible]	[illegible]	[illegible]	[illegible]	[illegible]	[illegible]	[illegible]	[illegible]	[illegible]
[illegible]	[illegible]	[illegible]	[illegible]	[illegible]	[illegible]	[illegible]	[illegible]	[illegible]	[illegible]	[illegible]	[illegible]	[illegible]	[illegible]	[illegible]	[illegible]	[illegible]	[illegible]	[illegible]	[illegible]	[illegible]	[illegible]	[illegible]	[illegible]	[illegible]	[illegible]

ABJ54204		general stress protein 24, putative	0	323.31	5359200000	1033		1.487207854	0.647315136	0.174530029		0.892774297	0.707112676	0.253188769		0.869449319	0.611815385	-0.227977117	26.3271	26.4483	27.5184	27.6885	27.0861	26.8309	
ABJ54208	rfbD	dTDP-4-dehydrorhamnose reductase	0	323.31	7214400000	1165		0.90535237	0.426127168	-0.516651471		0.575265393	0.736114613	0.214335124		1.033257669	0.239457627	1.252360026	27.4729	27.0996	26.6866	27.1817	27.1244	27.254	
ABJ54209		conserved hypothetical protein	0.0014793	3.7652	122500000	39		0.272465799	0.6279375	-0.335808754		1.06815083	0.447779817	0.627361298		0.813068786	0.412531646	0.671690941	21.5796	21.4197	20.9917	21.7167	21.1256	21.8262	
ABJ54214		cytidine and deoxycytidylate deaminase family protein	0	14.676	33243000	16		0.656794741	0.452121739	0.483612696		0.664180248	0.60681407	0.443605105	-1	13.29902744	0	-20.24159304	20.3748	21.0035	20.6776	20.8142	20.4566	0	
ABJ54224	accC	acetyl-CoA carboxylase, biotin carboxylase	0	75.398	1258800000	446		1.561845835	0.406657343	-0.475208282		0.014637904	0.980965926	-0.007729212		0.064926614	0.888508527	0.043547948	24.9158	24.5731	24.4538	24.4853	24.9505	24.6813	
ABJ54226	dut	deoxyuridine 5-triphosphate nucleotidohydrolase	0	33.296	358850000	185		0.785966146	0.410068027	0.632860184		0.079138936	0.889420959	-0.082212448		1.464701739	0.204780488	-1.120891571	23.0757	23.9732	23.8851	23.4294	23.8495	22.348	
ABJ54228		phosphatase, putative	0	55.826	1960900000	301		0.329311235	0.793046729	-0.096569061		0.694066103	0.801099366	-0.109139125		0.217423941	0.832574194	-0.056147893	25.7016	25.4451	25.9276	25.7808	25.4372	25.388	
ABJ54229	divIVA	cell division protein DivIVA	0	323.31	1455400000	395		0.164784169	0.816064516	0.094803492		0.193542855	0.825032787	0.128427823		1.236831794	0.389661972	-0.586655935	24.7086	24.7093	24.9291	25.3897	25.1366	24.7904	
ABJ54232	rnr	ribonuclease R	0	31.081	112030000	63		0.103703637	0.828474336	0.107632955		0.000719846	0.999384615	0.001077652		0.073571239	0.832066445	0.104880651	21.0499	20.8928	20.993	21.0675	21.3639	21.7598	
ABJ54233		cation-transporting ATPase, E1-E2 family protein	0	13.148	376690000	53		1.069933775	0.316606061	1.265493393		0.831185467	0.438017391	0.718180339		0.35497723	0.579054945	0.390289942	22.781	23.4289	22.7039	23.1849	23.1138	23.1959	
ABJ54234		exonuclease	0.0028694	2.7439	196980000	8		0.07719056	0.838170435	0.11760203		0.323648507	0.731535168	0.306784312		0.151164046	0.771756522	-0.118553797	22.6477	22.5198	22.9881	22.7683	22.6219	22.3527	
ABJ54238	ileS	isoleucyl-tRNA synthetase	0	323.31	4614600000	1050		1.144969005	0.539605263	-0.278106689		1.006343155	0.678496124	0.278564771		0.732476831	0.409710983	0.682786942	26.7992	26.5338	26.4787	26.8574	26.7833	26.9817	
ABJ54239		conserved hypothetical protein	0	28.855	200310000	70		0.001496495	0.995715116	0.00243632		0.051167359	0.890851064	-0.129550934		0.157301292	0.686495935	0.30800279	21.1568	21.2044	21.0402	20.1035	21.4549	22.2438	
ABJ54241	folE	GTP cyclohydrolase I	0	31.886	428690000	145		1.556240778	0.398519685	-0.50790151		0.155910449	0.89491358	0.057368279		0.048777769	0.88828483	0.050772667	23.5833	22.9587	22.9413	23.0109	22.6702	22.6766	
ABJ54242		conserved hypothetical protein	0	5.2874	139690000	83		1.828668509	0.324342857	-0.841309865		0.338312466	0.70785348	-0.414731344	1	11.0435741	0	21.11110051	21.5461	20.4329	21.2362	21.3718	0	20.6166	
ABJ54245		TPR domain protein	0.0029283	3.4006	27569000	16		0	0.985714286	0	1	18.15038518	0	20.10527833	1	13.5053066	0	21.07527574	0	0	0	20.066	0	20.7969	-1
ABJ54247		transcriptional regulator, GntR family protein	0	221.48	3815300000	784		0.502915058	0.609235772	-0.259282748		0.551211938	0.726543046	0.264900843		0.547530275	0.564251429	0.329479853	26.2301	26.1954	26.1391	26.2213	26.0523	26.2337	
ABJ54254	malP	maltodextrin phosphorylase	0	28.588	308810000	67		1.322184933	0.37619802	-0.616666158		2.155979026	0.050533333	1.327020009		0.287967706	0.633326733	0.332286517	22.314	22.0115	20.7068	22.2813	21.7523	21.9692	
ABJ54259	proS	prolyl-tRNA synthetase	0	323.31	6375300000	1496		1.029345644	0.385829787	-0.714687347		0.711104306	0.663242798	0.332996051		0.596820788	0.463577093	0.583040237	27.5611	26.9726	26.6095	26.7144	27.236	27.2455	
ABJ54261	guaB	inosine-5-monophosphate dehydrogenase	0	323.31	7434800000	1656		0.623172697	0.662805556	-0.178877831		0.320779547	0.822174274	-0.112938245		0.524459289	0.731777778	0.113573392	27.2378	27.021	27.543	27.2936	27.135	27.1513	
ABJ54264	clpL	ATP-dependent Clp protease, ATP-binding subunit	0	31.056	43376000	16		0.157155599	0.766867188	-0.163288752		0	0.988555079	0	1	14.01118395	0	20.84001446	20.6183	20.38	0	0	0	20.753	
ABJ54265	hslO	chaperonin, 33 kDa	0	93.147	867430000	280		0.182855169	0.881778512	-0.046240807		0.539871716	0.666064516	-0.366752942		0.471888654	0.651381166	0.207381884	24.1837	24.1483	23.8319	23.76	23.4602	23.8008	
ABJ54268		ABC transporter, ATP-binding protein	0	131.76	1347800000	390		0.842759881	0.506015267	-0.357532501		0.108829192	0.901117547	0.051227252		0.080184921	0.913789313	0.028500239	24.8046	24.5789	24.5674	24.7617	25.121	25.1906	
ABJ54269		ABC transporter, substrate binding lipoprotein	0	12.713	81798000	17		0.108177756	0.770307992	-0.233339945		1.246878451	[illegible]	[illegible]	[illegible]	[illegible]	[illegible]	[illegible]	[illegible]	[illegible]	[illegible]	[illegible]	[illegible]	[illegible]	[illegible]
ABJ54271	licD2	phosphotransferase LicD2	0	50.649	18507000	23		0.316089553	0.608313514	0.33283329	[illegible]	[illegible]	[illegible]	[illegible]	[illegible]	[illegible]	[illegible]	[illegible]	[illegible]	[illegible]	[illegible]	[illegible]	[illegible]	[illegible]	[illegible]
ABJ54278	aliA	oligopeptide ABC transporter, oligopeptide-binding protein AliA	0	323.31	8091500000	1534		0.00700974	0.972095097	0.031663895	[illegible]	[illegible]	[illegible]	[illegible]	[illegible]	[illegible]	[illegible]	[illegible]	[illegible]	[illegible]	[illegible]	[illegible]	[illegible]	[illegible]	[illegible]
ABJ54281		ABC transporter ATP-binding protein	0	7.3351	16181000	10	-1	14.87708581	0	-20.26070627	[illegible]	[illegible]	[illegible]	[illegible]	[illegible]	[illegible]	[illegible]	[illegible]	[illegible]	[illegible]	[illegible]	[illegible]	[illegible]	[illegible]	[illegible]
ABJ54293	pfkA	6-phosphofructokinase	0	323.31	4539700000	854		2.807082747	0.538983498	-0.243142764	[illegible]	[illegible]	[illegible]	[illegible]	[illegible]	[illegible]	[illegible]	[illegible]	[illegible]	[illegible]	[illegible]	[illegible]	[illegible]	[illegible]	[illegible]
ABJ54295	pbp2A	penicillin-binding protein 2A	0.0014684	3.5599	281890000	13	1	5.158369053	0	2.816181183	[illegible]	[illegible]	[illegible]	[illegible]	[illegible]	[illegible]	[illegible]	[illegible]	[illegible]	[illegible]	[illegible]	[illegible]	[illegible]	[illegible]	[illegible]
ABJ54296	fabH	3-oxoacyl-(acyl-carrier-protein) synthase III	0	73.305	167480000	57		0.043884363	0.920886762	0.045642217	[illegible]	[illegible]	[illegible]	[illegible]	[illegible]	[illegible]	[illegible]	[illegible]	[illegible]	[illegible]	[illegible]	[illegible]	[illegible]	[illegible]	[illegible]
ABJ54298	ilvE	branched-chain amino acid aminotransferase	0	276.31	4225100000	831		0.312380557	0.767499022	0.120385488	[illegible]	[illegible]	[illegible]	[illegible]	[illegible]	[illegible]	[illegible]	[illegible]	[illegible]	[illegible]	[illegible]	[illegible]	[illegible]	[illegible]	[illegible]
[illegible]	[illegible]	[illegible]	[illegible]	[illegible]	[illegible]	[illegible]	[illegible]	[illegible]	[illegible]	[illegible]	[illegible]	[illegible]	[illegible]	[illegible]	[illegible]	[illegible]	[illegible]	[illegible]	[illegible]	[illegible]	[illegible]	[illegible]	[illegible]	[illegible]	[illegible]

ABJ54863		elongation factor Tu family protein	0	255.92	3549700000
ABJ54877	rpsD	ribosomal protein S4	0	323.31	17213000000
ABJ54880		oligoendopeptidase F, putative	0	156.46	1914900000
ABJ54881	pgk	phosphoglycerate kinase	0	323.31	84464000000
ABJ54884	infB	initiation factor IF-2	0	323.31	8937900000
ABJ54886		secreted 45 kDa protein precursor	0	107.28	493980000
ABJ54887		conserved hypothetical protein	0	77.166	1399100000
ABJ54890	cbf1	cmp-binding-factor 1	0	86.682	1130400000
ABJ54899		amino acid ABC transporter, amino acid-binding protein	0	42.387	873070000
ABJ54901		hydrolase, TatD family protein	0	40.084	588740000
ABJ54905	prsA	ribose-phosphate pyrophosphokinase	0	57.809	375110000
ABJ54906	pgi	glucose-6-phosphate isomerase	0	323.31	11738000000
ABJ54907		conserved hypothetical protein TIGR00103	0	22.381	518230000
ABJ54909		glycosyl hydrolase, family protein 31	0.0028736	2.7524	380460000
ABJ54911	topA	DNA topoisomerase I	0	40.748	1144400000
ABJ54915		enoyl-CoA hydratase/isomerase family protein	0	8.6859	284870000
ABJ54917	rplL	ribosomal protein L7/L12	0	323.31	51624000000
ABJ54918	trpB	tryptophan synthase, beta subunit	0	6.6661	75350000
ABJ54919	purB	adenylosuccinate lyase	0	120.65	1186300000
ABJ54921	ftsX	cell division ABC transporter, permease protein FtsX	0	41.907	146960000
ABJ54923		conserved hypothetical protein TIGR01440	0	4.428	39071000
ABJ54930		hydrolase, haloacid dehalogenase-like family protein	0	25.736	308060000
ABJ54931	adcR	adc operon repressor AdcR	0	4.1882	315120000
ABJ54932	groL	chaperonin GroEL	0	323.31	11642000000
ABJ54933	glyQ	glycyl-tRNA synthetase, alpha subunit	0	29.523	545880000
ABJ54934	rpoD	RNA polymerase sigma-70 factor	0	323.31	1193000000
ABJ54938	gid	tRNA uridine 5-carboxymethylaminomethyl modification enzyme gid	0	168.05	794310000
ABJ54941		conserved hypothetical protein	0	7.2926	55753000
ABJ54943	prmA	ribosomal protein L11 methyltransferase	0	9.9399	92125000
ABJ54944		2,3,4,5-tetrahydropyridine-2-carboxylate N-succinyltransferase, putative	0	138.07	3486000000
ABJ54945		chorismate mutase	0	85.983	896870000
ABJ54946	rplJ	ribosomal protein L10	0	323.31	13944000000
ABJ54948	pepS	aminopeptidase PepS	0	323.31	5908400000
ABJ54949	folP	dihydropteroate synthase	0	7.528	119060000
ABJ54950		DNA polymerase I	0	12.608	158910000
ABJ54953	bta	bacterocin transport accessory protein	0	323.31	22319000000
ABJ54954	gmk	guanylate kinase	0	42.391	640930000
ABJ54955	thiI	thiamine biosynthesis/tRNA modification protein ThiI	0.0028571	2.614	196340000
ABJ54957		phosphoglycerate mutase	0	323.31	92608000000
ABJ54958	vex2	ABC transporter, ATP-binding protein Vexp2	0	6.6994	13977000
ABJ54959	rpsJ	ribosomal protein S10	0	298.93	15533000000
ABJ54962		DNA-directed RNA polymerase, delta subunit, putative	0	147.76	821920000
ABJ54965	nox	NADH oxidase	0	323.31	19224000000
ABJ54967	proWX	choline transporter (glycine betaine transport system permease protein)	0	16.583	551870000
ABJ54969		amino acid ABC transporter, ATP-binding protein	0	36.199	492020000
ABJ54971		conserved hypothetical protein	0	67.817	1042900000
ABJ54972	gyrA	DNA gyrase, A subunit	0	21.121	604700000
ABJ54973		hypersensitive-induced reaction protein 4	0	204.12	2258500000
ABJ54977	hpt	hypoxanthine phosphoribosyltransferase	0	323.31	1919100000
ABJ54983	pyrDa	dihydroorotate dehydrogenase A	0	323.31	9906700000
ABJ54984		conserved hypothetical protein	0	111.67	345550000
ABJ54989	cps2G	glycosyl transferase, group 1 family protein	0	25.281	332990000
ABJ54991		Cof family protein	0.0029112	3.0876	9436800
ABJ54994		serine protease	0	124.84	368130000
ABJ54995	rnz	ribonuclease Z	0	73.326	258340000
ABJ54999		hydrolase, haloacid dehalogenase-like family protein	0	59.467	969180000
ABJ55004	relA	GTP pyrophosphokinase	0.0082192	1.7579	40354000
ABJ55005		amino acid ABC transporter, ATP-binding protein	0	84.936	683020000
ABJ55009	ptsH	phosphocarrier protein HPr	0	323.31	40987000000
ABJ55012	gor	glutathione-disulfide reductase	0	248.23	3617000000
ABJ55021	rplC	ribosomal protein L3	0	323.31	20607000000
ABJ55026		transcriptional regulator, NrdR family protein	0	102.08	100880000
ABJ55028		LysM domain protein	0	323.31	3559800000
ABJ55031	scrK	fructokinase	0	31.006	184520000
ABJ55032	rpmD	ribosomal protein L30	0	193	3337800000
ABJ55035	mtnN	MTA/SAH nucleosidase	0	10.533	124670000
ABJ55036	rpsF	ribosomal protein S6	0	323.31	22752000000
ABJ55037		acetoin dehydrogenase, E1 component, beta subunit, putative	0	84.648	889230000
ABJ55039	cps2I	glycosyl transferase, group 1 family protein, putative	0	5.6712	663680000
ABJ55042		iron-dependent transcriptional regulator	0.005618	2.317	24845000
ABJ55043	rplU	ribosomal protein L21	0	62.535	1489100000
ABJ55046	rpsR	ribosomal protein S18	0	192.27	11441000000
ABJ55047		lipoate-protein ligase, putative	0	73.297	1198500000
ABJ55048	fusA	translation elongation factor G	0	323.31	51332000000
ABJ55051	tig	trigger factor	0	323.31	10998000000
ABJ55053	trmU	tRNA (5-methylaminomethyl-2-thiouridylate)-methyltransferase	0	44.959	538760000
ABJ55054		conserved hypothetical protein	0	17.114	158050000
ABJ55057		Uncharacterized ACR	0	120.98	596170000
ABJ55058	era	GTP-binding protein Era	0	17.135	112290000
ABJ55059	thrC	threonine synthase	0	249.35	2706800000
ABJ55060	proB	glutamate 5-kinase	0.0094851	1.5525	37056000
ABJ55062	lys1	saccharopine dehydrogenase	0	306.55	2453100000
ABJ55064	eno	phosphopyruvate hydratase	0	323.31	2.55E+11
ABJ55069	cps2E	undecaprenylphosphate glucosephosphotransferase Cps2E	0	7.2799	101470000
ABJ55070		ABC transporter, ATP-binding protein	0	107.06	108550000
ABJ55072		conserved hypothetical protein	0.0082079	1.7081	22870000
ABJ55073	lytA	autolysin/N-acetylmuramoyl-L-alanine amidase	0	48.166	3278100000
ABJ55076	trx	thioredoxin	0	323.31	24984000000
ABJ55085		phosphodiesterase, MJ0936 family protein	0	52.438	1953400000
ABJ55088		transcriptional regulator, GntR family protein	0	19.842	217510000
ABJ55092		conserved hypothetical protein	0	33.435	118670000
ABJ55094	tpiA	triosephosphate isomerase	0	323.31	32733000000
ABJ55098	fabZ	beta-hydroxyacyl-(acyl-carrier-protein) dehydratase FabZ	0	11.452	263990000
ABJ55105	dltC	D-alanine--poly(phosphoribitol) ligase subunit 2	0.0014771	3.7175	177490000
ABJ55106	trpS	tryptophanyl-tRNA synthetase	0	170.42	934460000
ABJ55107		ABC transporter, ATP-binding protein	0.0069444	2.0177	169990000
ABJ55108	rfbB	dTDP-glucose 4,6-dehydratase	0	323.31	11930000000
ABJ55111		TrmH family RNA methyltransferase group 3	0	26.886	110580000
ABJ55113		DegV family protein	0.0042194	2.3426	44092000
ABJ55115	pyrB	aspartate carbamoyltransferase	0	89.37	1859600000
ABJ55121	ppaC	manganese-dependent inorganic pyrophosphatase, putative	0	323.31	6478600000
ABJ55122	budA	alpha-acetolactate decarboxylase	0	7.5515	39881000
ABJ55125	dnaJ	chaperone protein DnaJ	0	13.33	24463000
ABJ55128	ddlA	D-alanine-D-alanine ligase	0	107.86	2578900000
ABJ55133	recN	DNA repair protein RecN	0.0042735	2.5521	23314000
ABJ55135		oxidoreductase, short chain dehydrogenase/reductase family protein	0	39.327	275520000
ABJ55137		phosphomethylpyrimidine kinase, putative	0	15.787	110620000
ABJ55139	murA-2	UDP-N-acetylglucosamine 1-carboxyvinyltransferase	0	61.993	570190000
ABJ55142	acpP	acyl carrier protein	0	79.192	437960000
ABJ55144	trmE	tRNA modification GTPase TrmE	0	34.929	771450000
ABJ55145	malQ	4-alpha-glucanotransferase	0	13.808	76793000
ABJ55150		conserved hypothetical protein	0	64.006	282360000
ABJ55153	frr	ribosome recycling factor	0	323.31	15436000000
ABJ55156	aroB	3-dehydroquinate synthase	0	51.993	379470000
ABJ55158	pheS	phenylalanyl-tRNA synthetase, alpha subunit	0	19.141	273160000
ABJ55165		KH domain protein	0	7.842	607590000
ABJ55166	pyk	pyruvate kinase	0	323.31	52905000000
ABJ55168		1-phosphofructokinase, putative	0	145.52	622770000
ABJ55170		membrane protein, putative	0	52.226	71347000
ABJ55175	ssb	single-strand binding protein	0	323.31	3810300000
ABJ55184		ATP-dependent RNA helicase, DEAD/DEAH box family protein	0	39.059	195280000
ABJ55189	ezrA	septation ring formation regulator EzrA	0	47.336	509780000
ABJ55190		hydrolase, carbon-nitrogen family protein	0	80.509	549500000
ABJ55191	trmD	tRNA (guanine-N1)-methyltransferase	0	5.945	94225000
ABJ55200		conserved hypothetical protein	0	14.764	339590000
ABJ55204	rpmG	ribosomal protein L33	0	160.71	6994600000
ABJ55208	trxB	thioredoxin-disulfide reductase	0	323.31	6380100000
ABJ55209	pepC	aminopeptidase C	0	323.31	4505900000
ABJ55213	rpsG	ribosomal protein S7	0	323.31	11543000000
ABJ55214	yfiA	ribosomal subunit interface protein	0	205.88	3210000000
ABJ55215		conserved hypothetical protein	0	97.142	1482800000
ABJ55218		Cof family protein	0	323.31	1077500000
ABJ55224		ABC transporter, substrate-binding protein	0.0081633	1.6287	109090000
ABJ55225	groES	chaperonin, 10 kDa	0	142.01	2631100000
ABJ55228		amino acid ABC transporter, amino acid-binding protein/permease protein	0	323.31	3360000000
ABJ55229		nitroreductase family protein	0	100.18	700060000
ABJ55234	rpmC	ribosomal protein L29	0	323.31	22272000000
ABJ55235	dnaN	DNA polymerase III, beta subunit	0	35.703	1375700000
ABJ55236	rplF	ribosomal protein L6	0	323.31	32036000000
ABJ55237	nrdH	glutaredoxin-like protein NrdH	0	46.35	159450000
ABJ55238	ndk	nucleoside diphosphate kinase	0	184.43	4260500000
ABJ55240	prfC	peptide chain release factor 3	0	81.312	1601000000
ABJ55242	gltX	glutamyl-tRNA synthetase	0	248.7	4649400000
ABJ55247		probable membrane lipoprotein TmpC precursor	0	299.16	1403700000
ABJ55248	sufS	cysteine desulfurases, SufS subfamily protein	0	75.516	215400000
ABJ55249		4-methyl-5(b-hydroxyethyl)-thiazole monophosphate biosynthesis protein, putative	0	175.14	667870000
ABJ55251		PTS system, mannose/fructose/sorbose family protein, IID component	0	199.36	2168500000
ABJ55256		conserved hypothetical protein	0	15.224	690970000
ABJ55261	gdhA	NADP-specific glutamate dehydrogenase	0	323.31	78291000000
ABJ55265		conserved hypothetical protein	0	122.25	368910000
ABJ55270		transcriptional activator, Rgg/GadR/MutR family protein	0	13.042	270120000
ABJ55271		Cof family protein	0	14.579	87840000
ABJ55280		protein jag (SpoIIIJ-associated protein), putative	0	32.116	574450000
ABJ55287		peptidyl-prolyl cis-trans isomerase, cyclophilin-type	0	158.28	239810000
ABJ55288		conserved hypothetical protein	0	290.73	1540200000
ABJ55291	gpsA	(NAD(P)+) glycerol-3-phosphate dehydrogenase	0	323.31	3791000000
ABJ55292	ftsA	cell division protein FtsA	0	223.05	2285800000
ABJ55295	fld	flavodoxin	0	139.1	562050000
ABJ55301	glyA	serine hydroxymethyltransferase	0	323.31	15956000000
ABJ55307	dexB	glucan 1,6-alpha-glucosidase	0	82.275	446440000
ABJ55311		class I glutamine amidotransferase, putative	0.0029197	3.2556	1640400000
ABJ55319	cps2D	tyrosine-protein kinase Cps2D cytosolic ATPase domain	0	12.2	212080000
ABJ55322	rplN	ribosomal protein L14	0	212.26	8649700000
ABJ55324	gki	glucokinase	0	72.625	789950000
ABJ55331	prs2	ribose-phosphate pyrophosphokinase	0	248.39	969910000
ABJ55333	pbpX	penicillin-binding protein 2X	0	5.6897	35082000
ABJ55335	cps2C	chain length determinant protein/polysaccharide export protein, MPA1 family pr	0	75.018	1009300000
ABJ55341	fhs	formate--tetrahydrofolate ligase	0	88.71	1743500000
ABJ55343		conserved hypothetical protein	0	72.732	616940000
ABJ55344	fabD	malonyl CoA-acyl carrier protein transacylase	0	234.21	2328800000
ABJ55358		SpoIIIJ family protein	0.0069541	2.0965	33536000
ABJ55361	aroD	3-dehydroquinate dehydratase, type I	0	60.89	3060500000
ABJ55365	grpE	heat shock protein GrpE	0	262.24	3710700000
ABJ55366	aroK	shikimate kinase	0	4.1969	177810000
ABJ55369		YbbR-like lipoprotein, putative	0	6.449	45890000
ABJ55371		nitroreductase family protein	0	109.19	574310000
ABJ55373		PTS system, IIB component	0	31.436	234940000
ABJ55378	cbiO1	cobalt ABC transporter, ATP-binding protein CbiO1	0.002907	3.0579	248980000
ABJ55379		protease maturation protein, putative	0	211.15	1311900000
ABJ55380		thioredoxin family protein	0	24.995	503480000
ABJ55381		phosphate ABC transporter, phosphate-binding protein, putative	0	68.757	115400000
ABJ55383	amiC	oligopeptide ABC transporter, permease protein AmiC	0	190.43	1043800000
ABJ55386	lysS	lysyl-tRNA synthetase	0	323.31	4731300000
ABJ55391	rpsU	ribosomal protein S21	0	48.57	12259000000
ABJ55393		alcohol dehydrogenase, zinc-containing	0	269.99	2283700000
ABJ55394		ABC transporter, substrate-binding protein	0	323.31	5304300000
ABJ55397	thrB	homoserine kinase	0	17.45	121850000
ABJ55398	pepO	endopeptidase O	0	323.31	17448000000
ABJ55399	ispD	2-C-methyl-D-erythritol 4-phosphate cytidylyltransferase	0	105.82	1337300000
ABJ55400	pheT	phenylalanyl-tRNA synthetase, beta subunit	0	191.65	457750000
ABJ55401	rpsI	ribosomal protein S9	0	259.06	20370000000
ABJ55402		conserved hypothetical protein	0	10.23	187830000
ABJ55403	ribF	riboflavin biosynthesis protein RibF	0	11.004	656290000
ABJ55404		conserved hypothetical protein	0	72.157	464550000
ABJ55405	ccpA	catabolite control protein A	0	323.31	3786100000
ABJ55410		oxidoreductase, putative	0	15.509	17638000
ABJ55413	infA	translation initiation factor IF-1	0	33.953	1073000000
ABJ55414	mvaK2	phosphomevalonate kinase	0.0069156	1.9935	307380000
ABJ55418		conserved hypothetical protein	0	20.669	98653000
ABJ55421	uvrA	excinuclease ABC, A subunit	0	10.148	111310000
ABJ55422		conserved hypothetical protein	0	19.013	319690000
ABJ55423	clpX	ATP-dependent Clp protease, ATP-binding subunit ClpX	0	83.302	290110000
ABJ55424	rpsB	ribosomal protein S2	0	323.31	26280000000
ABJ55425	lacB	galactose-6-phosphate isomerase, LacB subunit	0	9.6712	87589000
ABJ55427		amino acid ABC transporter, amino acid-binding protein, putative	0.0014728	3.6291	41347000
ABJ55429		conserved hypothetical protein	0	323.31	16563000000
ABJ55434	ribH	6,7-dimethyl-8-ribityllumazine synthase	0	74.998	160520000
ABJ55435		conserved hypothetical protein	0	22.793	42265000
ABJ55438		phosphotyrosine protein phosphatase	0	63.809	433090000
ABJ55445	lctO	lactate oxidase	0	323.31	17157000000
ABJ55446	rpsO	ribosomal protein S15	0	34.319	3777100000
ABJ55447	psaD	thiol peroxidase	0	306.45	11952000000
ABJ55449	prfA	peptide chain release factor 1	0	85.883	4731100000
ABJ55450	exp5	PTS system, IIABC components	0	36.83	176560000
ABJ55451	ilvC	ketol-acid reductoisomerase	0	323.31	39142000000
ABJ55452	penA	penicillin-binding protein 2B	0.0082645	1.8635	8934200
ABJ55453	deoC	deoxyribose-phosphate aldolase	0	66.701	384620000
ABJ55458	nrdF	ribonucleoside-diphosphate reductase, beta subunit	0	59.72	1040100000
ABJ55463		phospho-2-dehydro-3-deoxyheptonate aldolase	0	323.31	6660200000
ABJ55464		iron-compound ABC transporter, iron compound-binding protein	0	85.078	790070000
ABJ55468	malX	maltose/maltodextrin ABC transporter, maltose/maltodextrin-binding protein	0	197.34	1338300000
ABJ55471	galM	aldose 1-epimerase	0	18.525	1087200000
ABJ55472		DegV family protein	0	7.1859	44396000
ABJ55474		conserved hypothetical protein TIGR01033	0	323.31	2160400000
ABJ55478		serine/threonine protein kinase	0.0042674	2.5461	188070000
ABJ55481	rfbC	dTDP-4-dehydrorhamnose 3,5-epimerase, putative	0	323.31	7625300000
ABJ55484	pflB	formate acetyltransferase	0	323.31	6274900000
ABJ55485	cysS	cysteinyl-tRNA synthetase	0	11.702	279490000
ABJ55492	rplV	ribosomal protein L22	0	323.31	3876500000
ABJ55493		NADPH-dependent FMN reductase, putative	0	128.56	752070000
ABJ55495	capD	capsular polysaccharide biosynthesis protein	0	4.9373	410160000
ABJ55496	arcC	carbamate kinase	0	97.526	105450000
ABJ55497		conserved hypothetical protein	0	17.588	50789000
ABJ55501	leuS	leucyl-tRNA synthetase	0	323.31	3268600000
ABJ55504	sufC	FeS assembly ATPase SufC	0	323.31	11083000000
ABJ55505	atpA	ATP synthase F1, alpha subunit	0	238.45	3396400000
ABJ55512		RNA methylase family protein UPF0020, putative	0	5.3344	22606000
ABJ55516		MutS2 family protein	0	26.617	230370000

TM-CTR vs TM-AliA p-value(-Log)	TM-CTR vs TM-AliA q-value	TM-CTR vs TM-AliA A	TM-CTR vs TM-AliB Significant	TM-CTR vs TM-AliB p-value(-Log)	TM-CTR vs TM-AliB q-value	TM-CTR vs TM-AliB A	TM-CTR vs TM-AmiA Significant	TM-CTR vs TM-AmiA p-value(-Log)	TM-CTR vs TM-AmiA q-value	TM-CTR vs TM-AmiA A	TM-AliA. median LFQ int	TM-AliB. median LFQ int	TM-AmiA. median LFQ int	TM-CTR. median LFQ int	WT-CTR vs WT-AliA Significant	WT-CTR vs WT-AliA p-value(-Log)	WT-CTR vs WT-AliA q-value	WT-CTR vs WT-AliA A	WT-CTR vs WT-AliB Significant	WT-CTR vs WT-AliB p-value(-Log)	WT-CTR vs WT-AliB q-value	WT-CTR vs WT-AliB A	WT-CTR vs WT-AmiA Significant	WT-CTR vs WT-AmiA p-value(-Log)
0.01649101	0.980259205	0.011940002		0.361803233	0.772711744	0.081701597		0.050406578	0.954425	-0.034697533	23.966	23.9096	24.1277	23.9744		2.140237246	0.098352941	-1.30553786		1.001250607	0.499085714	-0.384631793		1.749210292
0.764187536	0.785975904	-0.615525564		0.071522819	0.81118258	0.104921977		1.029125816	0.686164706	-0.677350998	21.9352	21.5753	22.2153	21.2557		0.469522422	0.530804819	0.293481191		0.400852788	0.648818414	0.205204328		0.101735368
0.549664485	0.977285714	-0.220833778		2.790604393	0.383741007	-0.422218323		0.044967512	0.965731523	0.02082901	25.9695	26.0221	25.4489	25.5823		1.761217924	0.190876033	0.704507828		1.929961783	0.477271318	0.391718547		5.036680164
12.56167037	0	20.66704241	1	12.56167037	0	20.66704241		0.72334744	0.725484536	0.766327858	0	0	20.0165	20.6447	-1	12.81094642	0	-20.55210177	-1	13.01423233	0	-20.10196877		0
0.464800889	0.950373832	-0.298959414		1.614308419	0.310813187	0.630054474		0.031797179	0.957632653	0.048546155	23.9853	22.8819	24.0844	23.4906		3.975145997	0.107466667	-0.970102628		0.195295735	0.726145749	-0.15400219		2.322029444
0.363338086	0.950525346	-0.337831497		0.016880339	0.94937092	-0.021847407		0.669485158	0.788228571	-0.568702062	20.9035	20.726	21.1617	20.94		1.122537679	0.478048257	0.279565493		0.559120413	0.603702006	-0.228872299		0.118958789
0.330844174	0.941389474	0.442599297		0.676643491	0.398933333	0.681555112		0.170565589	0.912724771	0.187554042	21.9174	21.2935	21.8466	21.8182		0.10406004	0.765283531	0.145971934		0.842376727	0.44	-0.717441241		0.124044854
0.216777335	0.989408451	-0.293739319		0.344907326	0.59025256	0.422536532		0.199848239	0.909506631	-0.260032018	21.365	20.6845	21.2994	21.4522		0.794215871	0.388948148	0.478247643		0.131628932	0.779807763	0.122130076		1.456508292
0.157952323	0.932469314	0.055751801		0.784407225	0.583335689	0.289937655		2.258116403	0.804896175	-0.310448647	26.3418	26.2511	26.6501	26.3761		0.364417147	0.660084453	-0.160059611		1.306007789	0.520851406	0.279120672		2.083066034
0.064372659	0.942221441	-0.066000303		1.185551746	0.279042254	0.861970584		0.15799372	0.910353511	0.244554202	25.4552	24.691	25.8816	25.349		2.846981394	0.117735849	-0.836379369		2.963859186	0.378086957	-0.685589155		3.806639084
0.258154739	0.965808917	-0.218038559		0.218492866	0.725892562	0.160724958		0.567842158	0.80028866	-0.455074628	21.675	21.4567	22.2193	21.6688		2.508993939	0.313686486	-0.454089483		0.600934808	0.519343284	-0.421998978		2.857444987
0.100671558	0.935780718	-0.080453237		1.02872664	0.383801418	0.564409892		0.388076797	0.900317881	0.273493767	21.4648	21.0297	21.2249	21.4311		0.250129175	0.683763401	0.157677333		0.283032273	0.601185596	-0.308845838		0.126183346
0.842575791	0.765386667	0.628152212		1.101948298	0.372128	0.593504588		0.406612623	0.797469388	0.590542793	22.3053	22.0672	22.4177	22.7289		0.569178312	0.455814241	-0.424649874		0.36613985	0.56690566	-0.330531438		0.416682988
0.298525335	0.964675	0.629057248		0.212799805	0.644821053	0.377163251		1.27132786	0.808639053	-0.367665291	24.4548	24.7672	24.7477	24.3892		0.066458146	0.891589587	-0.044069926		0.777782936	0.515650794	0.318500519		0.119656697
0.170601529	0.963149206	-0.311959267		0.667064852	0.347145631	-1.01769193		0.177695852	0.916269113	-0.458254496	20.1846	21.1157	20.5508	19.7432		0.886286903	0.409485714	-0.427654584		0.930392536	0.501829268	0.40954272		0.456041568
0.060353479	0.97197411	0.029397329		0.120207758	0.883028037	-0.033419927		0.014030616	0.997477011	0.003089269	32.4408	32.7679	32.6616	32.7743		0.853042985	0.381969231	0.480443001		0.195466088	0.843758794	0.056722641		0.023441117
0.072059735	0.949415282	-0.039974848		0.350468618	0.685707434	0.196080208		0.361166629	0.915682353	-0.222003619	25.5885	25.5037	25.6959	25.6874		1.323983219	0.437932432	-0.34444205		0.456207259	0.64038342	-0.202421824		0.903010234
[illegible]	[illegible]	[illegible]	[illegible]	[illegible]	[illegible]	[illegible]	[illegible]	[illegible]	[illegible]	[illegible]	[illegible]	[illegible]	[illegible]	[illegible]	[illegible]	[illegible]	[illegible]	[illegible]	[illegible]	[illegible]	[illegible]	[illegible]	[illegible]	[illegible]

0.171368151	0.925713131	0.088724454	
0.414785046	0.940846939	0.124292692	
0.124414841	0.920932127	0.164846738	
0.226011568	0.926358491	-0.128598849	1
0.710696303	0.937340314	0.288467407	
0.41458141	0.940838095	-0.328338305	
0.114613488	0.945734024	0.044814428	
0.095918242	0.934976407	-0.067152341	
0.569789647	0.929848739	0.490483602	
0.437977697	0.930176	-0.578943253	
0.883507971	0.559529412	-0.917977015	
0.474484301	0.941174757	-0.10901769	
0.697358466	0.545904762	1.034738223	
0.662062625	0.962658228	-0.343659401	
0.165422219	0.971266881	-0.329442342	
14.86148725	0	-20.75979614	
0.335495509	0.939341772	0.129173915	
1.358013154	0.425647059	-0.905414263	
0.180517411	0.943788732	-0.045541128	
0.05537193	0.970976303	0.024645487	
0.158963575	0.938846154	-0.15752093	
0.407564222	0.964043127	-0.13315328	
0.333548993	0.97	0.244879723	

0.245390773	0.93410728	-0.064299583
0.105946915	0.944832451	-0.052105586
0.448362712	0.961368116	-0.149635315
0.197461849	0.942992593	0.056462288
0.417691808	0.946065116	0.315814972
0.446110503	0.966510949	-0.214747429
0.81771994	0.837557895	-0.515426318
1.109724404	0.901857143	0.3889726
0.77440848	0.756	0.64161396
0.234625646	0.924530233	-0.122886658
0.173064201	0.960566757	0.201641719
0.000646409	0.999080851	-0.000413577
0.292839241	0.954553672	-0.165239334
0.300589489	0.941016216	-0.51081562
0.227065479	0.978365759	0.359249115
1.916317184	0.775494253	0.422611237
0.974219761	0.962793478	0.117809931
0.390930499	0.958969325	-0.456322988
0.018735342	0.981830609	-0.013382276
0.544346773	0.840625	0.677400271
0.567101986	0.852483516	-0.674141248
0.432649002	0.967727273	-0.228796641
1.269472265	0.765846154	-0.505969683
0.44100566	0.957460317	0.127599716
0.413564185	0.947586854	0.320449829
0.459725388	0.96693633	-0.220551173
0.296520653	0.960011019	-0.154330889
0.505187216	0.9605	-0.351317406
0.39100692	0.966333333	0.293969472
0.135080319	0.943228464	-0.065228462
0.066037187	0.947422609	-0.057730357
0.082841536	0.944340716	-0.046404521
0.239139726	0.927825287	0.117267291
1.40115879	0.282222222	-1.111325264
0.208861205	0.983468531	0.303736051
2.447073639	0.55264	-0.568722089
1.626988132	0.956862745	0.279803912
1.040748885	0.834170213	-0.466703097
0.1034035	0.94309462	-0.069309235
16.8668266	0	-20.46519438
0.019993989	0.989528551	-0.007510185
0.206495766	0.954542056	-0.250002225
0.357027899	0.92550348	0.108051936
0.131925768	0.925700855	0.123888652
0.173005473	0.981134948	0.375589689
1.197137051	0.818067416	0.463973045
0.752513204	0.963723636	-0.186565399
0.642486123	0.981173653	0.324800173
0.152616726	0.936894837	-0.070902507
1.317453512	0.980701195	0.185313543
0.081358893	0.92825	0.069596608
0.116012574	0.924733728	0.096437136
0.0254842	0.97152381	-0.040895144
0.347741316	0.941088235	0.674234072
0.119827641	0.927121756	-0.098669052
0.075766246	0.935876138	0.078079542
0.530917071	0.956022599	-0.342241605
0.167962208	0.939747475	0.175302823
0.094077344	0.972443038	0.021926244
0.435603913	0.947822917	0.126537641
0.329692184	0.930013423	0.099366506
0.06799852	0.919788235	-0.1599919
0.109390521	0.926772358	-0.113612175
0.573985443	0.942714286	0.302207947
0.124336632	0.926695187	-0.057879766
0.34845825	0.931605634	-0.642935435
0.136146514	0.945648276	0.042681376
0.121153887	0.932737643	-0.076838811
2.542730763	0.714191781	0.444509824
1.3093603	0.453666667	-0.884036064
0.062257693	0.942274112	0.051435471
0.043306053	0.979544073	0.015155156
0.862950642	0.981168385	-0.165005366
0.136529254	0.942163569	0.063185374
1.095632791	0.961896104	-0.30037117
0.097463715	0.932754039	0.063681285
0.032132454	0.965899514	0.045164744
0.719623842	0.933889764	-0.409197172
0.154600209	0.972648968	-0.27498881
0.25183132	0.93129148	0.106675148
13.84005584	0	20.64086978
0.135229824	0.94392517	-0.040132205
0.21215404	0.92786087	-0.104173024
0.622948659	0.8568	0.530711492
0.187946193	0.979144828	0.333921432
0.085018655	0.926434783	0.293688456
0.029172387	0.973777778	-0.019194603
0.047109806	0.972683969	0.017366091
0.269421957	0.968467836	-0.178531647
0.091131872	0.926947368	0.129419009
1.228083107	0.559428571	-0.70191129
0.392188058	0.973481481	-0.265441577
0.196430331	0.923530516	0.136188507
0.316335213	0.973987097	-0.599837939
0.108357685	0.93616	-0.063866297
0.091898175	0.924721461	0.249013265
1.955944115	0.978910569	0.181083043
0.01860923	0.97481194	0.016768138
0.182578982	0.962727794	-0.220368385
0.726493334	0.976228571	0.180068016
0.533676452	0.960522523	0.266296705
0.474056018	0.936598639	-0.453584035
0.012743762	0.983506608	0.011843363
0.20498507	0.933972921	-0.069457372
0.243185848	0.972337165	-0.329175631
0.201369971	0.963410405	-0.208417575
0.632366558	0.922108527	0.43532753
0.587322551	0.983234043	-0.185018539
0.91107783	0.385	-1.381078402
0.042364158	0.972977492	-0.03189977
1.713125757	0.205818182	-1.134473483
1.578546878	0.464526316	-0.776210467
0.080264634	0.942745363	-0.044555664
0.119379844	0.928018018	-0.163754463
0.480055782	0.835137255	0.731906573
0.097099802	0.928530612	0.126256943
0.709594766	0.922601563	-0.063353539
0.077760909	0.945923875	0.051716487
0.335053744	0.940677596	0.461525917
0.15164748	0.945688266	-0.045304934
0.211116894	0.978263566	0.38893954
0.45230207	0.956088496	-0.280689875
0.107402782	0.934930403	-0.066793124
0.211457389	0.977529412	0.259430885
0.218241213	0.930369668	-0.130621592
0.32530862	0.959893617	-0.138762156
0.045528453	0.972006339	0.027000109
0.345657724	0.922847458	-0.086960475
0.174230405	0.938135266	-0.150898933
0.057271301	0.965086179	-0.032924652
0.82681324	0.978447257	-0.213984489
0.153686418	0.946548611	-0.04287529
0.861807346	0.555087719	-0.882729212
0.517121396	0.931336634	-0.114341418
0.282792894	0.948051546	0.139103889
1.305150872	0.562692308	-0.701800664
0.049473042	0.976304348	-0.020191193
0.410212216	0.969025641	-0.221916517
0.410737011	0.964802228	0.14144961
0.017841117	0.978644578	-0.01937294
0.39344372	0.976669456	-0.270533244
0.215774937	0.928131959	-0.087575277
1.552145436	0.951617978	-0.252892494
1.124100124	0.830640777	0.430559476
0.375811477	0.93636	-0.122964859
0.773048712	0.851587629	0.5171779
0.493092999	0.941035533	0.322179159
1.042007934	0.815636364	-0.492854118
0.439691678	0.928713656	0.090975126
13.14362191	0	20.19438457
0.217771754	0.942656489	0.151988665
1.469386215	0.78255814	0.453788439
0.542312378	0.929507692	-0.470568339
0.774196976	0.778684211	0.651236852
0.168966355	0.926990741	0.139937401
0.550402697	0.83354717	0.616615613
0.589254361	0.956292683	-0.35173289
0.35317981	0.925583333	-0.624847412
0.803708267	0.922897494	0.092717489
0.411861999	0.960364672	-0.14854304
0.180308205	0.928933045	-0.108132044
1.460667283	0.680742857	0.537954966
0.477523639	0.983488	-0.231817881
0.040185742	0.976287425	-0.012959162
0.730869182	0.976497925	-0.215957324
15.98742404	0	20.06310495
0.009751405	0.991067055	0.008125941
0.008151803	0.992860029	0.004794439
0.49907777	0.950711111	-0.34885025
0.301862904	0.929267257	-0.046674411
0.00335588	0.993106017	-0.005099297
0.083671106	0.94179798	0.043652852
0.007783632	0.991458213	-0.004872004
0.760869545	0.614666667	-0.842321396
12.15709035	0	20.7130785
0.101055172	0.940007449	0.072663625
0.047460664	0.94155268	-0.146490097
0.082558019	0.930699332	-0.25303936
0.018411182	0.978550376	0.018353462
0.430745975	0.970518519	-0.221042951
0.343228961	0.9325	-0.427329063
0.24393512	0.981540984	0.366378466
0.035527423	0.981222552	-0.010691643
0.290398574	0.928674157	-0.104094505
0.144245513	0.931779886	0.071557045
0.281057196	0.955988732	0.166484515
0.38910525	0.938417112	0.395174662
0.130253808	0.934144928	-0.059353193
1.385653181	0.977120521	0.14540259
0.044186179	0.947269682	0.057764053
0.043962475	0.97359633	-0.017855008
0.369225851	0.929762238	-0.594250997
1.711808914	0.55675	0.647104263
0.377548473	0.940185567	0.385956128
0.012696848	0.989985444	0.0070467
0.212119936	0.962092141	0.172334035
0.532793464	0.982591362	-0.172513326
0.036324145	0.948273333	-0.066864332
0.150340863	0.928567627	0.129276276
0.738225095	0.827259259	0.500193596
0.142052629	0.933493333	-0.072541237
0.840343927	0.953209877	-0.311938604
0.271320929	0.948758442	-0.143500328
0.626443069	0.92671875	0.438318253
1.221552446	0.553	0.824207942
0.153214539	0.922968504	-0.084682147
0.780259496	0.970721893	-0.302977244
0.140717406	0.929490196	0.126018842
0.239705351	0.983703704	-0.247987747
0.069298106	0.975473684	0.018244743
0.788275531	0.953710526	0.336099943
0.272593548	0.932125	-0.10269165
5.411558655	0.539391304	-0.499258041
0.293183825	0.93657971	-0.442910512
0.435654193	0.921837161	-0.080752691
10.55543874	0	19.99892426
0.528394188	0.940539683	0.487130801
0.760911331	0.942435208	0.103518168
0.091547268	0.979915535	-0.012223562
0.234315078	0.947059432	-0.151124001
0.46404734	0.899539823	0.628960292
0.330712358	0.979515625	-0.267139435
0.611738404	0.937820896	-0.42245547
0.077209013	0.94504584	0.046563148
0.295689849	0.985672474	0.231473605
0.019935292	0.988715543	-0.008024534
0.09353249	0.966300654	-0.02921772
1.250968509	0.781835294	-0.481241544
0.849549273	0.971615385	-0.315628052
1.229488496	0.938848485	0.331652641
0.359409122	0.937122302	0.6302646
0.015025366	0.97769697	0.026370684
0.028884373	0.971789137	-0.039090792
0.614659642	0.948433498	-0.282376925
1.652347472	0.939902913	-0.225586573
0.224859685	0.946414508	0.155396144
0.67554504	0.976751515	0.323688507
0.400122626	0.942015267	-0.595259666

[illegible]

WT-CTR vs WT-AmiA q-value	WT-CTR vs WT-AmiA Δ	WT-AliA, median LFQ int	WT-AliB, median LFQ int	WT-AmiA, median LFQ int	WT-CTR, median LFQ int
0.171153846	-0.634963036	25.0935	24.548	24.7031	24.0354
0.792179487	-0.067563693	22.1537	21.8412	22.2578	22.2806
0.168676056	0.549551328	25.3486	25.2015	25.1786	25.8014
0.998585573	0	20.4079	20.0743	0	0
0.176041237	-0.586194356	24.3916	23.3229	23.8062	23.3105
0.767484502	0.0770305	20.8067	21.4092	21.0444	21.1126
0.658745098	-0.174969355	21.5032	22.1486	22.0401	21.8846
0.1054	1.181084951	21.0199	21.7698	20.6008	21.6223
0.217680473	-0.455604553	26.2167	25.7715	26.3723	26.0988
0.170563107	-0.517406782	26.1685	26.1283	25.9433	25.4531
0.114083333	-0.775858243	21.8125	21.7797	22.2756	21.4041
0.681873239	-0.147150993	20.5736	20.7022	21.2872	20.603
0.392609626	-0.350880941	22.3045	22.0996	22.317	22.0756
0.767116203	0.077898343	24.8692	24.4733	24.6188	24.6181
0.341865889	-0.414653778	29.0859	28.3331	28.6277	28.385
0.964561605	0.007611593	32.5991	32.7897	32.7708	32.7613
0.31379021	-0.368781408	25.8358	25.8765	26.0485	25.495
0.172363636	-0.624993324	23.7728	24.0545	24.2309	23.8034
0.706977853	0.085645676	26.2533	26.0604	26.1084	26.3008
0.238102041	0.5394303	24.6004	24.8646	24.7594	24.8575
0.402805195	0.225845655	25.5654	26.2493	25.5882	25.7438
0.997175141	0	0	0	0	0
0.362483146	0.250167847	29.0864	29.181	28.7594	29.0206
0.209890909	0.73223718	22.393	22.4177	22.381	22.6267
0.153612903	1.137107213	21.5255	21.9266	20.6994	21.7255
0.332931677	0.264123599	29.8037	29.7433	29.7427	30.0973
0.287811765	-0.347515106	26.5718	26.3518	26.6891	26.3873
0.323710526	0.303244273	23.6187	23.6366	23.4099	23.6182
0.103047619	-0.84716479	23.6011	23.563	24.1956	23.356
0.493671111	0.189638138	29.3484	29.6262	29.4726	29.6718
0.242487562	0.368720373	25.9488	26.2163	26.3188	26.7211
0.607124304	0.134060224	23.2895	23.5275	23.1409	23.3994
0.172673267	-0.919825554	22.5024	21.4942	22.1141	21.6618
0.854073171	-0.043516795	23.5797	24.1886	23.5636	23.6366
0.957412446	-0.022857348	22.437	22.7433	22.466	22.5268
0.743196013	-0.072281837	29.0876	29.229	29.138	29.0967
0.0304	-1.360918999	21.4134	20.1629	21.2688	19.8043
0.283136	-0.352856	23.6133	23.7214	23.8106	23.5527
0.447990544	0.265647888	22.7943	22.6081	22.7967	23.0784
0.12172549	-1.148056348	21.9597	20.9025	21.346	20.4525
0.205730496	-0.455621719	25.816	26.256	26.3977	25.9204
0.900852459	-0.034844398	24.675	23.449	24.3999	23.7985
0.766131148	-0.076921145	24.7866	24.505	23.9873	24.2674
0.362837989	-0.313110987	20.111	20.2975	20.3347	19.899
0.394281167	0.26885128	28.0023	27.6424	27.174	27.2645
0.569632495	-0.187460264	22.4856	23.4171	23.9489	23.553
0.315786477	0.313521385	23.7391	23.9712	23.9798	24.4085
0.67384127	-0.096805573	30.3539	30.2595	30.3646	30.3797
0.315126761	0.355148633	24.0757	23.7805	24.0643	24.1686
0.429326877	-0.267445564	26.3767	26.0142	26.337	25.9343
0.933435294	-0.022476832	26.8459	27.1742	26.495	26.173
0	-20.70207087	21.0895	20.6352	20.7787	0
0.42763017	-0.289833069	23.4991	23.4118	23.1669	22.6435
0.690252159	0.099867821	29.1181	29.1354	28.9441	29.0589
0.178876033	-0.795225143	21.2813	21.2364	21.404	20.5677
0.200289855	-0.507673264	27.935	27.329	27.6224	27.2354
0.482296128	-0.245104154	20.6864	20.2074	20.8581	20.3704
0.637454545	-0.141124725	22.2652	21.4979	21.9554	21.4861
0.832942636	0.060667038	25.1602	26.1327	25.9821	25.8211
0.41377887	0.214188576	28.4561	29.1885	28.6812	28.9192
0.989671388	-0.00161012	26.6217	26.5287	26.9151	26.8904
0.272269058	0.391676903	27.093	27.3063	26.9777	27.4202
0.311267399	0.440027237	21.686	21.5188	21.6308	21.8047
0.158892308	-0.645423253	23.7826	23.2268	24.2066	23.5513
0.004666667	-1.339834213	22.931	22.9664	23.7896	22.4536
0.265518519	0.532763163	22.9679	22.5197	22.3092	22.5927
0.2345	0.67653656	21.0141	21.2616	20.4181	21.344
0.834639626	0.054448128	29.0678	29.4147	29.2908	28.8775
0.402718346	0.277463913	22.4231	23.1968	22.2865	22.6667
0.18125	-0.676204364	21.2489	21.0904	21.9057	21.5421
0.518788618	-0.175852776	21.2955	21.3565	21.577	21.3533
0.95702726	-0.010699272	24.8125	25.1396	25.0691	25.0345
0.495736842	0.178416252	24.8051	24.5266	24.7642	24.8318
0.506372591	-0.181749662	23.9965	24.2084	24.224	23.9557
0.0295	-2.939927419	22.3479	20.7449	25.7285	20.935
0.511747899	-0.215928396	24.5202	24.3288	24.3097	23.9819
0.361848739	0.355279287	24.045	24.5639	24.5638	25.0975
0.486726457	0.192550659	29.4643	30.0992	29.5164	29.9467
0.457735849	0.353902817	21.5836	21.6236	22.1648	22.3261
0.125818182	-0.981316249	22.5873	22.5202	22.8248	22.0562
0.351521368	-0.562498093	21.7824	21.5944	21.6677	21.4253
0.181965517	-0.613343875	24.8337	24.2329	25.1952	24.4708
0.840873846	-0.044382413	24.5566	24.355	24.5835	24.576
0.394293333	0.289319674	22.5045	22.5399	22.0933	22.3657
0.580068571	0.188296954	22.6503	23.3454	23.3866	23.4173
0.287015625	-0.526996613	20.4318	21.6268	20.7537	20.1129
0.264595349	0.392337163	25.4684	25.9476	25.6537	26.1633
0.218517647	0.66727066	27.0865	26.5909	26.9956	27.6488
0.236393782	-0.463761648	25.2382	24.2944	25.2122	24.8522
0.177701149	0.676997821	23.5156	23.4856	23.5851	24.0729
0.188512	-0.747820854	22.9806	22.3415	22.7461	21.7158
0.515420408	0.165335655	25.6202	25.6921	25.6395	25.7254
0.32956962	0.370404561	25.4292	25.6466	25.4011	26.1417
0.111565217	-0.96574084	24.3159	24.5392	25.5809	24.5729
0.5191643	-0.179574013	26.4499	26.2501	26.5279	26.5464
0.146137931	0.651980082	25.5764	25.8648	25.3954	26.0792
0.792877583	0.066876094	23.3558	23.5561	23.1892	23.3346
0.104484848	-0.865536054	23.8251	23.2899	24.2355	23.233
0.236484848	-0.895405134	21.6531	22.7248	22.6958	22.6194
0.230890173	0.56589667	22.9066	22.4986	21.7893	22.1191
0.331245283	0.331326803	22.7615	23.2588	22.7825	22.9709
0.280967742	-0.383115451	25.0212	25.7845	25.6323	25.094
0.149355932	0.631357511	27.7914	27.8311	27.5219	28.0274
0.100432432	-0.789927483	23.0681	23.0654	23.3848	22.7395
0.499436009	0.172457377	29.9836	30.0703	29.7129	29.8854
0.301954887	0.607791265	22.6857	23.4919	22.9206	22.8996
0.934096916	-0.016913414	28.4532	28.1726	28.4807	28.4406
0.315973064	-0.41990153	21.8061	20.8654	21.8207	21.4924
0.737010033	-0.086068471	23.5995	23.4016	23.3137	23.2106
0.217772455	-0.463531812	28.1465	28.0701	28.2576	27.9817
0.695272727	-0.138573011	25.1359	25.7184	25.0709	25.4191
0.405744898	-0.233472188	25.9299	26.159	26.1502	25.8806
0.310656934	-0.543624242	21.7836	19.9456	21.5717	21.1371
0.554901961	0.146320979	23.4119	23.2546	23.3798	23.5302
0.187460317	0.536156336	25.6528	25.7931	25.4029	25.7226
0.28336255	0.524336497	20.4127	19.7592	20.2053	20.4857
0.335721212	0.268883387	24.5024	24.5773	24.5659	24.8543
0.513435052	-0.153102239	24.9094	24.7999	24.8032	24.6933
0.336599388	-0.383191427	27.4429	27.2679	27.2948	26.8155
0.335696049	-0.429454803	20.4729	20.4687	20.1105	19.9998
0.237419355	-0.485337575	25.9345	25.6623	25.8263	25.2912
0.876282707	0.028141658	29.707	30.2611	30.0404	29.9944
0.233837838	-0.457712491	25.0093	24.4483	24.3313	23.9583
0.511496855	-0.223517418	22.6442	22.0759	22.3228	22.3804
0.318062284	0.28582255	26.0228	26.622	25.7443	26.0831
0.317414966	-0.459779104	22.1862	22.7549	22.9124	22.481
0.11106383	-0.870207151	24.6564	24.3004	24.4295	23.8502
0.514888889	0.186942418	26.1542	26.6511	26.5309	26.6665
0.235787234	-0.435382525	25.1204	25.4869	25.7454	25.2261
0.937448175	0.026014646	21.631	20.3135	21.339	21.4179
0.370410959	0.359088262	26.1809	26.8409	26.6221	26.7473
0.346585507	0.308365186	22.0079	21.866	21.648	21.8958
0.238133333	0.466305415	23.5639	23.2626	23.0318	23.4708
0.23621978	-0.666362127	21.9919	21.4134	21.4651	20.9097
0.402200514	0.256317139	27.9978	28.8062	28.3892	28.5786
0.179207207	0.628259023	28.1162	28.8421	28.5805	28.9351
0.170895238	-0.700166384	23.4503	23.5083	23.5988	23.1734
0.374514905	0.333770434	21.2055	21.2497	20.6184	20.9847
0.555217221	0.193277359	22.718	22.6302	22.6104	22.9162
0.347839542	0.309192657	24.0425	24.2977	23.729	24.0229
0.428281553	0.449105581	21.3285	21.8265	21.3612	21.6279
0.275319149	0.531288147	21.1869	20.7871	20.5513	20.9325
0.506021505	-0.278868039	21.6064	20.902	20.9379	20.5922
0.33228483	0.308816592	25.7835	26.0467	25.6071	25.9842
0.171786667	-0.621584892	28.4286	28.4544	28.7991	28.3132
0.351828571	-0.33552742	28.3063	28.6158	28.8886	28.5026
0.340214925	-0.287530581	24.275	24.6533	24.7502	24.4209
0.264962264	0.42534097	27.3285	27.3449	27.6363	27.8856
0.401926316	0.375548681	21.2988	20.9061	20.4052	20.8382
0.378522911	0.263536135	28.3035	28.441	28.1513	28.261
0.271545455	0.387888273	27.0931	27.8897	27.43	27.9669
0.288607004	-0.355197589	28.2969	28.3552	28.1526	27.82
0.481845455	0.255858103	21.4053	22.305	21.4248	21.4499
0.208394366	-0.448946317	27.7874	27.729	28.0567	27.6155
0.412565757	0.234332403	26.5849	26.6735	26.2694	26.6702
0.761331137	0.078450203	23.2763	23.5657	23.5863	23.8111
0.510635593	-0.311965942	20.9668	20.5713	20.271	20.3361
0.211754601	-0.548050563	21.8343	21.6818	21.9102	21.4303
0.163522388	0.727729797	25.0276	24.6837	23.3525	24.056
0.326588997	-0.403578758	21.045	21.0874	21.7097	20.8704
0.098344828	-0.922969818	23.3539	23.1447	23.6143	22.8887
0.270764977	0.839924494	23.6369	23.3256	22.6202	23.4001
0.937442815	-0.018770536	21.5101	21.8108	21.7789	21.8214
0.341517647	-0.347743352	22.8433	22.6414	23.0995	22.6171
0.280842105	0.421054522	23.8904	24.1273	23.7295	23.8405
0.234311111	-0.440876325	24.949	24.6348	25.1062	24.7117
0.409522843	0.301184018	23.1696	23.263	23.2641	23.3108
0.122943396	-1.14283212	20.899	21.205	20.7161	19.576
0.420829268	0.207198461	21.7199	21.7818	21.7472	21.9711
0.236834225	0.420569738	29.0347	29.1957	29.2205	29.6218
0.995768688	0	0	20.2546	0	0
0.921486726	-0.025887171	21.0378	19.9488	20.7263	20.6384
0.670452297	0.097915967	25.8602	25.6827	25.7823	25.8806
0.101538462	1.177582423	22.8635	23.4621	23.2153	24.1117
0.548748031	-0.139403343	27.473	27.7531	27.4904	27.3265
0.849815951	0.071832339	23.2146	22.4244	22.7552	22.2846
0.093481481	-0.837343534	25.0377	23.991	25.0498	24.3404
0.579908745	-0.16990598	26.2173	26.2187	26.5564	26.3328
0.512125	0.196502686	26.9064	26.8588	27.25	27.4373
0	-20.47199853	20.2641	0	20.4489	0
0.782982172	-0.05772082	27.5563	27.3608	27.447	27.2745
0.469413953	0.205844243	24.0047	24.3831	23.9097	24.2754
0.989344681	0.002305984	25.465	25.175	25.3612	25.3826
0.499530435	0.287565549	22.7188	23.0133	22.4468	22.6891
0.791317384	0.059246063	27.4611	28.0161	27.6059	27.8192
0.794787302	-0.067428589	24.2487	23.673	24.1534	23.9396
0.305697417	-0.379363696	25.9274	25.8382	26.0239	25.6784
0.195762963	0.66925621	22.4831	22.3651	21.7318	22.3011
0.740554257	0.091274261	24.9419	25.6319	24.9559	24.9001
0.478174312	0.2561903	20.6665	20.6524	20.4627	20.6876
0.172196078	-0.96730868	21.301	21.0712	21.077	19.9647
0.852439695	0.047909419	25.2602	24.8933	24.5018	24.651
0.293153846	0.343024572	25.0631	24.9662	25.0735	25.5049
0.171105263	-1.003915151	29.3877	30.1191	30.5659	28.7916
0.494	0.232971191	24.1556	24.3627	24.2738	24.248
0.429932367	0.27920473	24.5069	25.493	24.942	24.8619
0.497103753	-0.227245649	24.2995	24.5594	24.5703	24.311
0	-20.33615621	21.7767	20.6118	20.1422	0
0.559953307	-0.13499705	27.6876	27.745	27.7758	27.5967
0.315972973	-0.349855105	22.392	22.5403	23.2345	22.882
0.177527273	-0.966334343	21.6097	21.5712	21.7038	21.0219
0.316714777	0.525241216	22.1914	22.1546	21.225	21.8954
0.783735057	-0.071704547	25.5823	25.8269	25.9536	26.2214
0.818169279	0.056563059	24.682	24.8949	24.6129	25.0226
0.724196277	-0.199255308	24.1951	24.4653	23.7494	24.3024
0.625779412	0.113421758	27.2017	27.5656	27.4494	27.5323
0.263574879	0.577646573	24.1643	24.0957	23.3901	24.2688
0.726947723	-0.094984372	22.3444	22.1092	22.3131	22.5259
0.216952381	-0.464504878	22.0285	22.6075	22.8163	22.4256
0.664778761	0.138025284	22.0047	22.7003	22.4484	22.5831
0.209614458	0.448758761	27.9345	28.5088	28.1388	28.4705
0.325819355	-0.286955198	26.3416	26.0973	26.57	26.1886
0.360079096	-0.401120822	25.4874	25.3956	25.7233	25.6296
0.294550943	0.314652443	30.311	30.3718	30.1569	30.4969
0.266545455	0.346007983	27.38	27.4347	27.3055	27.6845
0.164705882	-0.795423508	20.193	20.2088	20.425	19.7712
0.177707865	-0.824100176	21.3081	21.2643	20.8663	19.9362
0.809685535	0.067391713	22.9688	22.5863	23.0777	22.6925
0.277551724	0.371816635	28.9705	29.4779	29.0793	29.3864
0.850709677	0.057711919	20.8603	20.7948	20.416	21.0845
0.251054187	-0.367651304	24.7113	24.4281	24.9002	24.5617
0.237625	-0.412587166	25.2322	24.9267	25.3105	24.9725
0.568639692	0.130859057	28.073	27.9868	27.9191	27.9867
0.08784	-0.9461387	24.477	24.0839	24.6855	23.5361
0.867010558	0.038189888	22.2126	22.1107	22.0533	22.117
0.207159236	-0.420062701	27.6906	27.6023	27.856	27.4174
0.280706827	-0.379637082	23.9302	24.5146	24.4899	24.0033
0.103222222	-0.695688248	27.5073	27.3879	27.7888	27.1717
0.482004577	-0.190382004	24.5812	23.9754	24.7436	24.5271
0.402772379	-0.346946716	20.7015	20.5702	20.7361	20.4565
0.232603352	0.743745168	21.7998	21.7701	21.9675	21.9103
0.20854902	0.465181033	26.0732	26.3411	26.3765	26.8036
0.512707368	-0.217752457	22.2448	22.6159	22.2925	21.9463
0.854480243	-0.06273524	20.661	21.2518	21.492	21.0135

0.401312336	0.242541631	26.5518	26.7531	26.5795	26.7204
0.610780037	0.127001762	27.3982	27.3959	27.1455	27.4285
0.683395431	0.121834437	22.0762	21.5711	21.3425	21.7364
0.209433962	0.644912402	21.202	20.7681	19.9634	20.8075
0.264174757	-0.394312859	24.9068	25.227	25.3564	24.9019
0.635242259	0.123628616	22.6097	23.5102	23.6716	23.6283
0.322793443	0.286287308	25.4693	25.4131	25.4311	25.7449
0.527267606	0.150108337	25.024	25.663	25.0575	25.2169
0.177674419	-0.893379529	21.7284	21.6739	21.464	20.5884
0.342432749	-0.329037348	23.2076	23.1951	23.0937	22.7549
0.576969466	0.18815581	22.1261	22.3993	22.2772	22.5952
0.264915888	-0.37705644	26.6757	26.7832	26.9217	26.5562
0.777694805	-0.108230273	22.2843	22.3005	22.188	22.0069
0.682522648	0.115846952	23.3094	23.0496	22.8205	22.954
0.40940458	0.29248937	21.9661	22.2218	21.9038	21.7896
0.784733119	0.087066015	20.7904	20.6998	20.5288	20.6258
0.150133333	0.697506587	26.7216	26.8449	26.3599	27.1295
0.066857143	-1.402689298	23.7594	23.7651	23.7448	22.6278
0.269522936	-0.420957565	27.2257	27.1041	27.6517	27.1272
0.460583529	0.194036166	27.3151	27.7454	27.3505	27.6703
0.495237251	0.253205935	21.0943	20.4625	20.0921	20.5902
0.646177215	0.125753403	24.0202	24.4845	24.2356	24.3607
0.819499218	0.056558609	24.3417	24.7012	24.4949	24.8485
0.096133333	-1.260858536	21.8713	20.8921	21.381	20.4491
0.919846381	-0.034427961	19.1381	19.1072	19.5773	18.9897
0.74359401	0.114411672	28.2571	27.3884	28.043	27.9412
0.994366197	0	20.2802	19.8277	0	0
0.926332842	0.017397563	26.9208	27.0887	26.7187	26.8146
0.475935484	0.400936445	23.6433	23.6105	22.2786	22.58
0.442552381	-0.23610719	22.0373	21.956	22.1475	21.8517
0.178216867	0.53210481	26.7223	26.6565	26.4625	26.9224
0.188458015	-0.649226189	24.0667	24.1932	24.6971	24.0564
0	20.48190848	21.4785	20.5442	0	20.3733
0.796602219	0.058732669	23.8429	23.7623	23.6182	23.6021
0.335749216	0.498166402	21.6325	20.9366	19.8139	20.7256
0.429831325	-0.330348969	25.1905	25.0041	24.1789	24.2435
0.440324582	0.236496925	21.9133	23.2343	22.4566	22.566
0.1556875	-0.62796243	24.163	23.768	24.3897	23.6872
0.648410811	-0.105402629	25.1054	25.3408	25.1996	25.163
0.703904274	0.138047854	27.0278	26.9758	26.2	26.6164
0.531029821	-0.160792033	28.9973	29.1957	29.1151	29.0952
0.339575221	-0.275554339	27.5808	27.669	27.6801	27.3065
0.832086553	-0.077585538	20.6364	21.0542	20.3706	20.426
0.346363112	0.322804133	23.5078	24.9008	23.7678	24.4409
0.125	-0.796559652	25.1264	24.9719	24.9019	24.3144
0.265657143	0.582674026	20.9591	20.8274	19.9647	20.5384
0.403759791	-0.256506284	28.4033	28.3099	28.6507	28.1455
0.374854054	0.325983683	20.3919	21.1969	20.2569	21.003
0.175020408	0.569869359	26.5643	26.4208	26.3287	26.6885
0.530661355	0.151432037	28.5701	29.0228	28.7961	28.9862
0.78383871	0.055815061	27.3436	27.4519	27.3756	27.4211
0.274208696	-0.513133049	21.6484	22.1808	22.0779	21.533
0.169580247	-0.578664144	27.2512	27.011	27.4685	26.9626
0.512090909	-0.183863322	25.6107	24.9381	25.847	25.7137
0.9698602	-0.008874575	27.9826	27.6265	27.4084	27.1824
0.798689873	0.060722987	28.164	29.0167	28.4103	28.1423
0.386959786	0.408051173	21.3981	21.1314	21.349	21.6319
0.293079848	0.426967939	21.9602	21.9788	21.4495	22.1189
0.182886957	-0.61435318	23.0747	23.0421	23.1526	22.5569
0.684859619	-0.093168894	26.4838	26.5252	26.4098	26.3124
0.282066116	0.45932134	22.2802	22.2829	22.1707	22.4455
0.345850575	0.275122643	28.884	29.3865	29.1212	29.3046
0.179728814	-0.659568787	22.442	22.5258	23.1537	22.4213
0.600648045	-0.201404254	20.2933	19.6965	20.4716	20.3866
0.306205882	0.590093931	27.0786	28.103	27.7134	27.7097
0.362519337	-0.331830661	24.5988	24.7434	25.0868	24.6287
0.178458716	-0.565948486	26.6994	25.8881	26.8675	26.3015
0.403733333	0.307393392	24.0191	24.1723	23.9855	24.0117
0.23785567	-0.644290606	20.3885	19.9251	20.2675	19.7044
0.523241935	-0.215260506	26.619	25.9005	26.3123	26.2363
0.17575	-0.626742045	22.407	23.1588	23.5952	23.211
0.590766917	0.128741264	27.0685	27.5023	27.3149	27.3977
0.235060109	0.389776548	26.8116	27.2738	26.9309	27.3781
0.651303411	0.106275876	28.0189	28.425	28.1302	28.2539
0.918065089	0.023142815	21.5986	21.6786	21.6007	21.5267
0.188909091	0.788987478	22.6135	22.2366	23.5076	24.3491
0.522769231	0.170906703	31.0147	31.3352	30.9737	31.1576
0.481150685	0.189066887	25.9712	26.153	25.8777	25.9962
0.187129032	0.528564135	26.2047	26.1283	26.0492	26.4775
0.278	0.437816938	25.4526	25.6356	25.1842	26.09
0.203539568	-0.507659276	26.6298	26.6674	26.5387	26.0732
0.072	-1.101974169	24.0597	23.5215	23.8892	23.0024
0.179697479	-0.683720271	20.5995	21.0009	21.2444	20.6704
0.363266667	-0.426611582	20.0257	19.0985	20.8888	19.9671
0.325889251	0.428754171	22.7277	22.5584	22.2313	22.6509
0.511456476	-0.37730662	22.3709	22.7832	22.4706	20.874
0.834488336	0.06654644	23.5252	23.3737	23.4661	23.6797
0.445577197	0.253278414	23.4295	23.596	23.1072	23.1895
0.364256267	-0.333868345	23.9282	23.5811	23.7795	23.5637
0.292926641	-0.497749329	21.0246	20.5483	20.6523	20.2486
0.590821764	-0.148852348	25.4703	25.7704	25.7845	25.7143
0.610762963	0.236724536	21.8792	21.3461	21.4366	21.6934
0.124642857	-0.783371925	28.611	28.6127	29.1516	28.6266
0.462685315	-0.180209478	24.717	24.784	24.8508	24.6753
0.28147541	0.38639005	25.5185	25.6871	25.6006	25.8656
0.567704854	-0.149785995	22.56	22.6558	22.5988	22.4245
0.755980198	0.070473035	23.4062	22.7661	22.5419	22.5522
0.567953846	0.158856392	28.2711	27.7795	28.226	28.3034
0.44636019	0.19829909	27.3009	27.3832	27.2807	27.4616
0.188461538	-0.600284894	24.9651	25.0508	25.2254	24.5826
0.873259036	-0.03246371	28.8651	29.4208	29.0015	28.8251
0.288913386	-0.390183449	22.3038	22.0501	22.5005	22.0311
0.326909091	0.340917905	27.1139	26.6132	26.9311	27.5188
0.264890995	-0.644920985	23.634	23.0368	23.0439	22.4779
0.401772021	-0.264243444	25.8565	26.0681	25.7326	25.5808
0.153114754	0.681409836	25.1015	25.5156	25.091	25.6264
0.208	0.502449671	26.2122	25.9368	26.3521	26.5944
0.337185185	-0.344673475	25.0275	25.4478	25.5159	24.9214
0.222744186	0.495503743	23.4867	23.403	23.0794	23.4843
0.101181818	0.70211792	29.0997	29.1322	28.8671	29.5487
0.324051282	0.349700928	21.826	21.5166	21.974	22.1653
0.790057325	0.073722204	24.0108	24.2205	24.191	24.1956
0.653971326	0.15500768	20.8016	20.5899	20.3506	20.2832
0.747609272	-0.074074427	25.3698	24.4572	24.926	24.7165
0.47543649	0.254230817	22.812	23.2585	22.908	23.3872
0.512042283	0.376302719	20.6211	19.8986	20.1392	20.8936
0.278142259	0.379138947	30.4728	30.3073	30.2625	30.9235
0.102705882	2.014746666	26.0176	24.9845	25.0626	25.6273
0.435932854	-0.374121984	23.0037	22.2435	21.0972	20.8998
0.832402477	-0.051864624	24.5568	24.7997	24.5723	24.5939
0.105365854	-0.874289831	24.8939	24.9467	25.3464	24.7699
0.230275862	-0.428856532	25.9638	25.7605	25.6092	25.3011
0.211097561	0.434607824	28.3619	28.5507	28.4809	28.8631
0.1793	-0.513717016	24.2321	23.482	23.6266	23.1362
0.556397661	0.21989282	21.1853	20.5274	20.9402	20.8547
0.733644295	-0.100652377	20.8625	20.6953	20.3459	20.3696
0.325408638	0.277311643	25.8919	25.8136	26.058	26.3362
0.324726073	-0.286037127	26.0382	25.7827	26.0735	25.7752
0.513721519	0.272535006	23.7485	24.1991	23.4507	24.3718
0.096375	-0.863916715	24.615	25.0828	25.0177	24.2486
0.683347222	0.098674456	25.0538	24.7594	24.6534	24.8318
0	20.4161272	20.7924	0	0	20.5744
0.170490566	-0.716245651	20.8791	20.7162	21.0508	20.4516
0.543263366	0.216179212	23.6307	23.341	22.7131	22.8742
0.41040201	-0.228756269	26.1237	25.382	25.8442	25.7091
0.316682594	-0.330847422	21.237	21.9276	21.6177	21.2869
0.118122449	-0.907004992	21.3538	20.5322	21.6196	20.5183
0.850073394	0.043328285	23.9642	23.4871	23.739	24.1175
0.062782609	-0.880404472	25.42	25.2345	25.9753	25.0288
0.412907731	0.240382195	24.6203	24.6715	24.9376	25.0555
0.326223642	0.366324743	23.4402	23.6181	23.4043	23.9602
0.410383838	-0.215115547	27.534	27.8543	27.8346	27.6107
0.854216134	0.03463459	25.0538	25.2166	25.1055	25.0373
0.754763636	0.063473066	27.7682	27.7757	27.6011	27.7133
0.181196581	-0.569490115	21.7986	21.6269	22.2888	21.6477
0.301378277	-0.352056185	23.8002	23.4503	24.0287	23.7859
0.183508772	0.545001666	23.0436	23.5141	23.1957	23.663
0.791763578	-0.119298935	21.7099	20.7097	20.9146	20.8526
0.850793262	-0.039903959	28.4471	28.6292	28.4811	28.2523
0.230285714	0.459889412	24.4523	24.7031	23.9709	24.4029
0.324874598	0.350152334	27.854	28.3743	28.0582	28.1661
0.373673913	0.24224472	26.5406	26.3035	26.1924	26.4371
0.785329053	0.065694491	25.7365	25.4708	25.9969	25.7953
0.104228571	0.831600825	23.4822	23.5118	22.6701	23.3626
0.171243243	0.710342407	23.5411	23.4303	23.0686	23.8167
0.212456376	-0.50979201	23.3246	23.1324	23.2648	22.7972
0.696780822	-0.144585609	22.9311	22.5622	22.9657	23.0731
0.809215748	0.046803792	27.7252	27.5398	27.6692	27.7059
0.461433255	-0.31185627	21.4621	21.192	21.0475	20.7402
0.896926756	-0.030026118	24.3332	24.444	24.3158	24.5415
0.273013333	0.451286316	21.6972	21.2563	21.4143	21.869
0.623941068	-0.158122698	20.3453	21.0652	20.4865	20.3225
0.47175	-0.263902982	22.2216	22.0927	22.2755	22.076
0.410478589	0.249392827	25.3714	24.9139	24.8359	25.0132
0.722132428	0.076622327	27.964	27.659	27.85	28.0433
0.969142857	-0.007376989	26.4096	26.1832	26.4452	26.4473
0.17264	-0.611014366	25.4397	25.6528	25.9796	25.5448
0.303059701	0.44050471	25.7985	25.5725	24.7589	25.2872
0.2054	0.838348071	20.942	20.4777	19.9939	20.5996
0.263863415	0.357684135	27.8864	27.9982	27.7059	28.0617
0.238743455	0.453022639	26.0139	26.1025	25.7131	26.0794
0.482276644	0.180701256	27.1927	26.8047	27.4517	27.6843
0.484692135	-0.294017474	22.1219	22.1219	22.2021	21.7948
0.317466667	0.649285634	21.1341	22.844	22.0367	23.0553
0.207328671	-0.509525299	24.0622	23.6198	24.2369	23.6021
0.624494465	0.129213015	22.8073	22.5125	22.3961	22.5538
0.530955823	0.210306803	24.2698	23.7777	23.3716	23.8057
0.496826374	0.266159693	21.0536	20.6367	20.8054	20.9579
0.196716418	0.535955429	24.7014	24.8609	25.0818	25.5549
0.683602094	0.096779823	25.2917	25.4276	25.5352	25.6967
0.508332623	0.173295657	27.0605	28.5063	27.6827	27.8292
0.95654023	0.012966156	22.7524	23.4296	22.942	23.0469
0.271982456	0.492079417	25.6717	26.7858	26.2397	26.3629
0.316473118	-0.378801664	21.7251	22.419	22.4465	21.9715
0.478142529	0.233410517	22.3398	22.2907	22.0354	22.2163
0.682395797	0.103569984	22.776	22.6476	22.9051	22.9293
0.314821053	0.338960965	24.7096	24.8618	24.3426	24.7539
0	21.04366811	0	0	0	21.1446
0.513160752	0.195650736	26.9192	26.824	26.908	27.0741
0.964171674	-0.007616361	23.767	24.4162	24.1455	24.2858
0.52249697	0.181058248	26.7403	26.6874	26.1531	26.2962
0.177173913	-1.8117733	21.1019	26.2463	26.6547	26.5144
0.955826087	-0.016934713	21.0329	21.5237	21.2681	21.2075
0.569135135	0.219213168	0	20.1772	20.2098	20.8086
0.634569343	0.19637235	20.7069	20.1468	20.2639	19.8491
0.88757485	-0.027826945	28.8129	28.6461	28.5961	28.6957
0.511692308	0.167941729	27.4978	27.3684	27.6294	27.65
0.660326821	0.106320699	22.1097	22.6832	22.4035	22.5444
0.546166008	-0.297551473	22.3323	21.7932	20.9162	21.0578
0.6540322	0.11108017	22.7897	23.3154	22.9591	23.0015
0.235356784	0.45111084	32.2477	32.1819	31.8254	32.5477
0.2856917	0.343947728	24.6744	25.0688	24.4205	24.763
0.092571429	-0.960349083	22.923	23.7925	23.8669	23.0137
0.343343109	0.279932658	26.3916	26.5193	26.396	26.6925
0.173365854	-0.673844973	25.3542	25.2553	25.5355	24.9845
0.634610603	-0.133804957	25.941	25.705	25.8023	25.5569
0.530308617	0.185777664	23.5835	23.5381	22.9486	23.1891
0.553571709	-0.235878627	22.4003	21.9648	22.448	22.0965
0.744	0.080963453	24.3586	24.248	24.4452	24.5733
0.684146853	-0.111977895	23.1568	23.1775	23.0236	22.9693
0.833639752	0.05061086	22.9597	23.3889	22.5988	22.6517
0.856842424	0.035433133	27.954	27.9317	27.9793	27.902
0.554351563	0.146785736	22.3383	22.3723	22.4558	22.555
0.175811765	-0.59786129	23.6581	22.6419	23.265	22.7437
0.169009346	0.529309909	27.1753	27.4818	27.145	27.6349
0.197382353	0.735469818	26.7564	26.3261	26.1823	26.4177
0.733653782	-0.071904818	30.2232	30.2314	30.3615	30.3489
0.600118959	-0.159712156	23.268	23.2267	23.5898	23.4503
0.395883905	-0.304393133	21.6867	22.6972	22.1423	22.0711
0.209387755	-0.584667842	20.2128	20.2204	20.5952	19.7585
0.496246696	0.175351461	25.591	26.2691	26.0163	26.1521
0.860405446	0.030860901	26.4164	26.2539	26.1511	26.167
0.41799511	0.279224714	24.2458	23.8058	23.1294	23.5092
0.855508346	0.05012544	24.4607	24.4988	24.2135	24.2405
0.315166078	-0.300746918	26.7326	26.9108	27.4425	27.0649
0.251215686	0.458646139	26.1617	26.342	25.8038	26.3006
0.316514286	-0.489437103	20.8664	22.4806	22.6757	22.6994
0.537484127	0.158831596	26.9581	27.1549	26.8471	27.285
0.233909091	-0.453888257	26.1258	26.0743	26.3378	26.0448
0.484099548	0.197374344	26.8937	27.7355	26.9104	27.2101
0.65919573	-0.125007312	26.4487	26.4109	26.7755	26.4249
0.462691589	-0.35684967	20.3502	19.3072	20.1857	19.5898
0.233325843	0.635027885	21.8334	21.8268	21.512	21.8059
0.833938272	-0.04902935	24.6387	25.0975	24.7087	24.7673
0.276119658	-0.389924367	26.3122	26.6677	26.7776	26.2039
0.164173913	0.850943883	23.1889	23.129	22.9458	23.6032
0.213432432	0.587972005	24.5234	24.9435	24.6748	25.1403
0.412158416	0.260395368	27.4283	27.4309	27.9547	27.935

0.341233533	-0.312041918	26.4131	26.6389	26.7631	26.3705
0.089692308	-0.945716858	29.1894	28.4974	28.7541	27.8787
0.799235387	-0.055495898	25.4966	25.0517	25.5416	25.6562
0.403458333	0.22421519	30.917	30.831	31.0737	31.3402
0.312072464	-0.332075437	27.4258	27.5763	27.7282	27.3659
0.762453947	0.082303365	23.7854	24.208	23.5918	23.5677
0.430307692	-0.273951848	25.1891	24.4852	24.7203	24.4868
0.288465116	0.439773877	24.8548	24.7167	24.4057	25.1236
0.413253731	-0.289974213	24.6739	24.1003	24.6848	24.3664
0.184	0.550059319	23.3405	23.8425	23.6133	24.1886
0.402020619	0.395770391	23.473	23.7166	23.1532	22.9945
0.569697674	0.142868996	28.4292	28.2588	28.0443	28.0967
0.494325167	-0.186386426	23.7526	23.934	23.8732	23.6086
0.278638655	-0.537235578	23.306	23.2527	23.5732	23.2559
0.576267686	0.222173691	23.388	24.5735	24.161	24.2226
0.172740741	-0.73576355	22.9601	23.1503	22.8951	22.6757
0.31670903	0.30466938	30.0867	30.4149	30.361	30.6596
0.763218391	0.087635676	21.2855	21.5317	21.432	21.1897
0.181486726	-0.531677246	24.9375	24.8115	25.1222	24.6426
0.144631579	-1.279673576	22.1602	22.0414	21.9117	21.2034
0.49600885	-0.358723958	21.4777	20.6131	20.3328	21.1584
0.337190184	0.319357554	22.2085	22.7238	22.6733	23.0384
0.206	0.46925958	22.9047	22.7296	22.598	23.0302
0.531081836	0.17652448	28.1397	27.9507	27.8646	28.3796
0.956380952	0.011819204	23.8651	23.6061	23.6055	23.4617
0.172876712	-0.64191405	24.918	24.605	24.6552	24.2002
0.191338346	0.641487122	24.2629	24.2021	23.6806	24.5656
0.317806897	0.393899282	21.1204	21.1981	20.8711	21.1373
0.273022026	-0.430129051	21.723	21.0578	21.7085	21.224
0.916172363	0.020547867	26.7199	26.1011	26.4686	26.4874
0.897504478	-0.0282348	24.829	24.0008	23.9825	23.8989
0.515270492	0.175735474	28.1407	28.697	28.2433	28.537
0.341801802	0.270711899	27.2941	27.2356	27.232	27.4132
0.171189873	0.898129463	21.7205	22.1814	21.21	22.0133
0.176227273	-0.655167262	21.8647	22.1069	22.3029	21.5899
0.282058091	0.351045926	28.8898	28.8963	28.6768	29.0249
0.94825	0.013584455	23.8153	23.7864	23.9291	23.967
0.461089202	0.281456312	22.0736	22.0078	21.923	22.417
0.248019802	0.38861688	30.8512	30.9298	31.0598	31.4287
0	19.88299211	20.7806	20.2185	0	20.1761
0.315208333	-0.348522822	28.8304	28.6565	28.8029	28.2038
0.414162963	-0.336290995	24.9404	23.9595	23.7408	23.802
0.784621359	0.061175346	28.8977	28.5888	28.7391	28.8725
0.910505952	-0.032177607	23.3267	23.7947	23.4604	23.5005
0.305777778	0.547798475	23.5307	23.6268	22.6943	23.6466
0.31652349	0.335110029	24.8053	24.8152	24.4408	24.913
0.183770492	-0.595934868	23.8569	23.7166	23.8357	23.1721
0.102421053	-0.702577591	25.7136	25.5592	26.1632	25.3763
0.334825	0.347679774	25.5838	25.805	25.1896	25.6
0.239195767	0.376716614	27.8782	28.068	27.9334	28.2868
0.579635674	-0.188814163	22.5805	22.6999	23.0352	22.3843
0.175784946	-0.683919271	23.2423	22.9645	23.2147	22.5736
0	20.1897157	20.3319	20.0998	0	20.1479
0.506837607	-0.247487704	23.2933	23.5474	23.9488	23.4837
0.212	0.606096903	22.3845	22.6078	22.4236	22.7504
0.340544379	0.343574206	24.0111	24.4102	24.0354	24.3709
0.78331723	-0.084569613	20.8237	20.3054	20.2413	20.5723
0.83505919	0.055227915	23.7426	23.9496	23.5179	24.07
0.353465909	0.285069466	29.0492	30.045	29.9389	30.1906
0.338561934	0.267808914	26.4138	26.5667	26.1988	26.4866
0.7909376	0.056563695	28.8556	28.8904	28.9446	29.181
0.531944	-0.296516736	22.4994	21.2066	21.0088	20.3306
0.040631579	1.134751956	26.267	26.7628	25.7745	26.9582
0.972467236	0.006533941	21.997	22.7974	22.1719	22.1988
0.187181102	-0.517935435	26.0657	26.2693	26.631	26.0949
0.436057416	0.439674695	22.0493	21.0986	20.8339	20.9932
0.271113122	0.421047846	28.7176	29.128	28.8326	29.3853
0.18871875	0.555542628	23.8035	24.515	24.1125	24.7849
0.272384279	-0.477360725	23.793	24.3128	24.3584	23.4577
0.832979969	-0.056025823	20.1922	20.032	20.6363	20.4766
0.888528529	0.034778913	25.0683	25.0792	25.0716	24.9056
0.312836237	0.359816869	28.0653	28.0988	27.8832	28.0261
0.591403774	-0.155276616	24.8497	24.5972	24.8148	24.5319
0.363080332	-0.248784065	30.1426	30.1626	30.2649	30.0317
0.801697161	-0.051607132	28.0603	28.2064	28.0247	27.9979
0.346959538	0.318119685	23.6979	23.9347	23.4427	23.8369
0.165371429	-0.80210336	21.6017	21.2925	21.9348	21.2532
0.515353783	-0.179687818	23.2476	24.1308	23.8888	23.5435
0.686961938	-0.142798106	22.0829	21.6293	21.5238	21.2751
0.83188125	0.045672417	25.9558	25.6141	25.7019	25.9313
0.22154386	-0.637765884	20.567	20.187	20.922	20.1955
0.937590643	0.014388084	25.8627	26.0157	25.8741	25.8737
0.335829268	0.297960917	32.3976	32.6473	32.4062	32.8458
0.512604555	-0.220440229	21.1765	21.6093	21.6513	21.354
0.505434125	-0.263981183	21.5649	21.2504	21.7734	21.4814
0.6473902	-0.335525513	23.3105	21.4268	22.0463	20.4394
0.281166667	0.394966761	25.8492	26.0637	26.0211	26.7102
0.206759494	0.466380119	28.7467	28.92	28.7447	29.095
0.208246575	0.552752495	25.1962	25.4406	25.2594	25.872
0.727891892	0.137148539	21.6106	21.9279	21.782	21.9875
0.576850575	-0.175203641	21.5098	22.119	21.3287	21.1471
0.316420339	0.341190656	29.3708	29.257	29.4504	29.9925
0.629794872	0.213629405	22.348	22.972	22.5019	21.9618
0.161333333	-1.423704783	21.7472	21.7754	22.7519	21.3461
0.233898305	0.449033419	24.8198	24.6035	24.4354	24.9638
0.956473988	-0.020781835	21.2693	22.9133	22.3029	22.3795
0.664765957	0.091777166	28.2729	28.0622	28.1307	28.1755
0.400387435	-0.349245707	21.65	21.7874	21.2618	21.2126
0.682622609	-0.147851944	20.6855	21.2739	20.9258	20.4458
0.325576159	0.282895724	25.2957	25.9478	25.1969	25.4792
0.263096154	0.453971863	27.1836	27.1096	27.1773	27.6738
0.156698413	1.120231628	21.2605	21.0144	19.7673	21.0312
0.213075	-0.504517237	22.8364	22.6071	22.7714	22.3772
0.199240876	0.462053617	25.801	25.9684	25.6801	26.1367
0	20.08089066	20.1575	20.1704	0	20.0209
0	20.46912766	20.8751	0	0	20.5119
0.176747253	0.87945811	21.8101	21.5068	20.6645	21.885
0.508629787	-0.178909938	24.1144	23.6769	23.6888	23.5751
0.0602	-1.742330869	23.5144	24.1022	24.1926	21.6909
0.629313761	0.140392303	24.7397	24.6395	23.9683	24.1518
0.591954545	-0.21197478	22.164	21.5726	20.9016	20.4294
0.362861972	0.342518489	22.8389	22.8399	22.4278	22.8202
0.417803922	0.222816149	28.3013	28.3347	28.4771	28.6644
0.958489914	-0.015012741	23.0408	22.5163	22.4353	22.9298
0.266122066	-0.518999418	22.8367	22.7432	23.0942	22.5939
0.592996255	0.190969149	23.4474	23.8034	23.9502	23.9786
0.772982114	0.061378479	30.5283	30.2994	30.5006	30.4345
0.74	0.080304464	23.9198	23.7004	24.0807	24.1551
0.11752	-0.997850736	20.4809	21.2663	21.2091	20.5909
0.276536297	0.393455505	26.3303	26.3632	25.9069	26.4289
0.49101566	-0.278146426	22.8845	22.3563	21.6284	21.6637
0.206923077	-0.50518163	24.3944	23.2558	23.8821	23.4617
0.510878661	0.1996816	23.3243	24.0112	23.6298	23.7516
0.413300493	-0.483559291	21.3538	20.8521	21.6733	21.1924
0.103069767	-0.817782084	23.1068	22.4785	23.2473	22.6224
0.591954631	0.151520411	27.1331	27.853	27.3475	27.3941
0.272547945	0.415854454	27.2027	27.1256	27.1705	27.4625
0.497601751	-0.192488035	26.84	26.8915	26.9491	26.8906
0.010153846	-1.229288101	27.7657	27.4746	28.4922	27.3138
0.506128755	-0.211798032	26.3275	26.7606	26.5133	26.349
0.332	0.316121101	25.1266	25.2624	25.0341	25.5387
0.326305732	0.316369375	24.6051	24.79	24.4222	24.8699
0.212493827	0.661101977	21.4793	21.6896	21.4665	22.2014
0.371311475	0.242060026	25.8968	26.0275	25.9224	26.1258
0.205268966	-0.461627007	26.1601	26.1572	26.6295	26.0901
0.485182844	-0.200520198	24.7548	24.1428	24.0907	23.9621
0.305888476	0.344521205	28.6955	29.0032	28.887	29.2498
0.174141414	-0.612978617	25.0073	24.522	25.4571	25.1249
0.954403483	-0.012952169	29.5836	29.4176	29.7986	29.7667
0.272125	0.42550691	21.4194	22.5916	21.5016	21.9406
0.209921053	0.468805949	26.3021	26.4027	26.4869	26.8853
0.34041543	-0.324618975	25.7194	25.2986	25.7731	25.4878
0.689924138	-0.107555072	27.0561	26.7065	26.7965	26.8418
0.412421053	-0.216081937	24.7405	24.9717	24.8813	24.6825
0.353382436	0.346288681	21.5089	21.7683	21.3964	21.6688
0.727434343	0.10439841	23.9065	22.8762	23.8789	24.0529
0.213515528	-0.477097511	25.4138	25.7797	25.932	25.4042
0.393255319	0.298047384	23.4189	24.0153	23.7977	24.168
0.41334	0.215006828	30.7485	30.7075	30.8615	31.1601
0.947568513	0.019892057	23.6532	23.8341	23.7019	23.8811
0.957847482	0.011617978	22.0401	22.5434	22.7861	22.731
0.485666667	0.184868177	21.4375	20.9396	21.1338	21.4031
0.1725	-0.754111608	23.9078	24.0785	23.7648	22.9611
0.040470588	-1.414115906	22.3035	22.3113	23.2215	22.033
0.887688156	-0.032802264	25.2032	25.417	25.4908	25.3048
0.694962199	-0.115242004	26.7063	26.492	26.3599	26.2789
0.171076923	-0.70594883	26.0784	25.9799	25.5092	25.2107
0.177619048	-0.919648488	24.0064	23.8722	23.661	22.4362
0.768019608	0.063757896	28.3654	28.408	28.6735	28.6334
0.771374593	0.084186554	23.7207	22.9146	23.7007	23.9556
0.647148014	0.139201164	25.1209	25.0088	25.1861	25.2804
0.16905	-0.978354772	22.5891	22.3858	23.3027	22.1355
0.293869732	-0.345780691	27.5796	27.697	27.707	27.3599
0.345453488	-0.290033658	24.5347	24.3243	23.9659	23.536
0.100977778	-0.704456011	24.6825	24.3002	24.6761	23.9752
0.983937411	-0.005911191	0	19.9142	20.8814	20.5766
0.177911111	-0.566650709	24.5187	24.8908	24.7149	24.2701
0.984511364	-0.003007571	25.4563	25.3487	25.3794	25.3466
0.312129496	0.374967575	23.795	23.5413	23.7795	24.044
0.917632047	0.021214167	26.0397	25.9193	25.8323	25.7859
0.280682927	-0.43617185	20.5074	20.1836	20.528	20.0678
0.519356415	0.180759112	26.4091	26.3086	26.5955	26.7845
0.317739726	0.348087947	26.8768	26.6752	26.2819	26.8408
0.592650467	0.274023374	21.3227	22.2789	22.2277	22.1249
0.207380645	-0.604746501	20.9433	19.9341	21.1241	20.3442
0.292272727	0.404314041	23.8505	23.2802	23.6543	24.2027
0.333931464	0.646006584	22.431	23.1359	21.1721	22.7314
0.175106383	1.47760582	21.0754	23.77	20.0501	23.5704
0.293923664	-0.388666153	24.8472	24.8803	25.2766	24.8319
0.211311258	0.598738988	23.6475	23.6664	23.0955	23.7793
0.326143791	-0.587741216	21.5329	21.3316	21.5824	21.0203
0.269927928	-0.369621277	25.1591	24.9999	25.5474	25.0842
0.647231884	0.10415713	26.9686	27.4755	26.9007	27.0224
0.500112554	0.193775495	27.9436	28.3652	27.8553	28.1992
0.695800344	0.090819359	25.5195	25.7009	25.4084	25.5483
0.121851852	-0.672753016	26.9088	27.0805	27.1414	26.5895
0.49762963	0.297797839	21.5601	22.1747	21.2568	21.6668
0.704559727	-0.087718646	28.658	28.681	28.7682	28.602
0.546287968	0.172712008	25.0849	24.7643	24.9315	25.0285
0.863444109	-0.038777033	23.6836	24.042	23.3335	23.3018
0.683501754	0.112150192	28.7325	29.3033	28.7061	29.0642
0.176884211	-0.669188817	21.6555	21.3757	22.4456	21.8674
0.658714286	-0.125463168	23.7986	23.7358	23.8878	23.9371
0.372435967	-0.336117744	23.3077	23.6453	24.1222	23.4958
0.504353448	0.168219884	26.2978	26.3872	26.3637	26.592
0	19.65537453	20.7326	0	0	19.575
0.313054152	-0.309372902	24.5165	25.0272	25.1453	24.8588
0.315347518	-0.893714269	22.1403	21.7118	21.5236	21.1471
0.310632727	-0.454555829	21.6327	19.9844	21.2071	20.7142
0.286095238	0.542877197	21.9791	21.9066	21.4616	21.7111
0.515474333	0.182091077	22.6147	22.659	22.7997	23.0618
0.378537634	0.260721207	22.8522	22.7043	22.5038	22.6923
0.33776	-0.282437325	29.2642	29.4474	29.3034	28.954
0.936158126	0.021958669	21.6409	20.9087	21.3443	21.3774
0.022	-1.483622551	20.7108	21.1656	20.9887	19.5794
0.281697959	0.473537763	28.2618	28.7107	28.3991	28.814
0.237127072	-0.693148931	22.133	21.1575	21.4321	20.8636
0.341036145	-0.365404765	21.04	20.4217	20.3138	20.1299
0.916983704	0.024656614	23.4042	23.3494	23.281	23.3809
0.276360515	0.392155647	29.02	28.8071	28.4768	28.9739
0.408567089	-0.253877004	26.3353	26.3542	26.4202	26.431
0.238463158	0.416516304	27.849	27.9943	27.9893	28.4709
0.497519651	-0.177095095	27.1961	27.2463	27.5987	27.3331
0.570357006	-0.226796786	21.6454	22.5576	22.0055	21.1852
0.32855873	0.256045659	29.5858	29.7222	29.78	30.0539
0.513294606	-0.238694827	19.4887	19.935	20.2869	20.2126
0.39605291	-0.262760162	24.1407	23.3113	23.3741	22.9795
0.723640678	0.08632342	24.9086	24.8172	24.5812	24.4811
0.272300885	0.334088643	26.9829	27.2898	27.0783	27.417
0.23908	-0.503925641	24.9158	23.4318	24.1301	23.5463
0.094967742	-1.154237747	25.8756	24.2469	25.058	23.9422
0.041333333	2.168667157	23.5947	24.3266	23.787	25.0705
0.591679104	0.198779106	20.7155	20.4038	19.9752	20.3224
0.736824121	-0.081309319	25.7527	25.5007	25.666	25.62
0.339428571	0.444835027	22.1751	22.259	22.0257	22.2835
0.27942616	0.369581858	27.3972	27.5885	27.3066	27.7107
0.362049587	-0.283719699	27.7573	27.5768	27.3972	27.1939
0.709945578	-0.143258413	22.7577	23.2303	22.9445	22.3479
0.064727273	-1.000637054	26.5136	26.2269	26.6945	25.7047
0.818342229	0.063650449	23.9462	23.4752	23.4796	23.6696
0.23764467	-0.55745856	21.5539	22.8422	23.3603	22.9576
0.282419753	-0.547313372	22.3383	20.8706	21.7908	21.6061
0.949630277	0.019228299	21.66	21.0125	20.573	20.4924
0.365736264	-0.255560875	26.5594	26.4598	26.5251	26.397
0.590613936	0.132397016	27.8254	28.0472	27.927	28.2199
0.647899281	-0.110036532	26.3664	26.6148	26.1576	26.086
0.187503876	-0.670082728	20.0071	19.6981	20.4302	19.5973
0.472408353	-0.438332876	21.7942	22.6278	22.4912	22.56
